# Supplementary material for: Discovery of FoTO1 and Taxol genes enables biosynthesis of baccatin III
Source: Nature. 2025 Jun 11;643(8071):582–92. doi: 10.1038/s41586-025-09090-z (PMC12240809; doi:10.1038/s41586-025-09090-z)
Supplement: Supplementary file 1 — This file contains Supplementary Notes 1–5, Supplementary Tables 1–13, Supplementary Figures 1–22, NMR spectra (Supplementary Figures 23–65) and Supplementary References. [file 41586_2025_9090_MOESM1_ESM.docx]

**Supplementary Information**

**mpXsn reveals FoTO1 and Taxol genes, enabling baccatin III biosynthesis**

Conor James McClune^1,6†^, Jack Chun-Ting Liu^2†^, Chloe Wick^1^, Ricardo De La Peña^1^, Bernd Markus Lange^3^, Polly M. Fordyce^4,5^, Elizabeth S. Sattely^1,6*^

^1^Department of Chemical Engineering, Stanford University, California 94305, ^2^Department of Chemistry, Stanford University, California 94305, ^3^Institute of Biological Chemistry, Washington State University, Pullman, Washington 99164, ^4^Department of Bioengineering, Stanford University, California 94305, ^5^Department of Genetics, Stanford University, California 94305, ^6^Howard Hughes Medical Institute, Stanford University, Stanford, California 94305

†These authors contributed equally

*Corresponding author: Elizabeth S. Sattely (sattely@stanford.edu)

**Table of Contents**

[**Supplementary Note 3**](#_heading=h.ifdw8nzd4en4)

[**Supplementary Table 7**](#_heading=h.re1reeq133ag)

[**Supplementary Figures 33**](#_heading=h.deedj8zbkelv)

[**Supplementary Figures - NMR spectrum 55**](#_heading=h.obe6hye04x6c)

[**References 98**](#_heading=h.fiv9dqcdubof)

# Supplementary Note

**Supplemental Note 1**

Structural characterization of **2’d**

We have previously observed **2’d** (compound **9** in ref.[^1^](https://paperpile.com/c/CAQ5G4/vB77)) as the main product when taxadien-5α-ol (**2**) is fed to *N. benthamiana* expressing T5αH[^1^](https://paperpile.com/c/CAQ5G4/vB77), suggesting that it is a secondary oxidation that occurs when T5αH incubates with its product **2**. Here, we confirmed this, through purification from *N. benthamiana* leaves expressing TDS, T5αH and FoTO1. 1D- and 2D- nuclear magnetic resonance (NMR) analysis indicate that **2’d** is 4β,20-epoxy-taxadien-5α-ol (**Fig. 3a**, **Table S3**). It is notable that the stereochemistry of the 4β,20-epoxy in **2’d** has not been previously reported in any natural taxanes, which exclusively features a 4α,20-epoxy stereochemistry. It is possible that the formation of **2’d** is due to overoxidation of taxadien-5α-ol (**2**) that accumulates to an unnaturally elevated level by T5αH in this partially reconstituted pathway.

**Supplemental Note 2**

TAT is a bi-functional acetyltransferase

It is worth noting that the reconstructed pathway to taxusin (**6**) required three acetyltransferases despite four acetoxyl groups on this molecule; these data suggest that one of the acetyltransferases is bi-functional. Experiments involving the feeding of **4** to *N. benthamiana* leaves expressing T9αH-750C with either TAT, or DBAT revealed that TAT is likely a bi-functional enzyme that introduces the acetyl groups on both the C-5α and C-9ɑ hydroxyls (**Extended Data Figure 4c**). This is consistent with previously reported acetylation activities of TAT on C-9 and C-10 hydroxyls.[^2^](https://paperpile.com/c/CAQ5G4/z8vLh)

**Supplemental Note 3**

Activities of T5αH and T13αH

T5αΗ has long been considered the first oxidative enzyme in the baccatin III/Taxol biosynthetic pathway, responsible for 5α-hydroxylation. However, our results demonstrate that T5αΗ is not an essential component of our reconstituted biosynthesis of baccatin III in *N. benthamiana* (**Fig. 5i**). Surprisingly, we observed that T13αH can complement a T5αΗ dropout, acting as the first oxidative enzyme and installing the 5α-hydroxylation (**Fig. 5i-j**, **Extended Data Figure 8**). Intriguingly, while both enzymes can perform 5α-hydroxylation, they each show unique activities beyond that: In the absence of FoTO1, both T5αH and T13αH produce OCT (**2’a**) and iso-OCT (**2’b**) as major products (**Fig. 3d-e, Extended Data Figure 8a**). This suggests that both enzymes likely employ an epoxidation mechanism, as it has been previously shown that OCT (**2’a**) and iso-OCT (**2’b**) can be derived from rearrangement of 4α,5α-epoxy-taxadiene.[^3^](https://paperpile.com/c/CAQ5G4/0Mifs) When FoTO1 is present, T5αΗ yields taxadien-5α-ol (**2**) and 4α,20-epoxy-taxadien-5α-ol as the dominant products (**Fig. 2d**) and T13αΗ generate taxadien-5α,13α-diol and 4α,20-epoxy-5α-hydroxy-taxadien-13-one as the major products (**Extended Data Figure 8a-b**). Isolation of these epoxide products (**Table S3, S8-9**) further supports the epoxidation mechanism of T5αΗ and T13αΗ.

It was found that, under more acidic conditions, 4α,5α-epoxy-taxadiene preferentially rearranges to taxadien-5α-ol (**2**), suggesting that 4α,5α-epoxy-taxadiene can be the potential intermediate during T5αH/T13αH oxidation.[^3^](https://paperpile.com/c/CAQ5G4/0Mifs) Based on this, one can hypothesize that FoTO1 creates a more acidic microenvironment or directly donates protons to guide the rearrangement of the epoxide intermediate. This can be achieved either through allosteric interaction of FoTO1 with T5αΗ/T13αΗ or channeling the epoxide intermediate to FoTO1’s active site. Interestingly, T5αΗ exclusively produces taxadien-5α-ol (**2**) when taxa-11(12),4(20)-diene (iso-taxadiene) is used as the substrate, which supports a radical mechanism.[^4^](https://paperpile.com/c/CAQ5G4/popoc)[^5^](https://paperpile.com/c/CAQ5G4/mYWpv) However, our discovery of the 4α,20-epoxide products suggests that the exo-alkene in iso-taxadiene can undergo epoxidation by T5αΗ and T13αH. Future feeding studies using purified iso-taxadiene and taxadiene could further elucidate the catalytic mechanism of T5αH and T13αH as well as their interplay with FoTO1 and other Taxol biosynthetic enzymes.

While 4,20-epoxy group is common among taxanes, all previously isolated epoxy-containing taxanes from *Taxus* have a 4**β**,20-epoxy configuration.[^6^](https://paperpile.com/c/CAQ5G4/9Jibm) This suggests that the two 4**α**,20-epoxy products we isolated in this study are likely not naturally produced in *Taxus* plants but are artifacts of enzyme over-oxidation in the *N. benthamiana* expression system. This hypothesis aligns with our previous findings that over-expression of T5αH in heterologous hosts led to the accumulation of over-oxidized products.[^1^](https://paperpile.com/c/CAQ5G4/vB77) In addition, the 4**α**,20-epoxy configuration would preclude the formation of the classical oxetane ring with a 5**β**,20-epoxy. Thus, our characterization of the 4**α**,20-epoxy stereochemistry in the two taxanes indicates that these molecules are unlikely to be precursors to natural taxanes that contain an oxetane moiety.[^7^](https://paperpile.com/c/CAQ5G4/zExWv)

Proposed model of taxadiene oxidation by T5αΗ and T13αH:


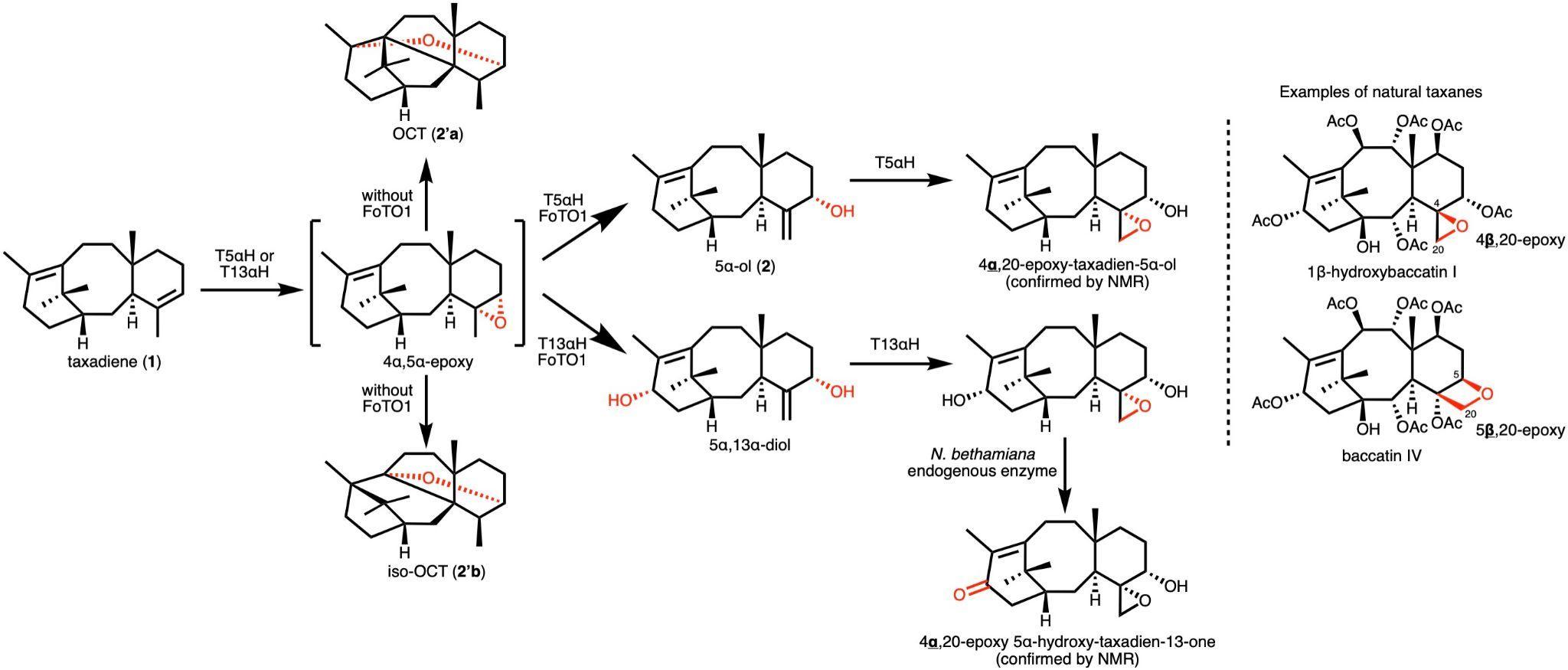


**Supplemental Note 4**

Note on FoTO1/NTF2-like domain

Further investigation is needed to dissect how FoTO1 leads to such profound improvements in the activities of *Taxus* oxidases T5αH and T13αΗ. Co-immunoprecipitation and *in vitro* binding data (**Fig. 3i-k**) suggest the role of direct protein-protein interactions with pathway enzymes, analogous to protein scaffolding role of NTF2-like domains in Ras GTPase activating protein (RasGAP) signaling[^8^](https://paperpile.com/c/CAQ5G4/oJLCS). While the few characterized NTF2-like proteins in plants have roles in protein shuttling unrelated to specialized metabolism[^9,10^](https://paperpile.com/c/CAQ5G4/KCC0a+2j1II), they differ from FoTO1 in sequence and domain architecture. FoTO1 represents not only the first example of an NTF2-like protein implicated in plant metabolism, but also the first characterized member of a gene family that appears widely conserved in land plants (**Fig. 3f**). Beyond the plant kingdom, NTF2-like proteins have independently evolved catalytic activity in fungi and bacteria[^11,12^](https://paperpile.com/c/CAQ5G4/X0YD8+Zl1eg) and have also been computationally engineered into synthetic luciferases[^13^](https://paperpile.com/c/CAQ5G4/ioaFb). Therefore, we should be cautious with functional extrapolation, given the propensity for this small, conical fold to adapt diverse roles. Regardless of its mechanism, FoTO1 has uncharacterized homologs throughout land plants and may represent a conserved family of plant proteins with valuable metabolic roles (**Fig. 3f**). It is likely these proteins will be critical for the engineering of complex pathways such as Taxol into heterologous hosts such as yeast for molecule manufacture.

**Supplemental Note 5**

We isolated a fungal species from our *Taxus* needles and included it as a component of our elicitation. We amplified the ITS2 sequence and found it matched *Aspergillus tubingensis* NRRL 4875. *A. tubingensis* and other *Aspergillus ssp.* have been previously identified from *Taxus* tissues [^14^](https://paperpile.com/c/CAQ5G4/dK2nc).

ITS2 sequence:

TGAATCATCGAGTCTTTGAACGCACATTGCGCCCCCTGGTATTCCGGGGGGCATGCCTGTCCGAGCGTCATTGCTGCCCTCAAGCCCGGCTTGTGTGTTGGGTCGCCGTCCCCCTCTCCGGGGGGACGGGCCCGAAAGGCAGCGGCGGCACCGCGTCCGATCCTCGAGCGTATGGGGCTTTGTCACATGCTCTGTAGGATTGGCCGGCGCCTGCCGACGTTTTCCAACCATTTTTTCCAGGTTGACCTCGGATCAGGTAGGGATACCCGCTGAACTTAAGCATATCAANANNCGGAGGAAGACAAGATCGGTCTGGCCGCCACCGCCTCCACCACCTATGGCGAGCCCTATCACACCGCCCGCGCCTTCGCGTCCATCGACCATCTGAGCCACGGCCGCGCGGCGTGGAACATCGTCACCACCTCCTATGCCCGCACGGCGGCGAATTTTTCCAAGAGCCATCCCGAGCATGACGAGCGTTATGCCGTGGCGGAAGAATATGTAAACGTCGTGCGCGGCCTGTGGGACAGCTGGGACGACGATGCCTTTGTCAAGGACAAGCAGGCTGGGCGTTATGTCGATCCGGAAAAGGTCCATATCCTCGACCACGAAGGCAAATATTTCNCGGTCAAAGGCCCACTCAACATTCCGCGCTCGCCGCAGGTTCACCTACGGAA

ITS primers:

| fwd ITS1 | TCCGTAGGTGAACCTGCGG |
| --- | --- |
| fwd ITS2 | GCTGCGTTCTTCCATCGATGC |
| fwd ITS5 | GGAAGTAAAAGTCGTAACAAG |
| rev ITS3 | GCATCGATGAAGAACGCAGC |
| rev ITS4 | TCCTCCGCTTATTGATATGC |

# **Supplementary Table**

**Table S1.** List of genes used in this study.

|  | **Acronym** | **Other name used in this study** | **Full Name** | **Protein family** | **Ref.** |
| --- | --- | --- | --- | --- | --- |
| 1 | TDS | -- | taxadiene synthase | terpene cyclase | [^15^](https://paperpile.com/c/CAQ5G4/QYnjU) |
| 2 | T5αH | -- | taxadiene 5α-hydroxylase | P450 | [^5^](https://paperpile.com/c/CAQ5G4/mYWpv) |
| 3 | FoTO1 | -- | facilitator of taxane oxidation | NTF2-like | This work |
| 4 | TAT | -- | taxadien-5α-ol-*O*-acetyltransferase | BAHD acyltransferase | [^16^](https://paperpile.com/c/CAQ5G4/yqB7Y) |
| 5 | T10βΗ | -- | taxane 10β-hydroxylase | P450 | [^17^](https://paperpile.com/c/CAQ5G4/34e0G) |
| 6 | DBAT | -- | 10-deacetylbaccatin III-10β-*O*-acetyltransferase | BAHD acyltransferase | [^18^](https://paperpile.com/c/CAQ5G4/1dRiL) |
| 7 | T13αH | -- | taxane 13α-hydroxylase | P450 | [^19^](https://paperpile.com/c/CAQ5G4/iBtLH) |
| 8 | T9αH-750C | -- | taxane 9α-hydroxylase-750C | P450 | This work |
|  | T9αH-725A | -- | taxane 9α-hydroxylase-725A | P450 | [^20–22^](https://paperpile.com/c/CAQ5G4/lO8He+q4Pth+iWLLP) |
| 9 | T2αH | -- | taxoid 2α-hydroxylase | P450 | [^23^](https://paperpile.com/c/CAQ5G4/15i1K) |
| 10 | TBT | -- | taxane 2α-*O*-benzoyltransferase | BAHD acyltransferase | [^24^](https://paperpile.com/c/CAQ5G4/mAMPZ) |
| 11 | T7βΗ | -- | taxoid 7β-hydroxylase | P450 | [^25^](https://paperpile.com/c/CAQ5G4/bXLlt) |
| 12 | T7AT | -- | taxane 7β-*Ο*-acetyltransferase | BAHD acyltransferase | This work  [^21^](https://paperpile.com/c/CAQ5G4/q4Pth) |
| 13 | TOT | -- | taxane oxetanase | P450 | [^20,21,26^](https://paperpile.com/c/CAQ5G4/lO8He+q4Pth+ayjUj) |
| 14 | T1βΗ-184 | 2-ODD184 | taxane 1β-hydroxylase-184 | 2-ODD | This work |
|  | T1βH-686 | 2-ODD686 | taxane 1β-hydroxylase-686 | 2-ODD | This work |
| 15 | T9dA | DeAc898 | taxane 9α-*O*-deacetylase | Alpha/beta hydrolase | This work |
| 16 | T9ox | -- | taxane C-9-oxidase | 2-ODD | This work  [^22^](https://paperpile.com/c/CAQ5G4/iWLLP) |
| 17 | T7dA | DeAc1023 | taxane 7β-*O*-deacetylase | Alpha/beta hydrolase | This work |
| 18 | PAM | -- | phenylalanine aminomutase | ammonia-lyases | [^27^](https://paperpile.com/c/CAQ5G4/02l0J) |
| 19 | PCL | AAE-867.5 | phenylalanine-CoA ligase | AMP binding / acyl-activating | This work |
| 20 | BAPT | -- | baccatin III:3-amino-3-phenylpropanoyl transferase | BAHD acyltransferase | [^28^](https://paperpile.com/c/CAQ5G4/801p0) |
| 21 | T2’αH | -- | taxane 2’α-hydroxylase | P450 | [^29^](https://paperpile.com/c/CAQ5G4/ntays)* |
| 22 | DBTNBT | -- | 3′-*N*-debenzoyl-2′-deoxypaclitaxel-*N*-benzoyl transferase | BAHD acyltransferase | [^30^](https://paperpile.com/c/CAQ5G4/AU87M) |

P450: cytochrome P450; 2-ODD: 2-oxoglutarate-dependent dioxygenase; NTF2: nuclear transport factor 2

*Enzymatic activity not observed with our reconstituted pathway.

**Table S2.** Panel of elicitation conditions

| **condition #** | **class** | **perturbation** | **concentration** |
| --- | --- | --- | --- |
| 1 | vehicle | MS media | - |
| 2 | biotic stress hormone | methyl jasmonate | 10 μM |
| 3 | biotic stress hormone | methyl jasmonate | 100 μM |
| 4 | biotic stress hormone | methyl jasmonate | 1 mM |
| 5 | biotic stress hormone | methyl jasmonate | 10 mM |
| 8 | biotic stress hormone | N-hydroxypipecolic acid | 2 mM |
| 9 | abiotic stress hormone | abscisic acid | 1 mM |
| 10 | biotic stress hormone | salicylic acid | 2 mM |
| 12 | growth hormone | trans-zeatin | 10 μM |
| 11 | PAMP (pathogen associated molecular pattern) | chitosan | 1% |
| 6 | PAMP | Flg22 (Stanford PAN facility) | 1 μM |
| 7 | PAMP | Flg22 | 10 μM |
| 13 | taxane | paclitaxel | 5 μM |
| 14 | taxane | baccatin III (MedChemExpress) | 100 μM |
| 15 | microbial challenge | Aspergillus ssp.  (**Supplemental Note 5**) | OD600 0.1 |
| 16 | microbial challenge | Aspergillus ssp. | OD600 0.1 |
|  |  | methyl jasmonate | 100 μM |
| 17 | microbial challenge | *Agrobacterium tumefaciens* GV3101 | OD600 0.01 |

**Table S3**. ^13^C and ^1^H δ assignments as well as 2D-NMR correlations of 4α,20-epoxy-taxadien-5α-ol (**2’d**) recorded in CDCl_3_.

| **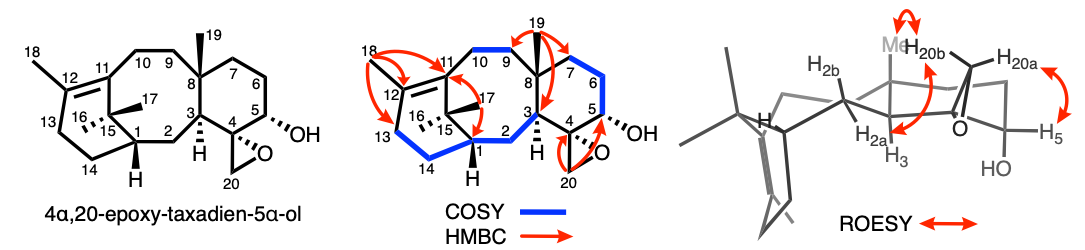** | | | | | |
| --- | --- | --- | --- | --- | --- |
| **C-#** | **δ ^13^C (ppm)** | **δ ^1^H**  **(mult.; *J* in Hz)** | **COSY** | **HMBC** | **key ROESY** |
| 1 | 43.2 | 1.67 (overlapping) | 2a, 14b | -- | -- |
| 2a | 23.2 | 0.80 (m) | 1, 2b | -- | 1, 2b, 20b |
| 2b | 23.2 | 1.29 (overlapping) | 2a, 3 | -- | 2a |
| 3 | 31.0 | 3.12 (d; 6.3) | 2b | -- | 2b, 7a |
| 4 | 62.7 | -- | -- | -- | -- |
| 5 | 73.3 | 3.33 (t; 2.6) | 6 | -- | 6, 20a |
| 6 | 27.7 | 1.82 (overlapping) | 5, 7a, 7b | -- | -- |
| 7a | 32.0 | 2.22 (dt; 7.0, 12.0) | 6, 7a | -- | -- |
| 7b | 32.0 | 1.03 (overlapping) | 6, 7b | -- | -- |
| 8 | ~40 (overlapping) | -- | -- | -- | -- |
| 9a | 30.2 | 1.93 (m) | 9b, 10a | -- | -- |
| 9b | 30.2 | 2.30 (m) | 9a, 10a, 10b | -- | -- |
| 10a | 22.1 | 1.49 (overlapping) | 9a, 9b, 10b | -- | -- |
| 10b | 22.1 | 2.00 (m) | 9b, 10a | -- | -- |
| 11 | 136.3 | -- | -- | -- | -- |
| 12 | 131.2 | -- | -- | -- | -- |
| 13a | 29.9 | 2.08 (overlapping) | 13b, 14b | -- | -- |
| 13b | 29.9 | 2.84 (overlapping) | 13a, 14a, 14b | -- | -- |
| 14a | 40.0 | 1.19 (m) | 13b, 14b | -- | -- |
| 14b | 40.0 | 2.00 (m) | 1, 13a, 13b, 14a | -- | -- |
| 15 | 39.3 | -- | -- | -- | -- |
| 16 | 30.9 | 1.03 (s) | -- | 1, 11, 15, 17 | -- |
| 17 | 25.3 | 1.32 (s) | -- | 1, 11, 15, 16 | -- |
| 18 | 21.3 | 1.83 (s) | -- | 11, 12, 13 | -- |
| 19 | 22.3 | 0.73 (s) | -- | 3, 7, 8, 9 | 2b, 6, 10b, 20b |
| 20a | 49.5 | 2.56 (d; 4.2) | 20b | 5 | 5 |
| 20b | 49.6 | 2.65 (d; 4.2) | 20a | 4, 5 | 2a, 19 |

NMR spectra are shown in **Fig. S48-53**.

s = singlet, d = doublet, dd = doublet of doublets, dt = doublet of triplets, t = triplet, q = quartet, quint = quintet, m = multiplet

**Table S4**. ^13^C and ^1^H δ assignments of taxusin (**6**) and 13β-taxusin (**6’**) recorded in CDCl_3_.

|  | **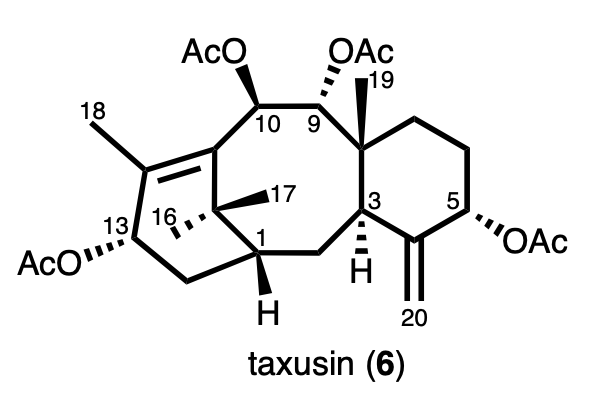** | | **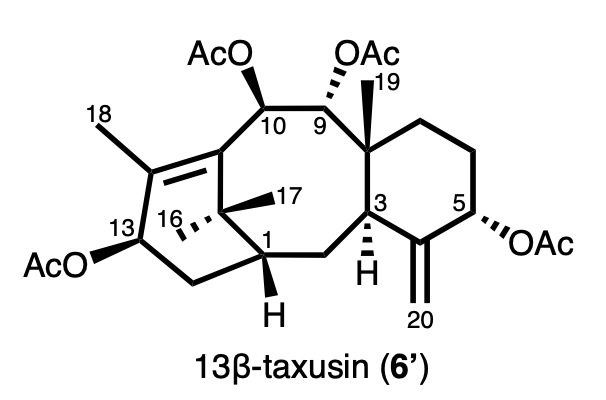** | |
| --- | --- | --- | --- | --- |
| **C-#** | **δ ^13^C (ppm)** | **δ ^1^H**  **(mult.; *J* in Hz)** | **δ ^13^C (ppm)** | **δ ^1^H**  **(mult.; *J* in Hz)** |
| 1 | 40.3 | 1.84 (m) | ~38 (overlapping) | 2.00 (m) |
| 2a | 27.3 | 1.79 (m) | N.A. | 1.73 (m) |
| 2b | 27.3 | 1.69 (m) | N.A. | 1.84 (m) |
| 3 | 37.9 | 3.00 (d; 6.5) | 38.7 | 2.80 (m) |
| 4 | 148.3 | -- | N.A. | -- |
| 5 | 76.3 | 5.36 (t; 2.5) | 76.0 | 5.38 (br, s) |
| 6a | 27.3 | 1.84 (m) | N.A. | 1.84 (m) |
| 6b | 27.3 | 1.68 (m) | N.A. | 1.69 (m) |
| 7 | 27.3 | 1.76 (m) | 27.2 | 1.74 (m) |
| 8 | 42.8 | -- | 43.4 | -- |
| 9 | 70.8 | 5.88 (d; 10.7) | 76.9 | 5.83 (d; 10.5) |
| 10 | 72.5 | 6.08 (d; 10.7) | 73.0 | 6.03 (d; 10.5) |
| 11 | 134.3 | -- | 140.8 | -- |
| 12 | 136.5 | -- | 136.3 | -- |
| 13 | 77.5 | 5.87 (m) | 72.7 | 5.33 (dd; 4.3, 9.6) |
| 14a | 31.9 | 1.06 (dd; 7.5, 14.5) | N.A. | 1.94 (dd; 9.6, 15.4) |
| 14b | 31.9 | 2.69 (dt; 14.6, 9.8) | N.A. | 2.10 (m) |
| 15 | 39.2 | -- | 47.8 | -- |
| 16 | 30.6 | 1.11 (s) | 35.6 | 1.25 (s) |
| 17 | 26.7 | 1.62 (s) | 26.1 | 1.55 (s) |
| 18 | 14.9 | 2.11 (s) | 18.7 | 2.05 (s) |
| 19 | 17.7 | 0.75 (s) | 17.2 | 0.74 (s) |
| 20a-(E) | 114 | 4.85 (s) | 113.4 | 4.84 (s) |
| 20b-(Z) | 114 | 5.21 (s) | 113.4 | 5.20 (s) |
| -OCOMe | 21.0 | 2.01 (s) | 21.1 | 2.03 (s) |
| -OCOMe | 20.7 | 2.05 (s) | 20.7 | 2.05 (s) |
| -OCOMe | 21.6 | 2.06 (s) | 21.5 | 2.08 (s) |
| -OCOMe | 21.7 | 2.16 (s) | 21.5 | 2.14 (s) |
| -OCOMe | 169.4 | -- | 169.9 | -- |
| -OCOMe | 169.4 | -- | 170.2 | -- |
| -OCOMe | 169.9 | -- | 170.4 | -- |
| -OCOMe | 169.9 | -- | 171.1 | -- |

NMR spectra are shown in **Fig. S23-28** (taxusin) and **Fig. S29-34** (13β-taxusin).

N.A. = not assigned (due to weak signal intensity or overlapping signals).

s = singlet, d = doublet, dd = doublet of doublets, dt = doublet of triplets, t = triplet, q = quartet, quint = quintet, m = multiplet

**Table S5**. ^13^C and ^1^H δ assignments as well as 2D-NMR correlations of 1β-hydroxytaxusin (**6-O1**) recorded in CDCl_3_.

| **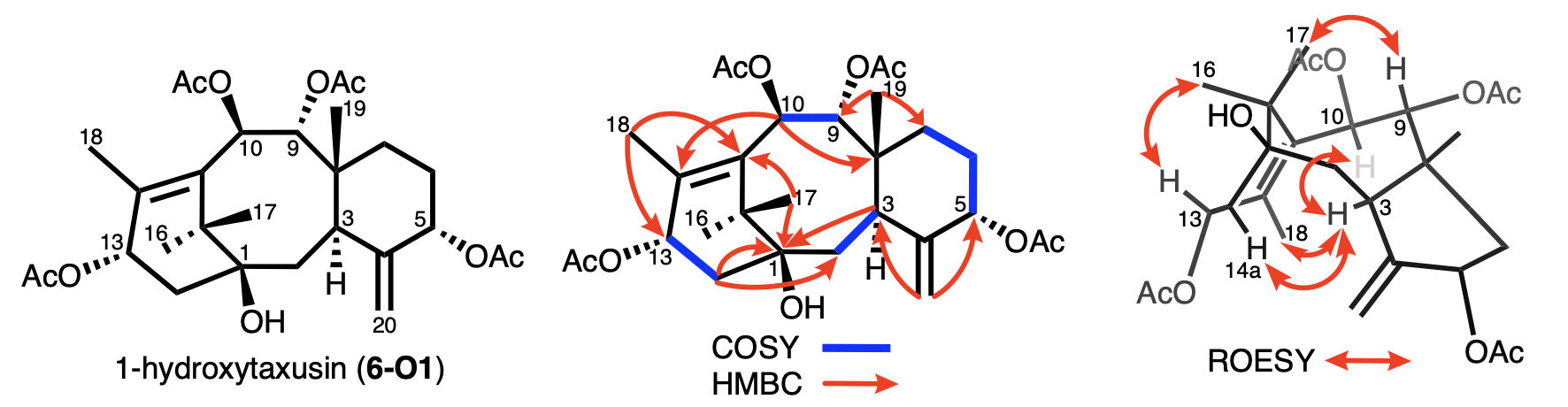** | | | | | |
| --- | --- | --- | --- | --- | --- |
| **C-#** | **δ ^13^C (ppm)** | **δ ^1^H**  **(mult.; *J* in Hz)** | **COSY** | **HMBC** | **key ROESY** |
| 1 | 76.3 | -- | -- | -- | -- |
| 2a | 38.3 | 1.79 (d; 15.2) | 2b | 1, 3, 4, 8 | -- |
| 2b | 38.3 | 1.91 (dd; 6.7, 15.2) | 2a,3 | 1, 3, 8 | -- |
| 3 | 41.7 | 2.94 (d; 6.7) | 2b | 1, 2, 4, 5, 8, 19, 20 | 6a, 10, 14a, 18 |
| 4 | 148.0 | -- | -- | -- | -- |
| 5 | 76.4 | 5.38 (t; 2.6) | 6b | 3, 4, 6, 7, 20 | 6b, 7 |
| 6a | 27.4 | 1.74 (dd; 4.4, 11.0) | 7 | 19 | -- |
| 6b | 27.4 | 1.85 (m) | 7 | -- | -- |
| 7 | 27.4 | 1.70 (m) | 6a, 6b | -- | -- |
| 8 | 43.4 | -- | -- | -- | -- |
| 9 | 77.0 | 5.88 (d; 10.7) | 10 | 7, 8, 10, 19 | 2b, 17, 19 |
| 10 | 72.2 | 6.08 (d; 10.7) | 9 | 8, 9, 11, 12, 15 | 3, 6a, 18 |
| 11 | 134.7 | -- | -- | -- | -- |
| 12 | 139.1 | -- | -- | -- | -- |
| 13 | 71.1 | 6.03 (t; 8.5) | 14a, 14b, 18 | 11, 12, 14 | 14b, 16 |
| 14a | 41.6 | 1.60 (dd; 7.1, 14.8) | 13, 14b | 1, 2, 13, 15 | -- |
| 14b | 41.6 | 2.53 (dd; 9.8, 14.8) | 13, 14a | 1, 2, 12, 13 | -- |
| 15 | 43.8 | -- | -- | -- | -- |
| 16 | 27.4 | 1.2 (s) | -- | 1, 11, 15, 17 | -- |
| 17 | 22.1 | 1.62 (s) | -- | 1, 11, 15, 16 | -- |
| 18 | 14.9 | 2.10 (s) | -- | 11, 12, 13 | -- |
| 19 | 17.9 | 0.76 (s) | -- | 3, 7, 8, 9 | -- |
| 20a-(E) | 114.4 | 4.92 (s) | 20b | 3, 4, 5 | 2a, 20b |
| 20b-(Z) | 114.4 | 5.25 (s) | 20a | 3, 5 | 20a |
| -OCOMe | 20.7 | 2.05 (s) | -- | -- | -- |
| -OCOMe | 21.1 | 2.02 (s) | -- | -- | -- |
| -OCOMe | 21.7 | 2.08 (s) | -- | -- | -- |
| -OCOMe | 21.7 | 2.16 (s) | -- | -- | -- |
| -OCOMe | 169.8 | -- | -- | -- | -- |
| -OCOMe | 169.8 | -- | -- | -- | -- |
| -OCOMe | 170.2 | -- | -- | -- | -- |
| -OCOMe | 170.3 | -- | -- | -- | -- |
| C1-OH | -- | 1.42 (s) | -- | 1, 2, 14 | -- |

NMR spectra are shown in **Fig. S35-40**.

s = singlet, d = doublet, dd = doublet of doublets, dt = doublet of triplets, t = triplet, q = quartet, quint = quintet, m = multiplet

**Table S6**. ^13^C and ^1^H δ assignments as well as 2D-NMR correlations of 15-hydroxy-11(15→1)*abeo*-taxusin (**6-O2**) recorded in CDCl_3_.

| 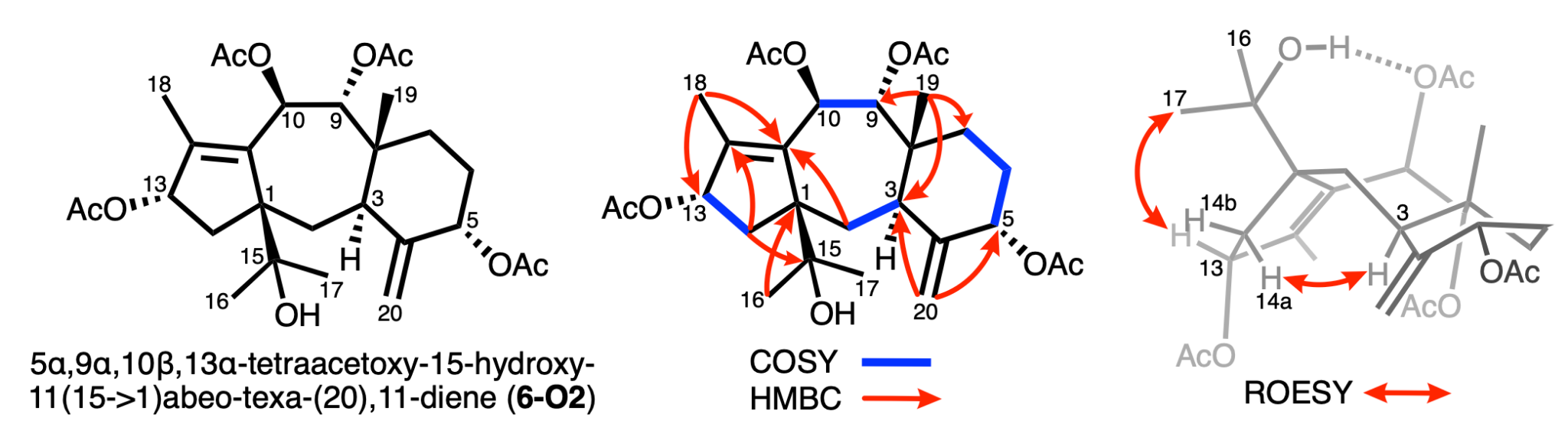 | | | | | |
| --- | --- | --- | --- | --- | --- |
| **C-#** | **δ ^13^C (ppm)** | **δ ^1^H**  **(mult.; *J* in Hz)** | **COSY** | **HMBC** | **key ROESY** |
| 1 | 62.8 | -- | -- | -- | -- |
| 2a | N.A. | 1.38 (d; 14.4) | 2b | 1, 8, 4, 11 | -- |
| 2b | N.A. | 2.17 (dd; 8.8, 14.4) | 2a, 3 | 3 | -- |
| 3 | 39.7 | 2.70 (d; 8.6) | 2b | 4, 20 | 14a |
| 4 | 146.6 | -- | -- | -- | -- |
| 5 | 74.7 | 5.31 (t; 2.7) | 6a, 6b | -- | -- |
| 6a | N.A. | 1.79 (m) | 5, 6b | -- | -- |
| 6b | N.A. | 1.88 (m) | 5, 6a | -- | -- |
| 7 | 27.4 | 1.69 (m) | 6a, 6b | -- | -- |
| 8 | 42.1 | -- | -- | -- | -- |
| 9 | 77.6 | 5.78 (d; 10.2) | 10 | -- | 2b, 19, C15-OH |
| 10 | 69.2 | 6.16 (d; 10.2) | 9 | -- | 18 |
| 11 | 137.4 | -- | -- | -- | -- |
| 12 | 144.8 | -- | -- | -- | -- |
| 13 | 79.3 | 5.53 (t; 7.5) | 14a, 14b, 18 | -- | 14b, 17 |
| 14a | 44.3 | 1.22 (dd; 7.7, 13.9) | 13, 14b | 1, 13, 15 | -- |
| 14b | 44.3 | 2.49 (dd; 7.3, 13.9) | 13, 14a | 12, 15 | -- |
| 15 | 75.2 | -- | -- | -- | -- |
| 16 | 24.6 | 1.32 (s) | -- | 1, 15, 17 | -- |
| 17 | 27.0 | 1.15 (s) | -- | 1, 15, 16 | -- |
| 18 | 11.5 | 1.83 (s) | -- | 11, 12, 13 | -- |
| 19 | 16.5 | 0.78 (s) | -- | 3, 7, 8, 9 | -- |
| 20a-(E) | 111.9 | 4.76 (s) | 20b | -- | 2a, 20b |
| 20b-(Z) | 111.9 | 5.17 (s) | 20a | 3, 5 | 20a |
| -OCOMe | 20.8 | 2.00 (s) | -- | -- | -- |
| -OCOMe | 20.8 | 2.01 (s) | -- | -- | -- |
| -OCOMe | 20.8 | 2.02 (s) | -- | -- | -- |
| -OCOMe | 21.3 | 2.04 (s) | -- | -- | -- |
| -OCOMe | 168.4 | -- | -- | -- | -- |
| -OCOMe | 169.4 | -- | -- | -- | -- |
| -OCOMe | 170.9 | -- | -- | -- | -- |
| -OCOMe | 170.9 | -- | -- | -- | -- |
| C15-OH | -- | 2.42 (s) | -- | -- | 9, 17 |

NMR spectra are shown in **Fig. S41-47**.

N.A. = not assigned (due to weak signal intensity or overlapping signals).

s = singlet, d = doublet, dd = doublet of doublets, dt = doublet of triplets, t = triplet, q = quartet, quint = quintet, m = multiplet

This assignment is consistent with previously reported assignment of the same compound in CD_3_OD.[^31^](https://paperpile.com/c/CAQ5G4/2u727)

**Table S7**. Comparison of the ^1^H δ assignments of taxusin (**6**), 1β-hydroxytaxusin (**6-O1**), and 15-hydroxy-11(15→1)*abeo*-taxusin (**6-O2**).

|  | **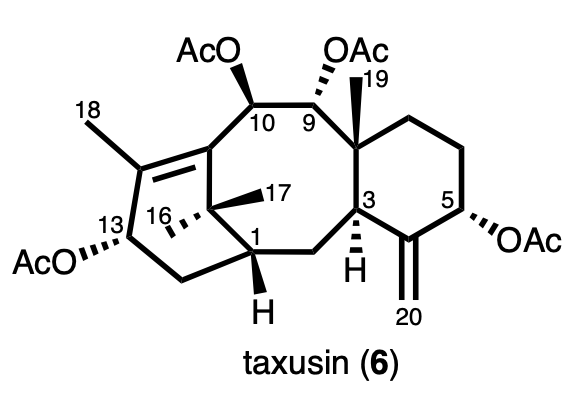** | **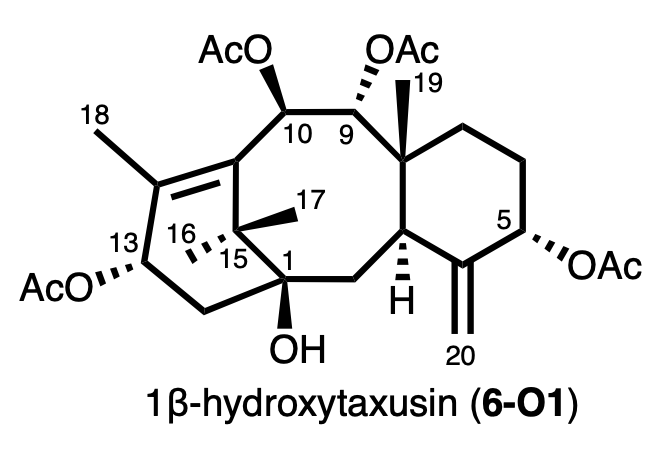** | **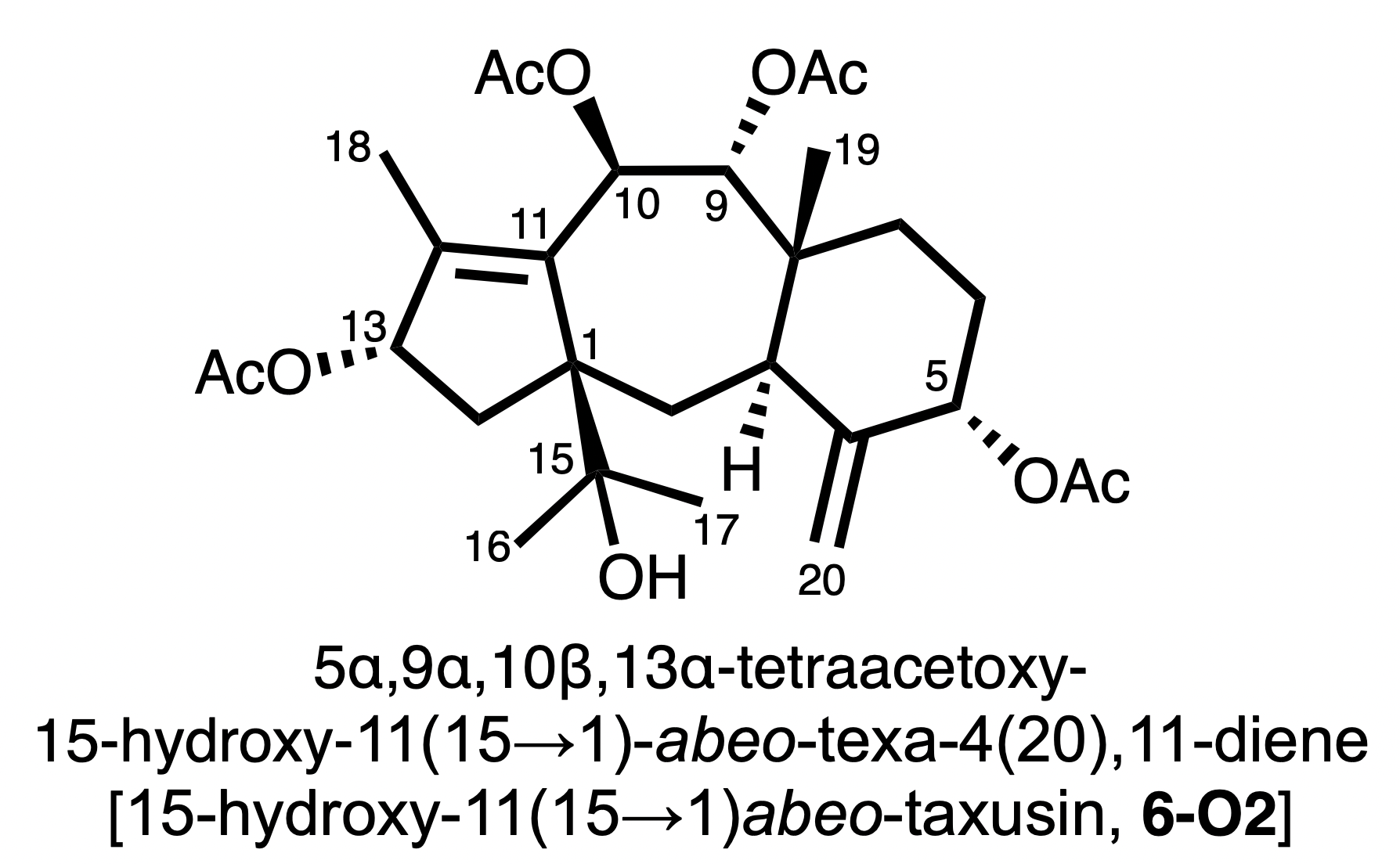** |
| --- | --- | --- | --- |
| **C-#** | **δ ^1^H (mult.; *J* in Hz)** | | |
| 1 | 1.84 (m) | -- | -- |
| 2a | 1.79 (m) | 1.79 (d; 15.2) | 1.38 (d; 14.4) |
| 2b | 1.69 (m) | 1.91 (dd; 6.7, 15.2) | 2.17 (dd; 8.8, 14.4) |
| 3 | 3.00 (d; 6.5) | 2.94 (d; 6.7) | 2.70 (d; 8.6) |
| 4 | -- | -- | -- |
| 5 | 5.36 (t; 2.5) | 5.38 (t; 2.6) | 5.31 (t; 2.7) |
| 6a | 1.84 (m) | 1.74 (dd; 4.4, 11.0) | 1.79 (m) |
| 6b | 1.68 (m) | 1.85 (m) | 1.88 (m) |
| 7 | 1.76 (m) | 1.70 (m) | 1.69 (m) |
| 8 | -- | -- | -- |
| 9 | 5.88 (d; 10.7) | 5.88 (d; 10.7) | 5.78 (d; 10.2) |
| 10 | 6.08 (d; 10.7) | 6.08 (d; 10.7) | 6.16 (d; 10.2) |
| 11 | -- | -- | -- |
| 12 | -- | -- | -- |
| 13 | 5.87 (m) | 6.03 (t; 8.5) | 5.53 (t; 7.5) |
| 14a | 1.06 (dd; 7.5, 14.5) | 1.60 (dd; 7.1, 14.8) | 1.22 (dd; 7.7, 13.9) |
| 14b | 2.69 (dt; 14.6, 9.8) | 2.53 (dd; 9.8, 14.8) | 2.49 (dd; 7.3, 13.9) |
| 15 | -- | -- | -- |
| 16 | 1.11 (s) | 1.2 (s) | 1.32 (s) |
| 17 | 1.62 (s) | 1.62 (s) | 1.15 (s) |
| 18 | 2.11 (s) | 2.10 (s) | 1.83 (s) |
| 19 | 0.75 (s) | 0.76 (s) | 0.78 (s) |
| 20a-(E) | 4.85 (s) | 4.92 (s) | 4.76 (s) |
| 20b-(Z) | 5.21 (s) | 5.25 (s) | 5.17 (s) |
| -OCOMe | 2.01 (s) | 2.05 (s) | 2.00 (s) |
| -OCOMe | 2.05 (s) | 2.02 (s) | 2.01 (s) |
| -OCOMe | 2.06 (s) | 2.08 (s) | 2.02 (s) |
| -OCOMe | 2.16 (s) | 2.16 (s) | 2.04 (s) |
| -OH | -- | 1.42 (s) | 2.42 (s) |

**Table S8**. ^13^C and ^1^H δ assignments as well as 2D-NMR correlations of 4α,20-epoxy-5α-hydroxy-taxadien-13-one recorded in CDCl_3_.

| **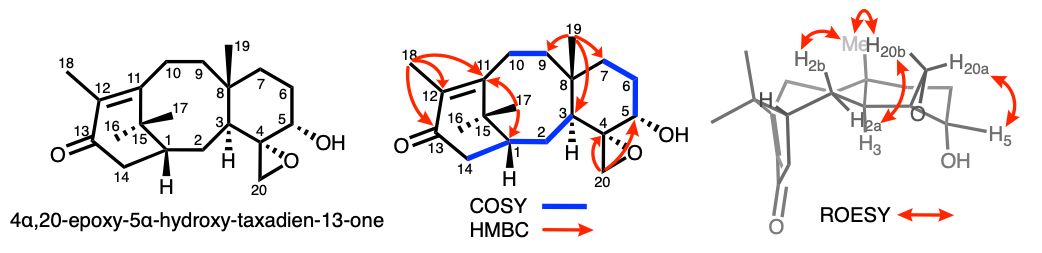** | | | | | |
| --- | --- | --- | --- | --- | --- |
| **C-#** | **δ ^13^C (ppm)** | **δ ^1^H**  **(mult.; *J* in Hz)** | **COSY** | **HMBC** | **key ROESY** |
| 1 | 40.5 | 2.08 (m) | 2a, 14b | -- | -- |
| 2a | 21.6 | 0.97 (dd; 5.0, 15.0) | 1, 2b | -- | 1, 2b, 20b |
| 2b | 21.6 | 1.38 (m) | 2a | -- | 2a, 19 |
| 3 | 31.0 | 3.15 (d; 6.3) | 2b | -- | 2b, 7a |
| 4 | 61.9 | -- | -- | -- | -- |
| 5 | 72.6 | 3.29 (t; 2.8) | 6 | -- | 6, 20a |
| 6 | 27.4 | 1.81 (m) | 5, 7a, 7b | -- | -- |
| 7a | 31.8 | 2.16 (m) | 6, 7a | -- | -- |
| 7b | 31.8 | 1.06 (m) | 6, 7b | -- | -- |
| 8 | 40.6 | -- | -- | -- | -- |
| 9a | 38.5 | 1.37 (m) | 10a, 9a | -- | -- |
| 9b | 38.5 | 2.18 (m) | 9b | -- | 17 |
| 10a | 29.5 | 2.99 (dt; 5.2, 13.6) | 10b, 9b | -- | 18 |
| 10b | 29.5 | 2.33 (m) | 10a | -- | -- |
| 11 | 161.3 | -- | -- | -- | -- |
| 12 | 133.9 | -- | -- | -- | -- |
| 13 | 200.9 | -- | -- | -- | -- |
| 14a | 39.4 | 2.22 (d; 19.4) | 14b | 1, 13, 15 | -- |
| 14b | 39.4 | 2.87 (dd; 7.2, 19.4) | 1, 14a | 13 | -- |
| 15 | 40.5 | -- | -- | -- | -- |
| 16 | 36.8 | 1.12 (s) | -- | 1, 11, 15, 17 | 1, 14b, 17 |
| 17 | 24.4 | 1.43 (s) | -- | 1, 11, 15, 16 | 1, 9b, 10b, 16 |
| 18 | 13.4 | 1.97 (d; 0.9) | -- | 11, 12, 13 | 10a |
| 19 | 22.1 | 0.78 (s) | -- | 3, 7, 8, 9 | 6, 9b |
| 20a | 49.2 | 2.57 (dd; 3.9, 0.9) | 3, 20b | 5 | 5 |
| 20b | 49.2 | 2.62 (d; 3.9) | 20a | 4, 5 | 2a, 19 |

NMR spectra are shown in **Fig. S54-59**.

s = singlet, d = doublet, dd = doublet of doublets, dt = doublet of triplets, t = triplet, q = quartet, quint = quintet, m = multiplet

**Table S9**. ^13^C and ^1^H δ assignments of 4α,20-epoxy-taxadien-5α-ol and 4α,20-epoxy-5α-hydroxy-taxadien-13-one recorded in CDCl_3_.

|  | **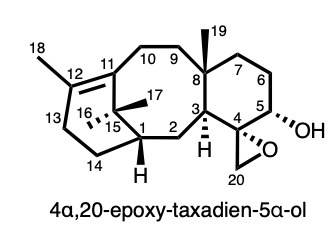** | | **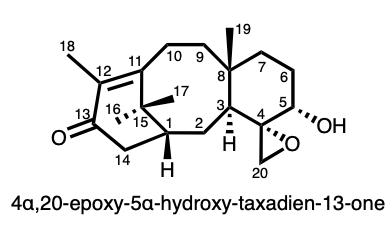** | |
| --- | --- | --- | --- | --- |
| **C-#** | **δ ^13^C (ppm)** | **δ ^1^H**  **(mult.; *J* in Hz)** | **δ ^13^C (ppm)** | **δ ^1^H**  **(mult.; *J* in Hz)** |
| 1 | 40.5 | 2.08 (m) | 43.2 | 1.67 (overlapping) |
| 2a | 21.6 | 0.97 (dd; 5.0, 15.0) | 23.2 | 0.80 (m) |
| 2b | 21.6 | 1.38 (m) | 23.2 | 1.29 (overlapping) |
| 3 | 31.0 | 3.15 (d; 6.3) | 31.0 | 3.12 (d; 6.3) |
| 4 | 61.9 | -- | 62.7 | -- |
| 5 | 72.6 | 3.29 (t; 2.8) | 73.3 | 3.33 (t; 2.6) |
| 6 | 27.4 | 1.81 (m) | 27.7 | 1.82 (overlapping) |
| 7a | 31.8 | 2.16 (m) | 32.0 | 2.22 (dt; 7.0, 12.0) |
| 7b | 31.8 | 1.06 (m) | 32.0 | 1.03 (overlapping) |
| 8 | 40.6 | -- | ~40 (overlapping) | -- |
| 9a | 38.5 | 1.37 (m) | 30.2 | 1.93 (m) |
| 9b | 38.5 | 2.18 (m) | 30.2 | 2.30 (m) |
| 10a | 29.5 | 2.99 (dt; 5.2, 13.6) | 22.1 | 1.49 (overlapping) |
| 10b | 29.5 | 2.33 (m) | 22.1 | 2.00 (m) |
| 11 | 161.3 | -- | 136.3 | -- |
| 12 | 133.9 | -- | 131.2 | -- |
| 13a | 200.9 | -- | 29.9 | 2.08 (overlapping) |
| 13b |  |  | 29.9 | 2.84 (overlapping) |
| 14a | 39.4 | 2.22 (d; 19.4) | 40.0 | 1.19 (m) |
| 14b | 39.4 | 2.87 (dd; 7.2, 19.4) | 40.0 | 2.00 (m) |
| 15 | 40.5 | -- | 39.3 | -- |
| 16 | 36.8 | 1.12 (s) | 30.9 | 1.03 (s) |
| 17 | 24.4 | 1.43 (s) | 25.3 | 1.32 (s) |
| 18 | 13.4 | 1.97 (d; 0.9) | 21.3 | 1.83 (s) |
| 19 | 22.1 | 0.78 (s) | 22.3 | 0.73 (s) |
| 20a | 49.2 | 2.57 (dd; 3.9, 0.9) | 49.5 | 2.56 (d; 4.2) |
| 20b | 49.2 | 2.62 (d; 3.9) | 49.6 | 2.65 (d; 4.2) |

s = singlet, d = doublet, dd = doublet of doublets, dt = doublet of triplets, t = triplet, q = quartet, quint = quintet, m = multiplet

**Table S10**. ^13^C and ^1^H δ assignments as well as 2D-NMR correlations of 5α,13α-diacetoxy-taxadiene recorded in CDCl_3_.

| **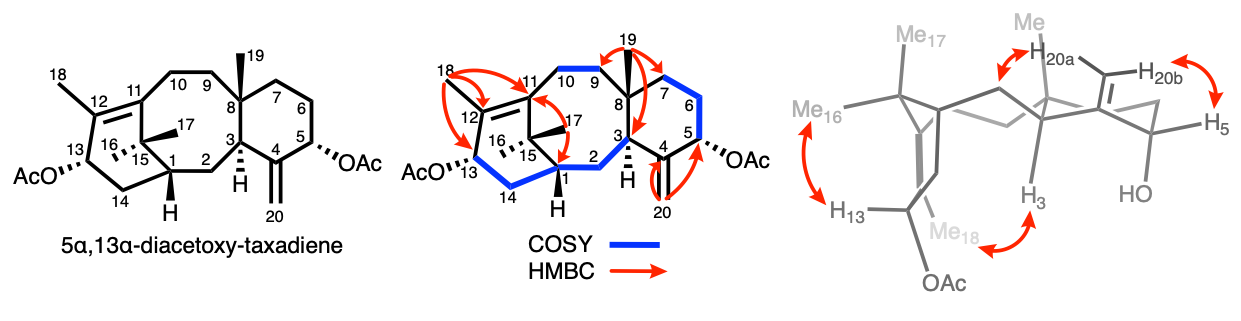** | | | | | |
| --- | --- | --- | --- | --- | --- |
| **C-#** | **δ ^13^C (ppm)** | **δ ^1^H**  **(mult.; *J* in Hz)** | **COSY** | **HMBC** | **key ROESY** |
| 1 | 40.6 | 1.81 (m) | 2a, 14a, 14b | 13 | -- |
| 2a | 28.6 | 1.60 (m) | 1, 2b | 3, 4, 8, 14 | -- |
| 2b | 28.6 | 1.65 (ddd; 2.5, 5.9, 15.6) | 2a, 3 | 3, 4, 8, 14 | -- |
| 3 | 37.5 | 3.18 (d; 5.9) | 2b | 2, 4, 7, 8, 19, 20 | 18 |
| 4 | 150.3 | -- | -- | -- | -- |
| 5 | 76.8 | 5.32 (t; 3.0) | 6 | 3, 4, 7, 20, C5-OCOMe | 6, 20b |
| 6 | 28.4 | 1.80 (m) | 5, 7a, 7b | 4, 5, 7, 8 | -- |
| 7a | 34.2 | 1.08 (m) | 6, 7b | 6, 8 | -- |
| 7b | 34.2 | 2.12 (m) | 6, 7a | 5, 19 | -- |
| 8 | 40.1 | -- | -- | -- | -- |
| 9a | 40.1 | 1.29 (m) | 9b, 10a, 10b | 3, 7, 8, 11 | -- |
| 9b | 40.1 | 2.12 (m) | 9a, 10a, 10b | 11, 19 | -- |
| 10a | 25.3 | 2.11 (m) | 9a, 9b, 10b | 8, 9 | -- |
| 10b | 25.3 | 2.83 (m) | 9a, 9b, 10a | 8, 9, 11, 12 | -- |
| 11 | 139.8 | -- | -- | -- | -- |
| 12 | 128.0 | -- | -- | -- | -- |
| 13 | 71.5 | 5.84 (tq; 7.5, 1.6) | 14a, 14b, 18 | 12, 14 | 14b, 16 |
| 14a | 32.0 | 1.09 (m) | 1, 13, 14b | 1, 2, 13, 15 | -- |
| 14b | 32.0 | 2.62 (dt; 14.4, 9.6) | 1, 13, 14a | 1, 2, 12, 13 | -- |
| 15 | 40.0 | -- | -- | -- | -- |
| 16 | 30.5 | 1.08 (s) | 17 | 1, 11, 15, 17 | -- |
| 17 | 26.8 | 1.42 (s) | 16 | 1, 11, 15, 16 | -- |
| 18 | 14.7 | 1.87 (s) | 13 | 11, 12, 13 | -- |
| 19 | 22.6 | 0.66 (s) | -- | 3, 7, 8, 9 | -- |
| 20a-(E) | 112.4 | 4.76 (s) | -- | 3, 4, 5 | 2, 20b |
| 20b-(Z) | 112.4 | 5.11 (s) | -- | 3, 4, 5 | 5, 20a |
| C5-OCOMe | 21.8 | 2.15 (s) | -- | C5-OCOMe | -- |
| C13-OCOMe | 22.0 | 2.05 (s) | -- | C13-OCOMe | -- |
| C5-OCOMe | 170.4 | -- | -- | -- | -- |
| C13-OCOMe | 171.0 | -- | -- | -- | -- |

NMR spectra are shown in **Fig. S60-65**.

s = singlet, d = doublet, dd = doublet of doublets, dt = doublet of triplets, t = triplet, tq = triplet of quartet, q = quartet, quint = quintet, m = multiplet

**Table S11**. Sequences for proteins identified in this manuscript

| **Abbreviation** | **Full name** | **Gene ID from *T. chinensis*** | **Sequences** |
| --- | --- | --- | --- |
| FoTO1 | facilitator of taxane oxidation | ctg24251_gene.1 | MAETMNEKVGADEKLIEDSRNTGQEENDYSKRLGPLSRNIIPHLINIYTCIATPRDLEIYHPDATFEDFFVRAFGIKEIKSIHYSFPKLVYDGKILEYSVEENETSPGCGELLFNIKQQYKVLYLGKEVNITTLMVLQIENGKIIKHEDRINQHPVRGRHDISVPLVGRAREGIRRLTMLMLHVRMGFGKDPTPP |
| T9αH-750C | taxane 9-alpha hydroxylase | ctg10747_gene.1 | MAFSRLIEAAAAEAPTIVTVLLLSFIFYLWRRNSSSSSTRLPPGPFQWPVIGNLHQFGRLPHLSIQHLANKYGPIMWLRLGYYPVVVVSTTEMAKEFLKIHDLAFSSRPKSGVGEHLVYNYKSMGFSPYGDYWRHIRRVWMTELMTPKRLSSFRSIREEEVCSAMRSIWEKSEQGRVAVNVSKAIDWISSSIVWRTVAGKKCSEDRDGKDLCDMVKRLMLTVKEVNGREIIPCIGWFDLQGVTRRMKETHRIFDGVAQNIIDQHINGRKREQSSDVKDIVDVLLEMADTGAINIQLDSIKAIIFDVLTGGIETATSSLEWTMTEMVRNPDIARKLQQEIESVVGKHRTVTESDLPNIEYLQCVVQESLRLHPPAPLIFPRESTEACTVGAEGYVIPPKTRLMINVWAIGRDPAVWEDSLTFKPERFMGKDMDIKGRSDFRMLPFGGGRRGCPGAQMAIGNMELILAQLMHCFDWRAEGDPSELDMSEALGTSLSRKHNLFAVPTLKLLNCI |
| T7AT | taxane 7β-*Ο*-acetyltransferase | ctg3030_gene.2 | MENPSSTDFLVKKFDPVVVAPSLPLPKTTLQLSPIDNQIGFRGFFNSLSVYNAPDDISADPVKIIREALSKVLVHYFPLAGRFRNKENGELEVDCTGEGALFVEAMVEDNISVLRDFDDLNPSFQQLVFWPPMGANIEDLHLLVVQVTRFTCGDITIGVTVCHSIFDGCGAAQFVTALADMARGEVKPLLEPIWNRELLKPEDPLHLQLYQFDSLCPPPILFEELGQASLIINSNTIKYMKQCIMEECKVFCSTFEVMAALVWVARTKAFQIPHTETVKLLFAMDMRRSFNPPFPNGYYGNAIGTAYAMDNVEDLLNGSLSRVVMIIKKSKVSLRDNYLRSNKVKDPYSLDVNKKDNNVLALSDWRRLGFHEANFGWGDPVNVTAPQLLGKGLPLLSYYLFLQPSKNQPDGIKILMSCMHPSAVKSIKMEMEAMINKFVNKS |
| T1βΗ-686 | taxane 1-beta hydroxylase 1 | ctg5594_gene.1 | MASSAQNGVELSYIDLSQFSFDSEGLKNLQNHPGVATVREACKEFGCFGVLNTGIPDDVVQKLESVSHELCAMPSEMKDRAITSNPYDSYNRIPYRESFWFPTTWDSDSVLAYFNKLWPEKDNLNLCETVMAYALGMAELQRKISFIIIASLGLDVETFYHSDFEKATSYMRVHHHYSEGKFAAGEEALFGHYDPNCFTMLYQDIGGGLQIESKEGKWVDAKPGSLVINVAESLKAWSNGRYSSAKHRVVYKDWMHRLSVGWVMQFPDKEICAPAELVDEQHPQLYRPFPYPPFLDASMKYRITIDKYAGISPIY |
| T9dA (DeAc898) | taxane C9 deacetylase | ctg19840_gene.3 | MEAATAEPRVMQDMHGFIKVYSDGSVVRAGEPYFPAAISEENNDKLGPYKDVVYNAELGLWARIYLPPPPHKKTRLPVLLFFHASGFCILSPATPVVHRLCLLWAAKTGVIIVSVKYRLAPEHRLPAAYDDSIAALQWLLAMKSTEPGAVAVDPWLHSHADLSNIFVAGESAGGNIAHYLGCWVAAQDGEIQAQVKGLILVCPFFGGEDRTPSEEGNLAVMSEADIIWKYALPVGSNRDHPFCNPVGEGRESAISSLALPPILFVIAGLDVLRDKELQYCELLKKCGKQLEVVMFDEENHGFTLFNAEDQKSLEVIRCISDFVWSKS |
| T9ox | taxane C9 oxidase | ctg4166_gene.5 | MEQNNMRNEVDLPFIDLSQFSFDSEGIKNLQNHPGVATVMESCQEWGFFRIMNTGIPNDVFQKVESVSHELFAMPQEMKDRAITSSPHDTYINHPYRESFWFPTPPHSDSVLAFCNKLWPEKDNLKLCETIGTYILSMEDLKRKISSIIIASLGLDKVETFYHSDFENGTSVFRIHHYYSDGKFAAGEEALFAHTDPHCFTILYQDNGGGLQIQSKEGNWVHVKNIPNSLIINVADSLKAWSNGRYRSVNHRVVYKDWTNRISLGWFMMFPDKEIRVPAEFIDDQHPQCYRPFTYLQFRDAFMKDRIDIDGYAGIIPTY |
| T7dA (DeAc1023) | taxane C7 deacetylase | ctg1975_gene.2 | MADNSEPRVVENLYDGVIKLYSDGSIVRGDQQSPPPPTDDYNCVPFKDIVFDHTLGLWARVYLPPQTAKTRVPVLVYYHGGGFCCEFPPSTAILDCMCHKWAATLGVIIVSAEYRLAPEHRLPAAYHDAISALHWIDSMKSGAVEVDPWFRSHADFCKVFVAGDSAGGNIANHVGIWAAGAHGDGDLQIQIKGIILGCPFFGGEERTPSGSHNSPVFNLEISDTMWRLSLPLGSNRDHPFCNPVGVGDLKEADLPPMLFVIAGQDILKDKQLQYCEFLKGCGKQVEVHVFEEEDHGFTALKMENRSAVEALRCISHFINLTN |
| PCL | phenylalanine-CoA ligase | ctg867_gene.5 | MDAEVVKSVRELGVDDVVQAGLPRHRAEIFYGQLQRAIADIGGSQTSLWHRVSKELLAPHHPHALHQLMYYSIYKNWDTSENGPPLYWFPTQESARLTNLGWMMETYGPQLLGSSYYNPITSFQSFQQFTVDHPEVYWSLVLKELSVVFHESPRCILDTSDKSRSGGVWLPGSVLNVAESCLSAKESINKTDNSIAIVWREEGRNEYPVNKMTLGELRAKVMRIANALDVVFTKGDAIAIDMPMTVNAVAIYLALILAGYVVVSIADSFVPKEIATRVRVTKAKGIFTQDFILRGGKRIPLYSRVVESGAPKAIVIPAEEELGTQLREIDVAWSKFLSFSDHLRSPEYYSAVRQPVDARTNILFSSGTSGEPKAIPWTHSPPIRCGSECWSHLDVKAGDIFCWPTNLGWVMGPVLVYSCFLSGATAAIYEGSPLDRGFGKFVQDARVTVLGTVPSMVKTWKSTGCMEGLDWSHIRTFASTGEASSIDDDLWLSSKGWYKPVIELCGGTELSACFVHGSLLQPQALGMFSTPTMTTGFVLFDDQQIPYPNDQPCIGEIGLFPRFFGSSYTLLNADHDAVYFKGMPMYKGMRLRRHGDMIERTVGGYYKAHGRSDDTMNLGGIKTSAIEIERVCNRAHEQVLETAAISISSSEGGPELLAILTVLKDGPTVSMDTLKLAFSKAIQSNLNPLFKVSFVKVISDFPRTASNKIMRRVLRDQIKQEFSLHKSRL |
| T1βH-184 | taxane 1-beta hydroxylase 2 | ctg13625_gene.1 | MASSLQNEDDLPIISLSQFSFESEGLKNLQNYPGLAKVREACKKWGFFRIVNTGIPNDVFRKMESVSHELCVMPQEMKDRAITSDPYDSYSQTPSRESFWFPTPSHSVSVQDFCNKLWPEKDNLKLCQTIGAYMFGMKELQRKISVIILASLGLDLETFYHSDFEKGTSIFRIHHHYSDGKFAVGEEALFGHTDPNCFTILYQDNGGGLQIQSKEGNWVDVKPVPNSLVINIADSLKAWSNGRYRSAKHRVVYKDWTNRISFVWMLMFPDKEIRAPTELIDEQHPQHYRPFTYHPFREATMKDHVNIDGYAGIFPTY |

**Table S12**. Experimental setup for compounds isolated in this study.

| **Compound** | **Genes** | **Plants** | **Yield** | **Column condition** | **NMR** |
| --- | --- | --- | --- | --- | --- |
| 4,20-epoxy- taxadien-5α-ol (**2’d**) | tHMGR, GGPPS, tTDS2, FoTO1, T5αH | 10 x 4-week old plants + 8 x 5-week old plants (6.14 g DW) | 280 μg  (46 μg/g DW) | 1. 25 g silica column  (EA:Hex = 2:8, 250 mL;  EA:Hex = 3:7, 50 mL; EA:Hex = 4:6, 50 mL; EA:Hex = 1:1, 40 mL; EA:Hex = 6:4, 50 mL; EA:Hex = 8:2, 50 mL;  EA 100%, 100 mL)   1. Biotage C18 6 g column   (50%, 3 CV,  50-70% 30 CV,  70-89% 5 CV) | **Table S3** |
| 4,20-epoxy-5α-hydroxy- taxadien-13-one | tHMGR, GGPPS, tTDS2, FoTO1, T13αH | 10 x 4-week old plants + 4 x 5-week old plants (5.03 g DW) | 270 μg  (54 μg/g DW) | 1. 25 g silica column  (EA:Hex = 2:8, 200 mL;  EA:Hex = 3:7, 70 mL; EA:Hex = 4:6, 50 mL; EA:Hex = 1:1, 50 mL; EA:Hex = 6:4, 50 mL; EA:Hex = 8:2, 50 mL;  EA 100%, 100 mL)   1. Biotage C18 6 g column   (40%, 5 CV,  40-60% 20 CV) | **Table S8** |
| 5α,13α-diacetoxy-taxadiene | tHMGR, GGPPS, tTDS2, FoTO1, T13αH, TAX19 | 9 x 4-week old plants + 12 x 5-week old plants (10.38 g DW) | 16.3 mg  (1.57 mg/g DW) | 1. 42 g silica column  (EA:Hex = 1:9, 250 mL;  EA:Hex = 2:8, 250 mL; EA:Hex = 4:6, 250 mL)   1. Biotage C18 6 g column   (45%, 5 CV,  45-85% 40 CV,  85-100%, 5 CV) | **Table S10** |
| taxusin (**6**) | tHMGR, GGPPS, tTDS1, tTDS2, FoTO1, T5αH, TAT, T10βH, DBAT, T13αH, T9αH-750C, TAX19, CJM236* | 12 x 5-week old plants  (10.94 g DW) | 4.1 mg (375 μg/g DW) | 1. 100 g silica column  (EA:Hex = 1:4, 2 L)  2. Biotage C18 6 g column  (45% 2 CV,  45-49% 5 CV,  49% 2 CV,  49-52% 6 CV,  52-55% 10 CV) | **Table S4** |
| 13β-taxusin (**6’**) |  |  | <1 mg  (<91 μg/g DW) |  | **Table S4** |
| 1β-hydroxytaxusin (**6-O1**) | tHMGR, GGPPS, tTDS1, tTDS2, FoTO1, T5αH, TAT, T10βH, DBAT, T13αH, T9αH-750C, TAX19, T1βH-184 | 8 x 4-week old plants +  6 x 5-week old plants  (12.25 g DW) | 2.2 mg (180 μg/g DW) | 1. 100 g silica column  (EA:Hex = 1:4, 1.5 L;  EA:Hex = 1:3, 0.5 L;  EA:Hex = 2:3, 0.5 L;  EA:Hex = 3:2, 0.5 L)  2. Biotage C18 6 g column  (40% 5 CV,  40-45% 15 CV) | **Table S5** |
| 15-hydroxy-11(15→1)*abeo*-taxusin (**6-O2**) |  |  | 0.4 mg  (33 μg/g DW) |  | **Table S6** |
| baccatin III (**16**) | HMGR, GGPPS, TDS, FoTO1, ΤΑΤ, T10βH, DBAT, T13αH, T9αΗ-CYP750C, T2αH, TBT, T7βH, T7AΤ, TOT, T1βH-686, DeAc898, DeAc1023, and T9ox (Note: exclude T5αH) | 53 x 4-week old plants  (30.70 g DW) | 1.33 mg  (estimated ~20% pure; ~8.7 μg/g DW) | 1. 100 g silica column  (EA:Hex = 1:1, 1 L;  EA:Hex = 3:2, 0.5 L;  EA:Hex = 7:3, 0.5 L;  EA:Hex = 4:1, 0.5 L;  EA:Hex = 9:1, 0.3 L;  EA, 0.5 L)  2. Biotage C18 6 g column  (30% 2 CV,  30-32% 2 CV,  32-37% 10 CV) | **Extended Data Figure 7b** |

Solvent system for biotage Sfär C18 D Duo 100 Å 30 µm column: A =water, B = acetonitrile. Percentage of solvent B is listed. DW: dry weight, EA: ethyl acetate, Hex: hexane, CV: column volume.

*CJM236 is a non-functional gene accidentally included in this expression. It does not affect the accumulation of taxusin (**6**) and 13β-taxusin (**6’**).

**Table S13**. Oligonucleotide primers used in this study.

| **Agrobacterium vector (pEAQ) cloning primers** | | |
| --- | --- | --- |
|  | FoTO1 fwd | atattctgcccaaattcgcgaccggtATGGCAGAAACAATGAATGAGAAAGTTGGCG |
|  | FoTO1 rev | taatgaaaccagagttaaaggcctcgagTCATGGAGGAGTTGGATCCTTTCCAAAGC |
|  | T9αH-750C fwd | ttctgcccaaattcgcgaccggtATGGCATTTTCTAGACTGATTGAAGCAGC |
|  | T9αH-750C rev | gagttaaaggcctcgagTTAGATGCAATTTAACAATTTCAGGGTAGGAACTG |
|  | T7AT fwd | atattctgcccaaattcgcgaccggtATGGAGAATCCAAGCTCAACAGACTTCCT |
|  | T7AT rev | agagttaaaggcctcgagTCATGATTTATTCACAAATTTGTTTATCATGGCTTCC |
|  | T1βH-184 fwd | aagcttctgtatattctgcccaaattcgcgaccggtATGGCTTCCTCGCTGCAGAATGAA |
|  | T1βH-184 rev | taatgaaaccagagttaaaggcctcgagTCAATAAGTAGGAAATATGCCTGCGTAACCAT |
|  | T1βH-686 fwd | tctgtatattctgcccaaattcgcgaccggtATGGCTTCCTCGGCGCAGAAT |
|  | T1βH-686 rev | aaaccagagttaaaggcctcgagTCAATAAATAGGGGATATGCCGGCGTATT |
|  | T9ox fwd | tattctgcccaaattcgcgaccggtATGGAACAGAATAACATGCGAAATGAAGTTGATCT |
|  | T9ox rev | atgaaaccagagttaaaggcctcgagTCAATAAGTAGGAATTATGCCTGCATAACCGTCA |
|  | T9dA fwd | agcttctgtatattctgcccaaattcgcgaccggtATGGAAGCAGCTACGGCGGAGC |
|  | T9dA rev | aaaccagagttaaaggcctcgagCTAACTTTTACTCCAGACGAAATCAGAGATGCAG |
|  | T7dA fwd | agcttctgtatattctgcccaaattcgcgaccggtATGGCGGATAACAGCGAGCCTC |
|  | T7dA rev | ccagagttaaaggcctcgagTTAATTAGTTAAGTTAATGAAGTGAGAGATGCAACGG |
| **FoTO1 mutants** | |  |
|  | C49T fwd | CCTTATCAATATATATTGTTGCATAGCAACACCAC |
|  | C49T rev | GTGGTGTTGCTATGCAACAATATATATTGATAAGG |
|  | F83H fwd | GAGATCAAGTCAATCTTTTATTCCTTTCCAAAG |
|  | F83H rev | CTTTGGAAAGGAATAAAAGATTGACTTGATCTC |
|  | A61H-N63D fwd | GACCTTGAAATATATGCTCCGAATGCGACATTTGA |
|  | A61H-N63D rev | TCAAATGTCGCATTCGGAGCATATATTTCAAGGTC |
|  | F57L fwd | GCAACACCACGTGACTTTGAAATATATCATCC |
|  | F57L rev | GGATGATATATTTCAAAGTCACGTGGTGTTGC |
|  | I114F fwd | GCGGAGAGTTACTGATTAACATCAAGCAACAG |
|  | I114F rev | CTGTTGCTTGATGTTAATCAGTAACTCTCCGC |
|  | Truncate N-term | ctgtatattctgcccaaattcgcgaccggtATGAGATTAGGACCGCTTTCACGCAATATAATTCCTC |
|  | Truncate C-term | aatgaaaccagagttaaaggcctcgagCTAGTTAATCCGGTCTTCGTGCTTGATGATCTTCCC |
| **Tagged constructs** | |  |
|  | V5 Tag - C terminus fwd | TAGAATCAAGTCCAAGAAGTGGATTTGGAATTGGCTTTCCTCCAGAGCCTGGAGGAGTTG |
|  | V5 Tag - C terminus rev | atgaaaccagagttaaaggcctcgagCTAAGTAGAATCAAGTCCAAGAAGTGGATTTGG |
|  | V5 Tag - N Terminus fwd | GCCAATTCCAAATCCACTTCTTGGACTTGATTCTACTggctctggaGCAGAAACAATGAA |
|  | V5 Tag - N Terminus rev | atattctgcccaaattcgcgaccggtatgGGAAAGCCAATTCCAAATCCACTTC |
|  | HA-T5aH fwd | GAAACCAGAGTTAAAGGCCTCGAGTTAAGCATAATCTGGAACATCATATGGATAtccagagcC |
|  | HA-T5aH rev | TTAAGCATAATCTGGAACATCATATGGATAtccagagcC TGGTCTCGGAAACAGTTTAAT |
| **E. coli expression vector (pET28a) cloning primers** | | |
|  | pET fwd | TAGctcgagcaccaccaccaccaccactgagatccgg |
|  | pET rev | atggctgccgcgcggcaccaggccgctgctgtgatgatgatgatgatggctgctgcccat |
|  | 3xFLAG-TEV fwd | ggcctggtgccgcgcggcagccatgactataaagatgatgacgacaaaGACTATAAGGAC |
|  | 3xFLAG-TEV rev | AGACTGAAAGTATAAATTCTCtttatcgtcgtcatccttgtaatcCTTATCATCGTCGT |
|  | T5αH fwd | gacgataaagagaatttatactttcagtctTCCTCCCTTAAACTTCCTCCTGGGAAATTAG |
|  | T5αH rev | tctcagtggtggtggtggtggtgctcgagCTATGGTCTCGGAAACAGTTTAATGGAAAAT |
|  | TDS2 fwd | tgacgacgataaagagaatttatactttcagtctagcggtagcccgaccaagttggcta |
|  | TDS2 rev | tggtggtggtggtggtgctcgagCTAtacttgaatcggttcaatgtaaacttttcttata |
|  | mTurq-FoTO1 fwd | acgataaaGAGAATTTATACTTTCAGTCTGTGAGTAAGGGTGAAGAATTATTTACTGGCG |
|  | mTurq-FoTO1 rev | accgctcatgcttaatttctcctctttaattCTATGGAGGAGTTGGATCCTTTCCAAAGC |
|  | mTurq-FoTO1∆Cterm rev | tcagtggtggtggtggtggtgctcgagTCAGTGTTGGTTAATCCGGTCTTCGTGC |
| **Others** | | |
| FoTO1-mTurq2 | FoTO1 rev | AAATAATTCTTCACCCTTACTCACtccagagccTGGAGGAGTTGGATCCTTTCCAAAGCC |
|  | C-term mTurq2 fwd | ggctctggaGTGAGTAAGGGTGAAGAA |
|  | C-term mTurq2 rev | ttaatgaaaccagagttaaaggcctcgagCTATTTATACAACTCATCCATTCCGAGCGTGATG |
| mTurq2-FoTO1 | N-term mTurq2 fwd | ctgtatattctgcccaaattcgcgaccggtATGGTGAGTAAGGGTGAAGAATTATTTACTGGC |
|  | N-term mTurq2 rev | tccagagccTTTATACAACTCATCCATTCCGAGCGTGATGC |
|  | FoTO1 fwd | GGAATGGATGAGTTGTATAAAggctctggaGCAGAAACAATGAATGAGAAAGTTGGCGCT |
| T5αH-mCherry | T5αH-mCherry rev | TCGCCCTTGCTCACCATGCTTCCCGAACCTGGTCTCGGAAACAGTTTAATGGAAAATCCC |
|  | T5αH-mCherry fwd | CCATTAAACTGTTTCCGAGACCAGGTTCGGGAAGCATGGTGAGCAAGGGCGAGGAGG |

# Supplementary Figures

**
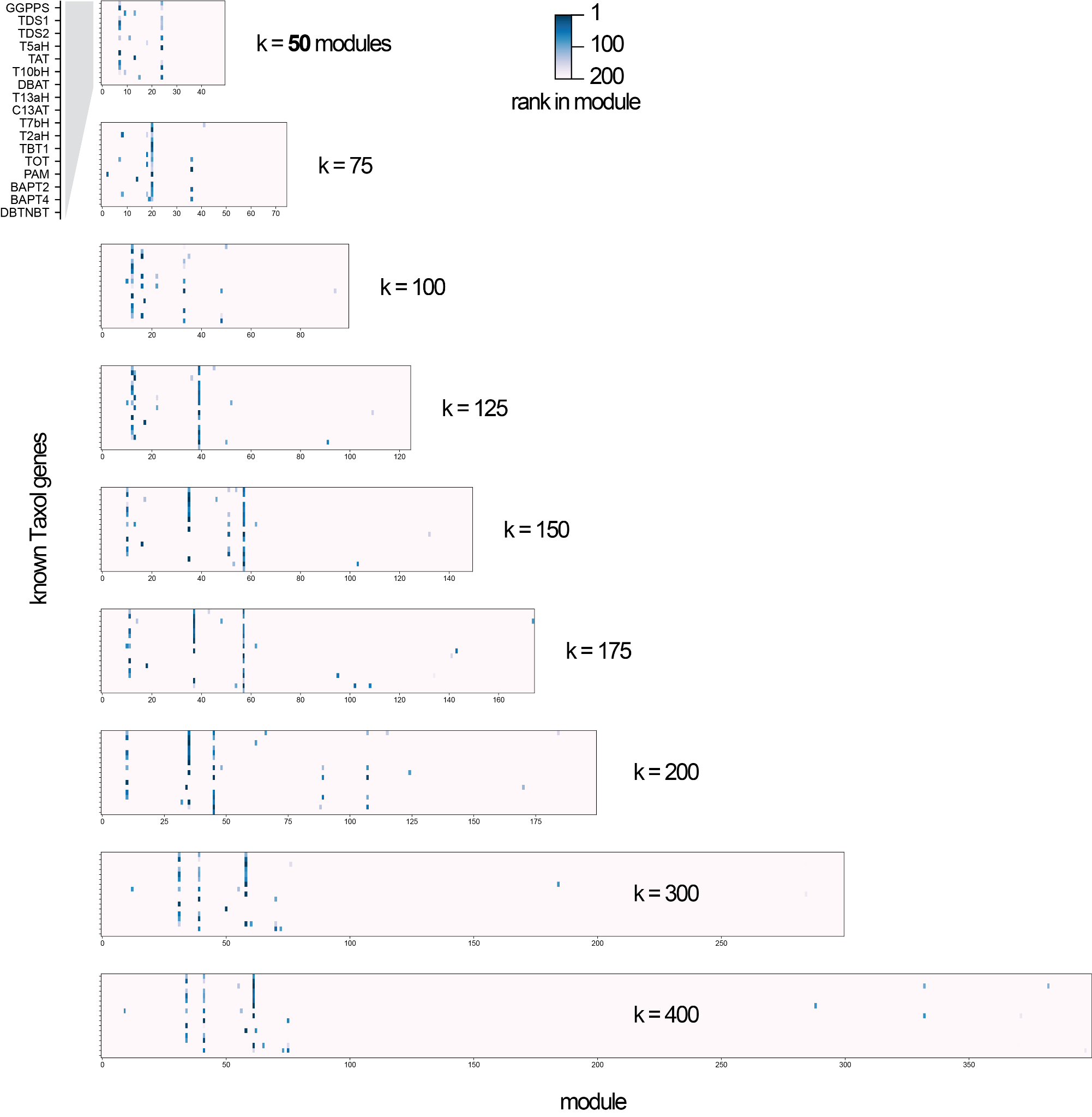
Fig. S1.** **Gene modules with different parameters for matrix factorization**. Consensus non-negative matrix factorization (cNMF) [^32^](https://paperpile.com/c/CAQ5G4/XrOuW) was run with different numbers of modules (k). The ranking of 17 genes previously shown to be associated with taxane biosynthesis (x-axis) were visualized for all modules produced by each cNMF run. When k ≥ 150, these known taxane enzymes consistently cluster into three dominant modules. In each plot, modules are ranked by by total usage in our dataset, as defined by the cNMF analysis (e.g. module #1 from each run has the highest usage, or expression, across cells in the dataset).

**
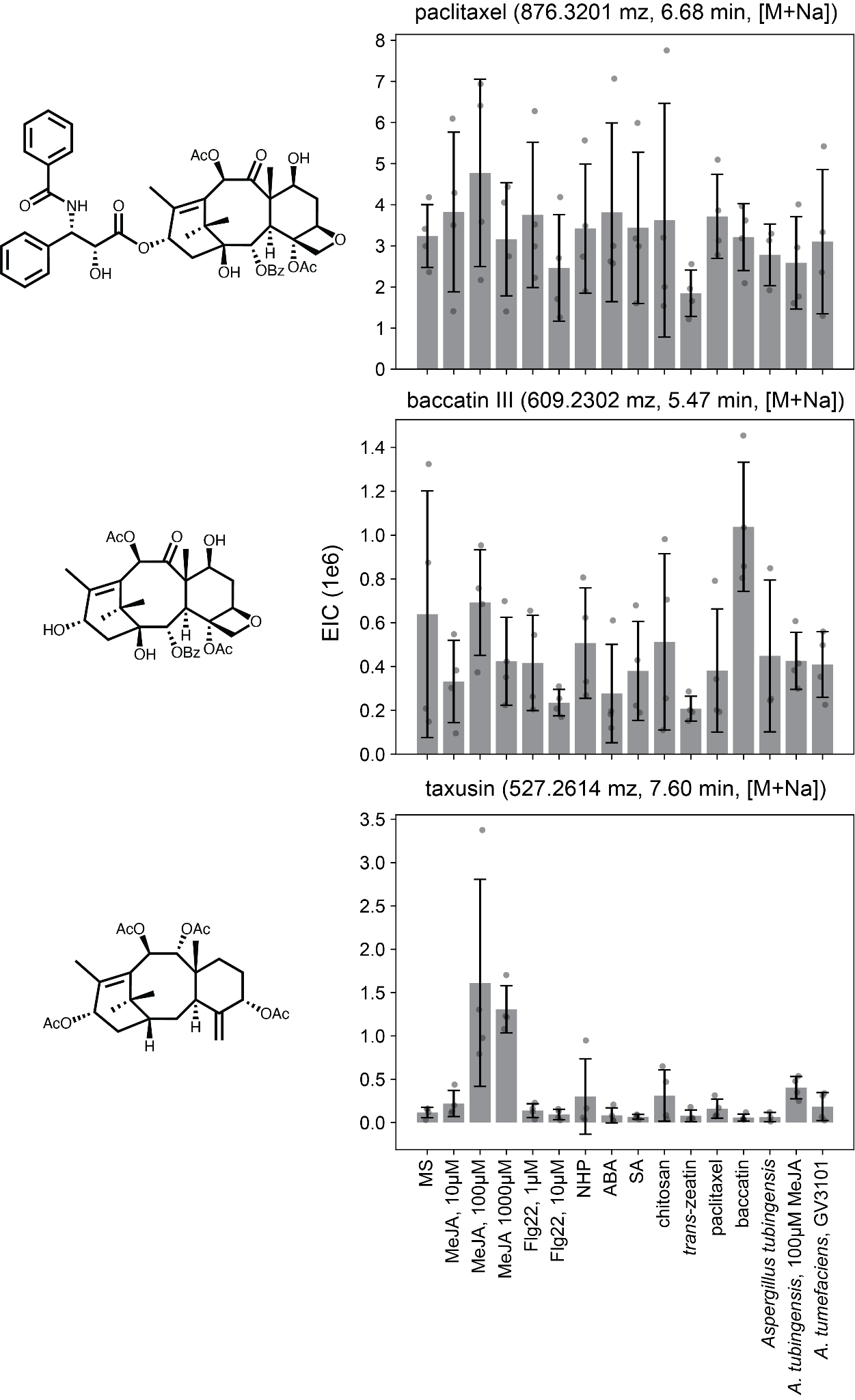
**

**Fig. S2. Accumulation of known taxanes after elicitation.** Mature *Taxus media* needles were placed in MS media containing each indicated elicitor (see **Table S2** for details) for 3 days. Subsequently, tissues were lyophylized, homogenized and extracted (15 mg DW / mL) into 75% ACN/water. Paclitaxel and baccatin III were not observed to accumulate to higher levels after 3 days of any elicitor, possibly due to initial accumulated stores of these metabolites. In contrast, chitosan and high levels of methyl jasmonate (MeJA) result in increases in taxusin accumulation, suggesting these elicitors can activate expression of early pathway enzymes.

**
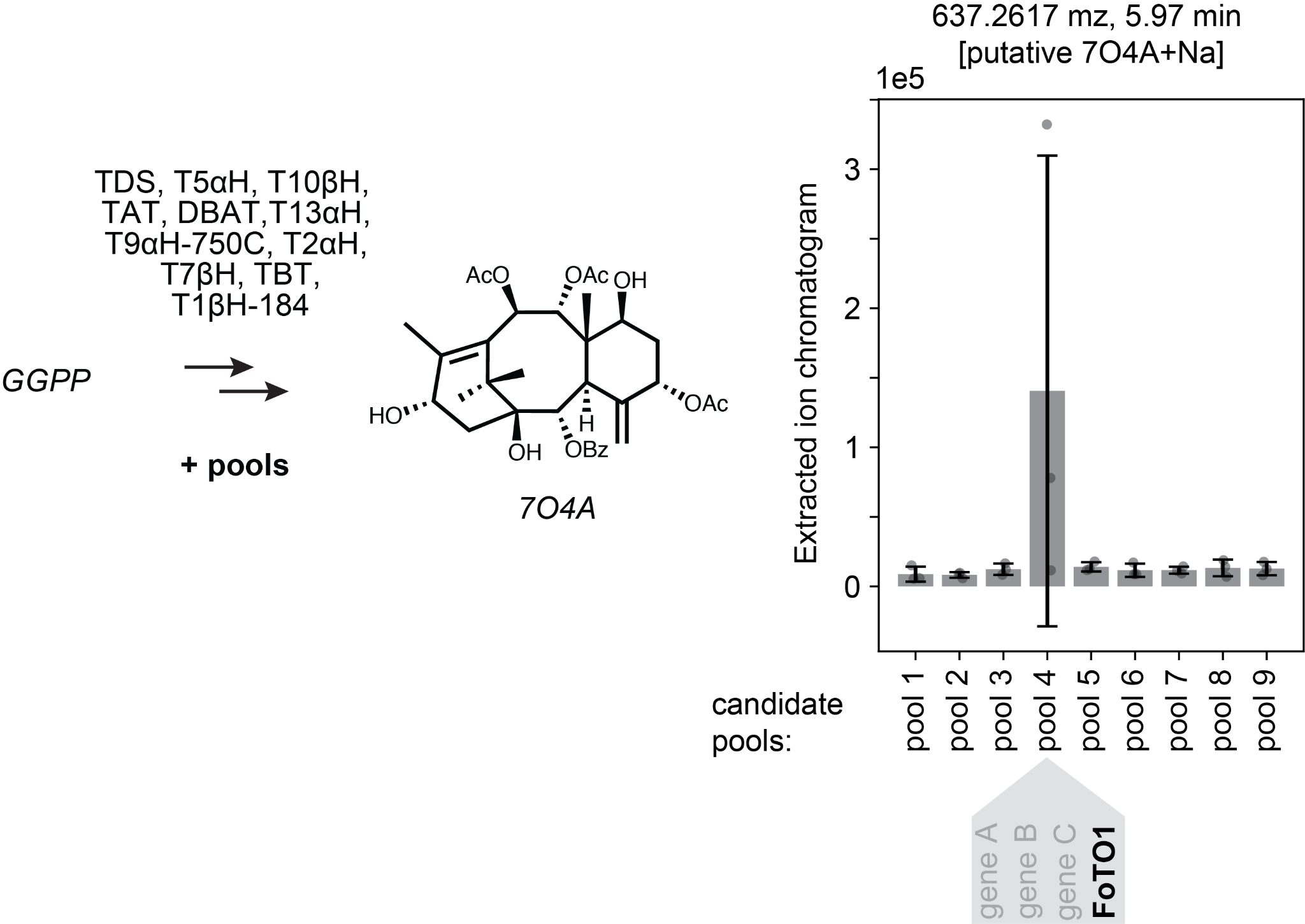
**

**Fig. S3. Representative candidate screening and identification of FoTO1.** Candidate genes were screened in pools of 4 genes by co-expression with pathway enzymes in *N. benthamiana*. FoTO1-containing pool was originally identified by an increased yield of a putative 7O4A taxane.

**
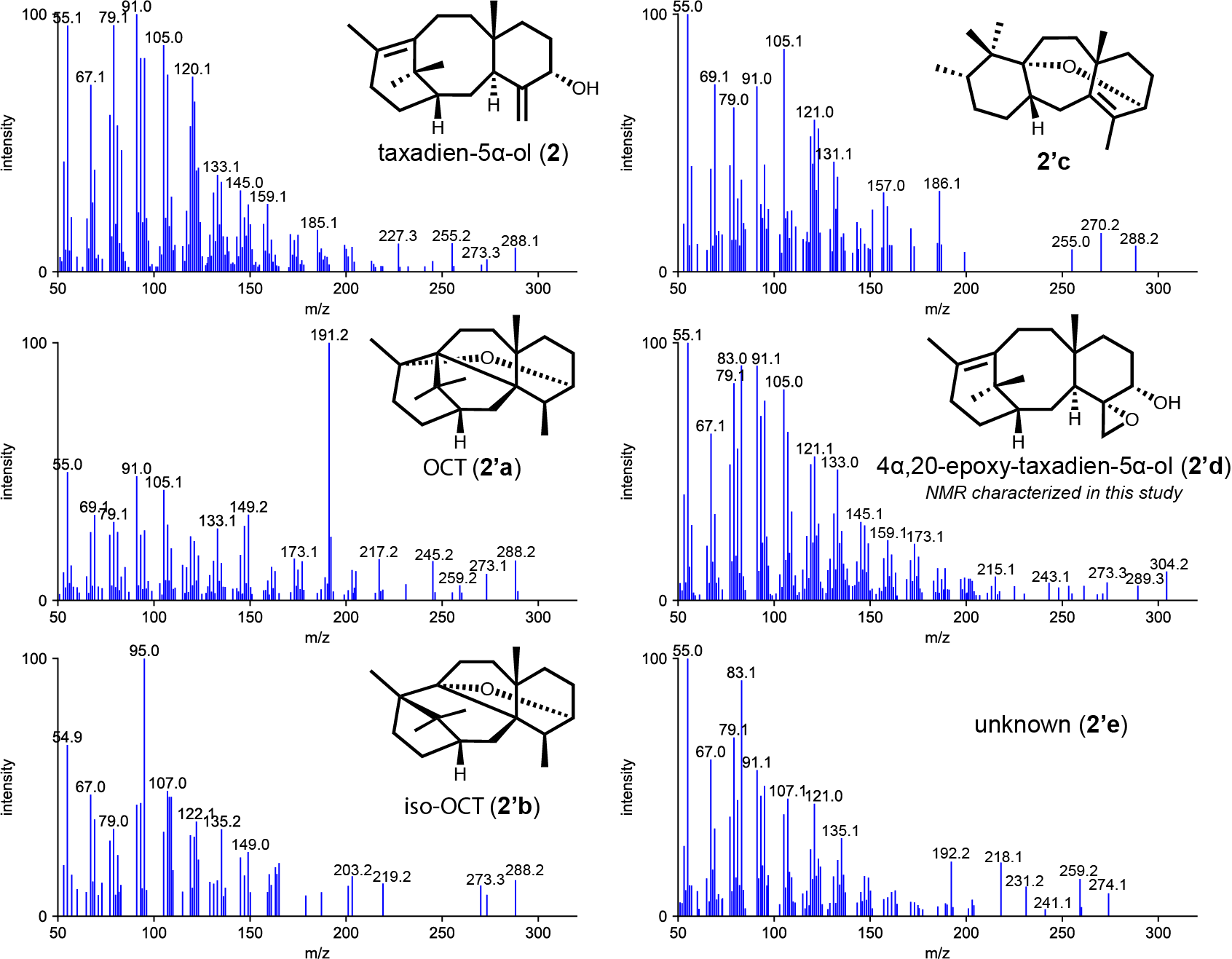
**

**Fig. S4.** **MS spectra of T5αH products**. GCMS spectra of major T5αH products and their structures are shown. Compounds **2**, **2’a-c** were previously reported[^1^](https://paperpile.com/c/CAQ5G4/vB77) while **2’d** is purified and confirmed in this study (**Table S3**). Regions between m/z 50 to 320 are shown. While we were not able to obtain sufficient amounts of **2’e** for structural characterization, the MS spectra of **2’e** reveals a fragmentation pattern consistent with taxanes, particularly in the region m/z 50–150. The observed parent ion mass (m/z 274) suggests that **2’e** could potentially be a saturated form (+2H) of taxadiene. Additional work will be required to confirm its identity and significance in FoTO1-T5αH catalysis.

**
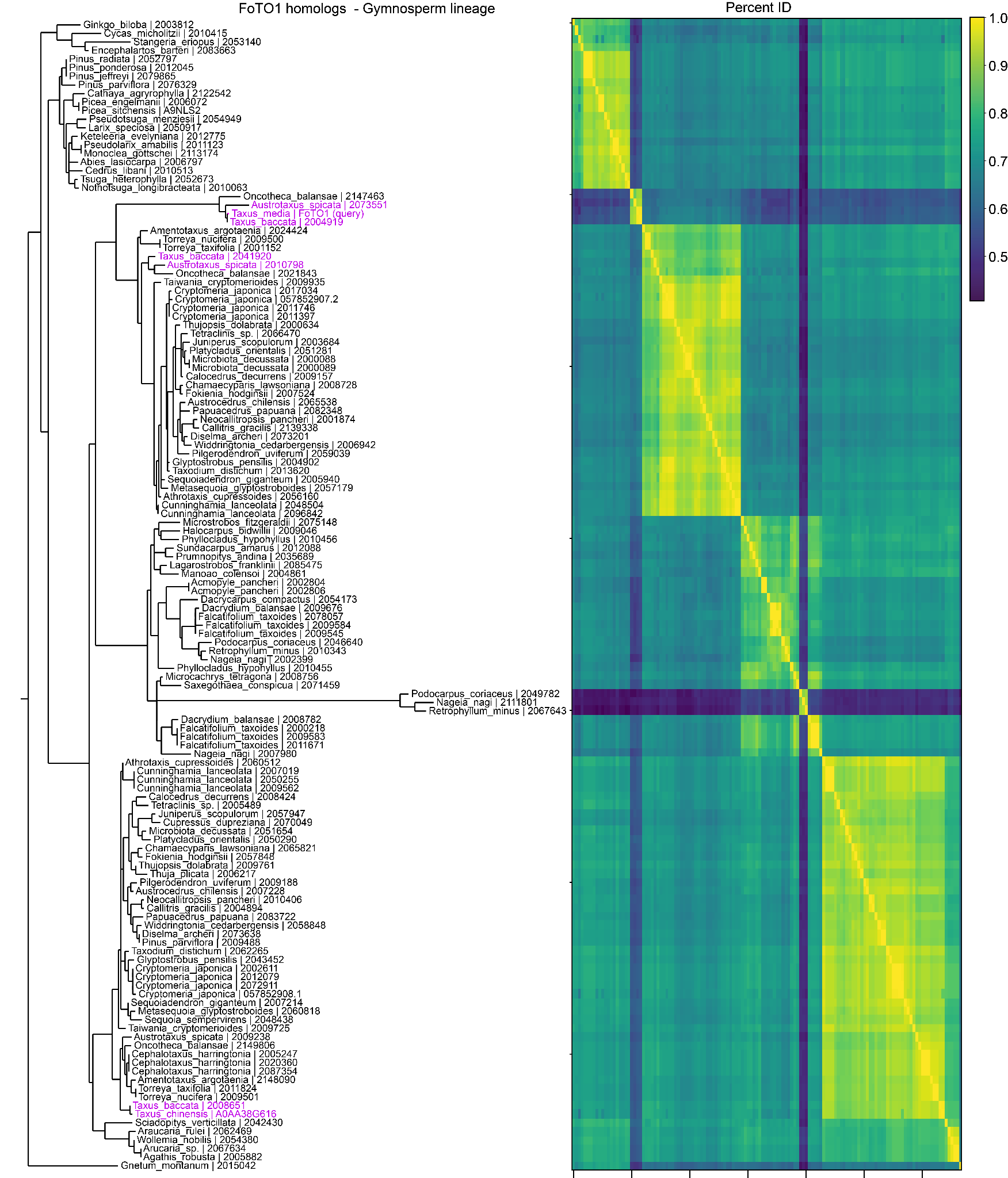
Fig. S5.** **FoTO1 homologs in gymnosperm lineage**. Homologs of FoTO1 identified by jackhmmer (see **Methods**) from Uniprot, Refseq and 1KP databases were assembled into a phylogenetic tree with FastTree[^33^](https://paperpile.com/c/CAQ5G4/lWaeo). (left) The gymnosperm lineage of this tree is displayed, with genes from the genus *Taxus* highlighted in purple. (right) A percent identity matrix of all proteins in this tree compared with each other protein.

**
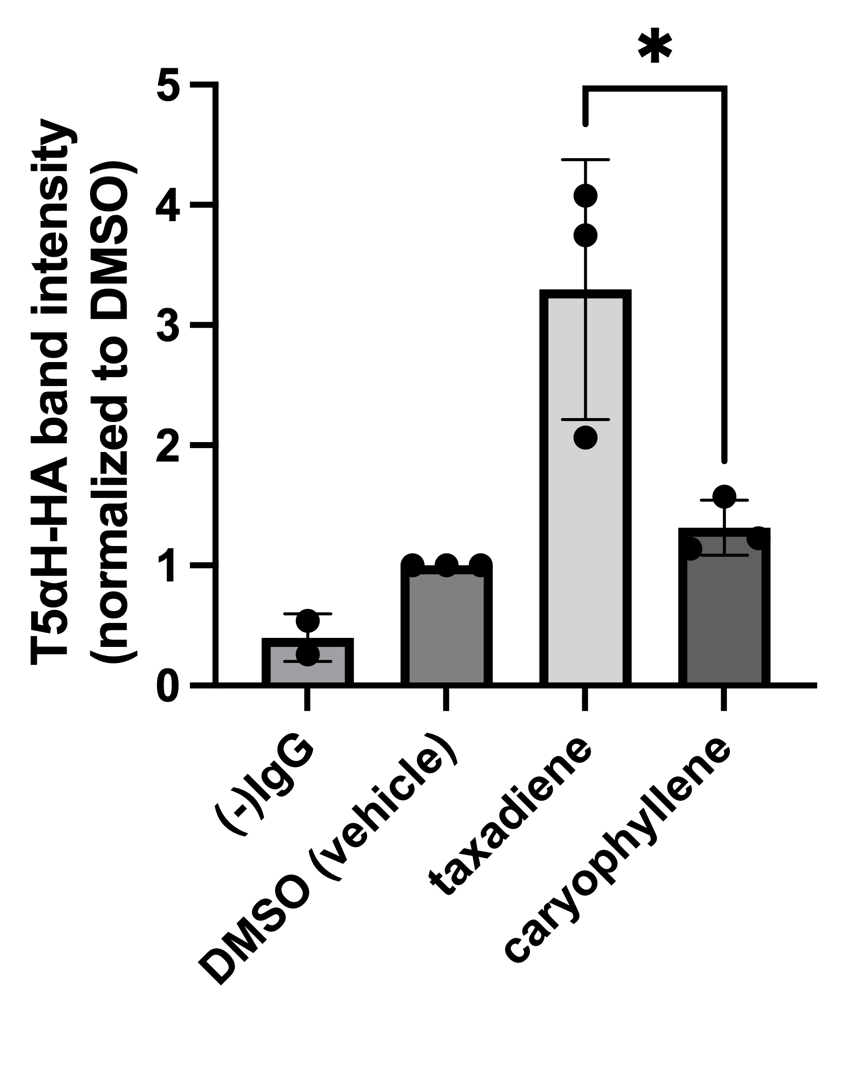
**

**Fig. S6.** **Quantification of co-immunoprecipitation T5αΗ by FoTO1**. V5-FoTO1 and T5αΗ-HA were coexpressed in *N. benthamiana* leaves for five days. Lysates were treated for 15 minutes with 45 μM taxadiene (**1**), caryophyllene or a DMSO vehicle prior to immunoprecipitation and quantification of T5αΗ-HA by western blot. Caryophyllene is a sesquiterpene with hydrophobicity similar to taxadiene. (-)IgG indicates immunoprecipitation without antibody, as a control for nonspecific binding to beads. Band intensities were normalized to the DMSO vehicle condition and p-values were calculated by two-tailed t-test. Three dots indicate independent replicates conducted on different days.

**
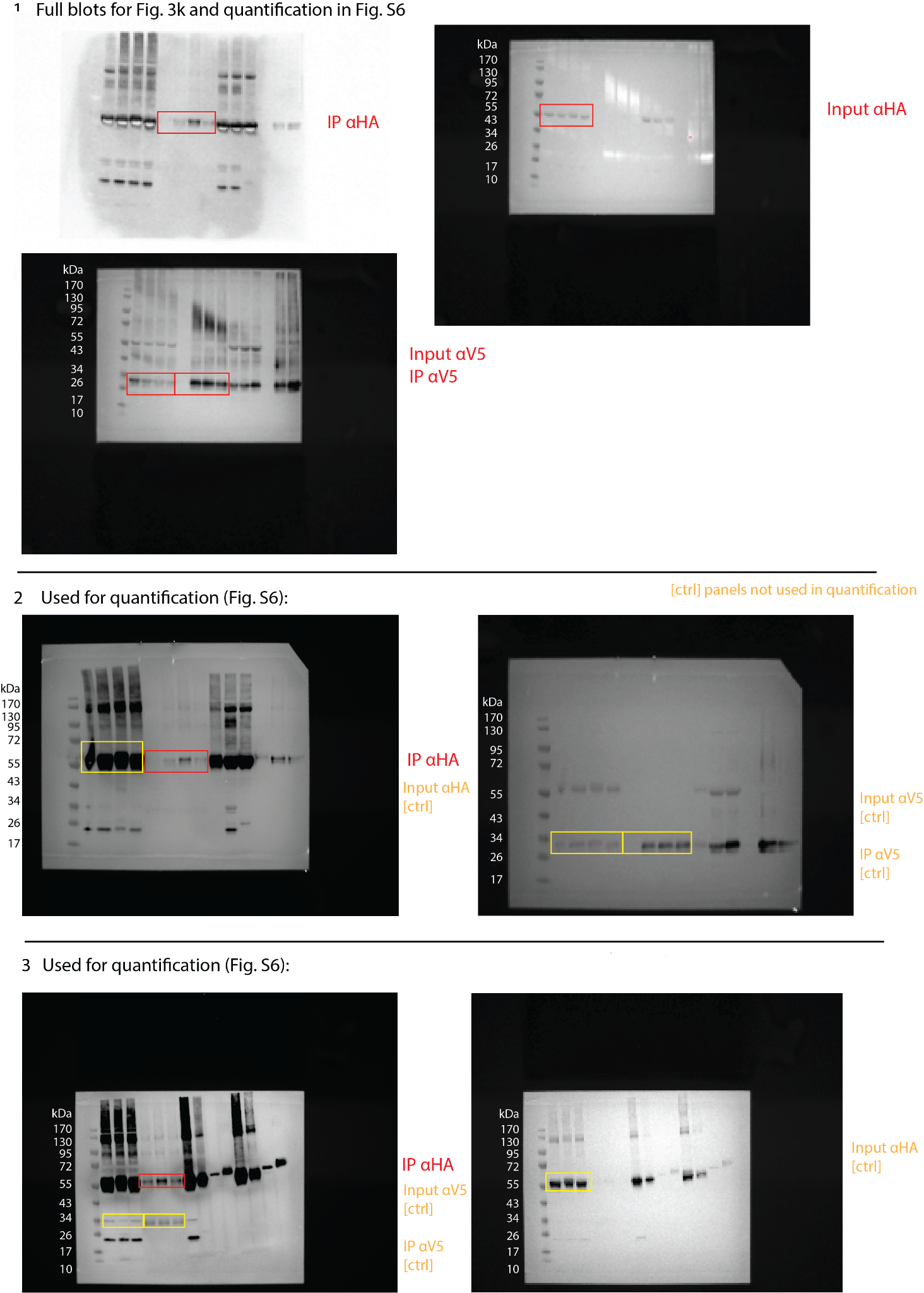
**

**Fig. S7. Raw gel image used for Fig. 3k and Fig. S6.**


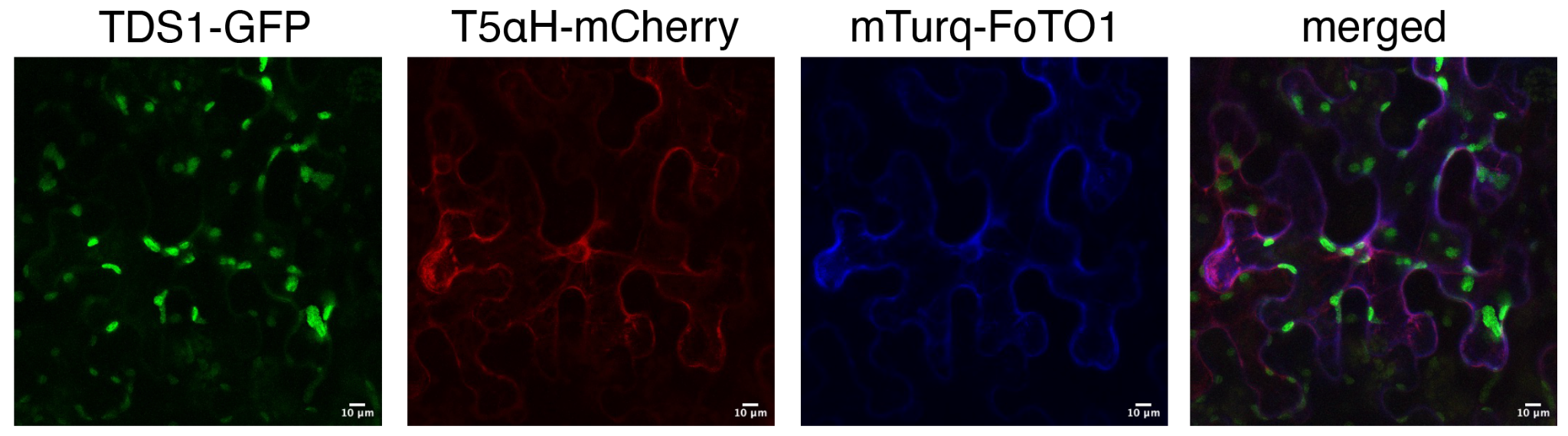


**Fig. S8.** **TDS1, T5αH, FoTO1 show different subcellular localizations in *Nicotiana benthamiana***. TDS1, T5αΗ, and FoTO1 were fused with fluorescent protein GFP, mCherry, and mTurq, respectively, and the fusion proteins were expressed in *N. benthamiana* using *Agrobacterium*-mediated expression. Leaves were harvested at 5 day post infiltration and imaged by Leica SP8 confocal microscopy. Fluorescence from GFP (green), mCherry (red), and mTurq (blue) are shown. Scale bars indicate 10 μm.

**
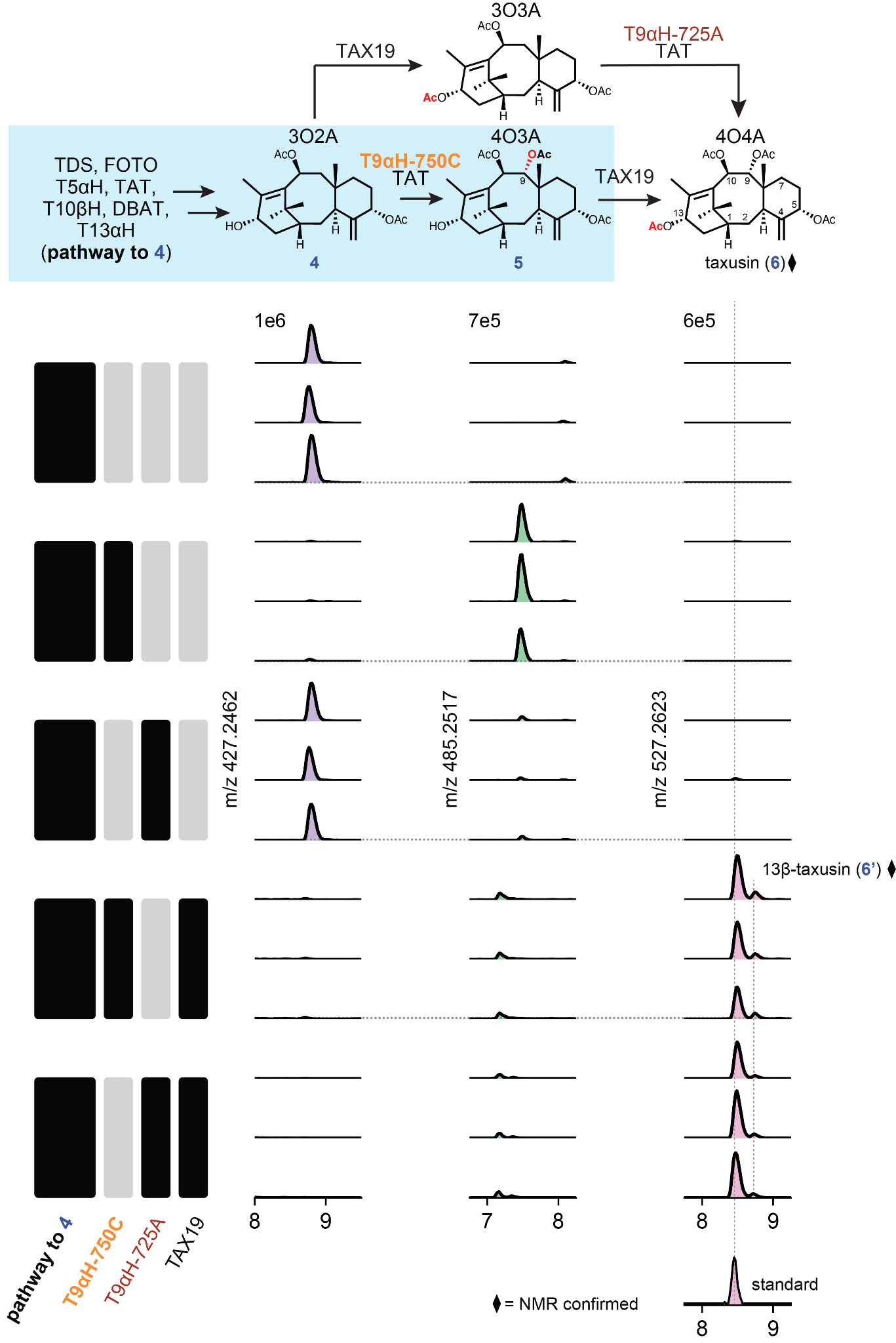
**

**Fig. S9.** **Replicate EIC for Figure 4b.** Extracted ion chromatogram (EIC) of indicated masses of extracts from three replicate *N. benthamiana* leaves expressing the indicated enzyme combinations. Blue shade highlights the biosynthetic pathway toward Taxol.

**
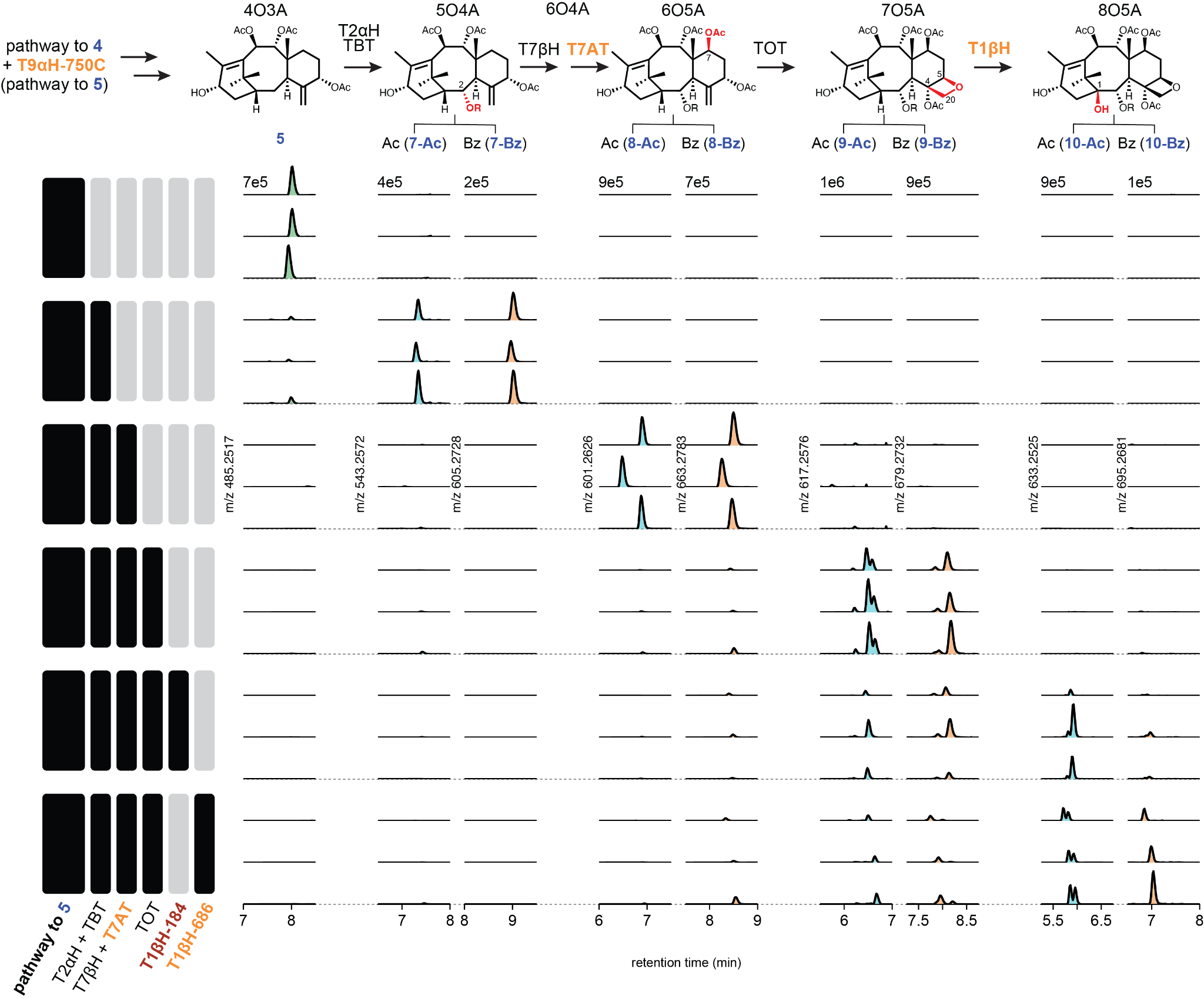
Fig. S10.** **Replicate EIC for Figure 4c.** Extracted ion chromatogram (EIC) of indicated masses of extracts from three replicate *N. benthamiana* leaves expressing the indicated enzyme combinations.


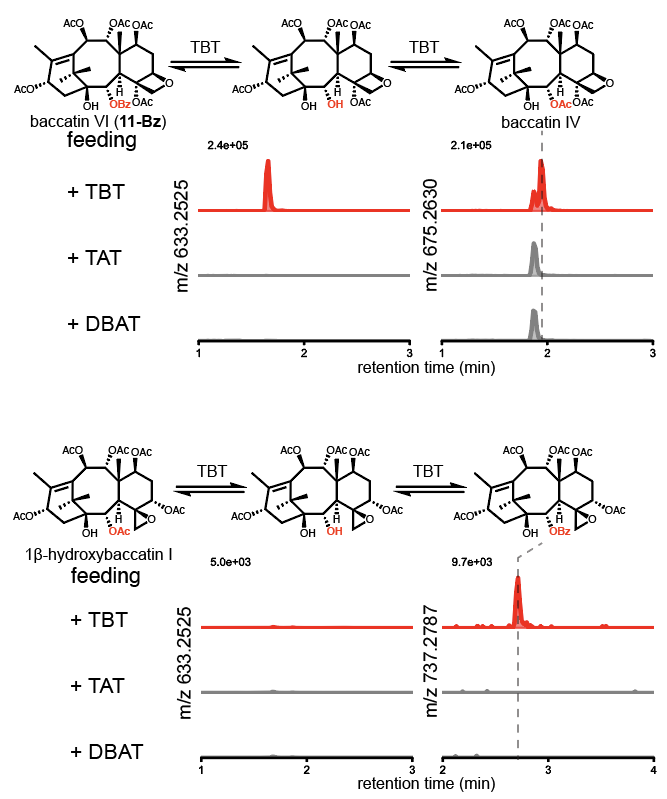


**Fig. S11**. **TBT mediates the interconversion between C-2α-benzoyl and acetyl group.** Baccatin VI (**11-Bz**) and 1β-hydroxybaccatin I were fed into *N. benthamiana* leaves expressing either TBT, TAT, or DBAT. Only TBT showed 2-*O*-debenzoylation activity and converted baccatin VI (**11-Bz**) into C-2α-debenzoylated and C-2α-debenzoylated acetylated products. TBT also converted 1β-hydroxybaccatin I into the corresponding C-2α-deacetylated benzoylated products; the deacetylated intermediate was not detected. The 2-*O*-debenzoylation activity of TBT has been characterized.[^34^](https://paperpile.com/c/CAQ5G4/ap2Fh)


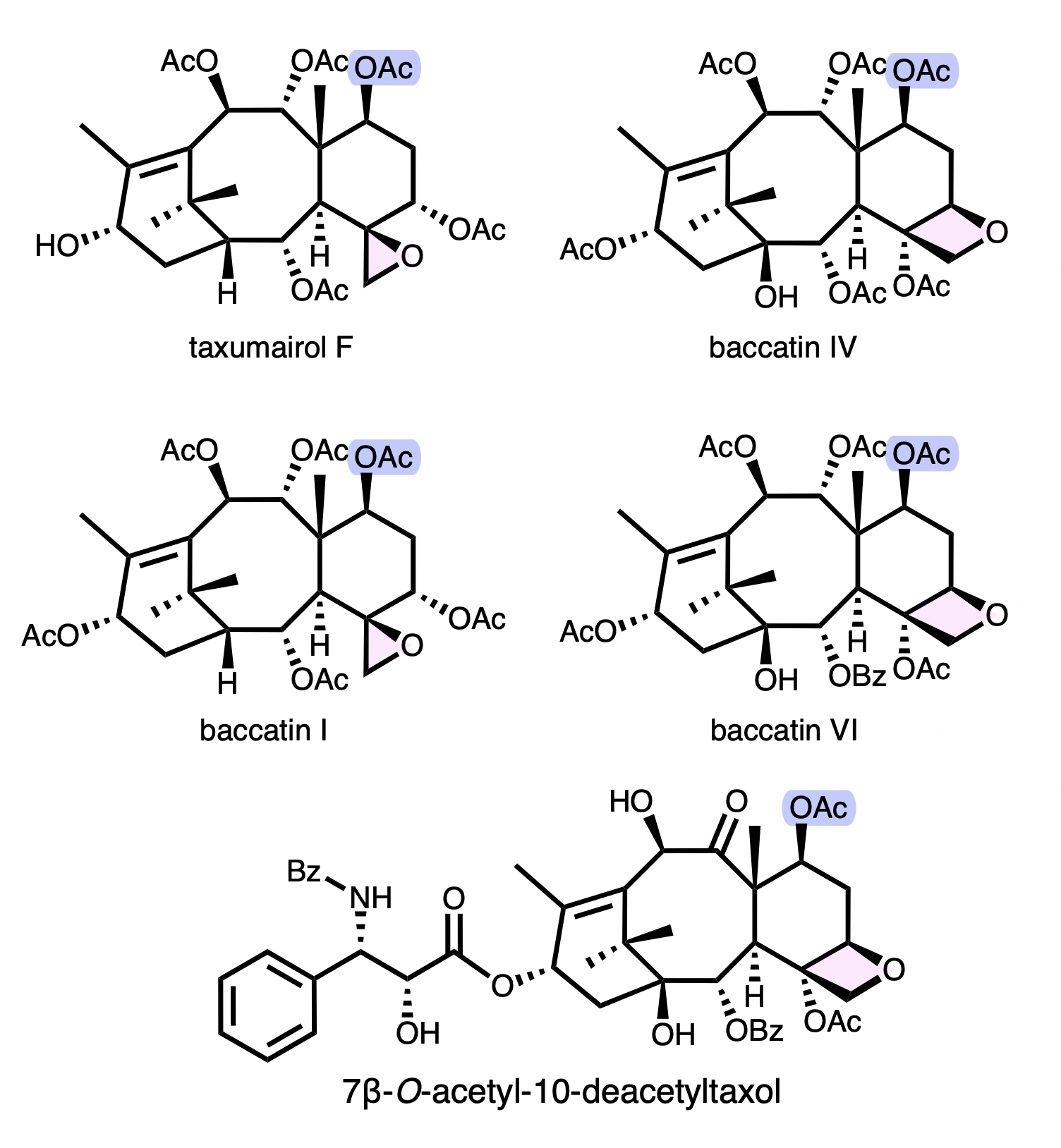


**Fig. S12. Structures of C-7β-acetylated taxane examples with oxetane or epoxide modification.** Many highly oxygenated taxanes with oxetane or epoxide group (highlighted in pink) derived from TOT activity are also C-7β-acetylated (highlighted in blue), including taxumairol F, baccatin I, baccatin IV, baccatin VI, and 7β-*O*-acetyl-10-deacetyltaxol.[^6^](https://paperpile.com/c/CAQ5G4/9Jibm) This leads us to hypothesize that C-7β acetylation is a prerequisite for TOT function.


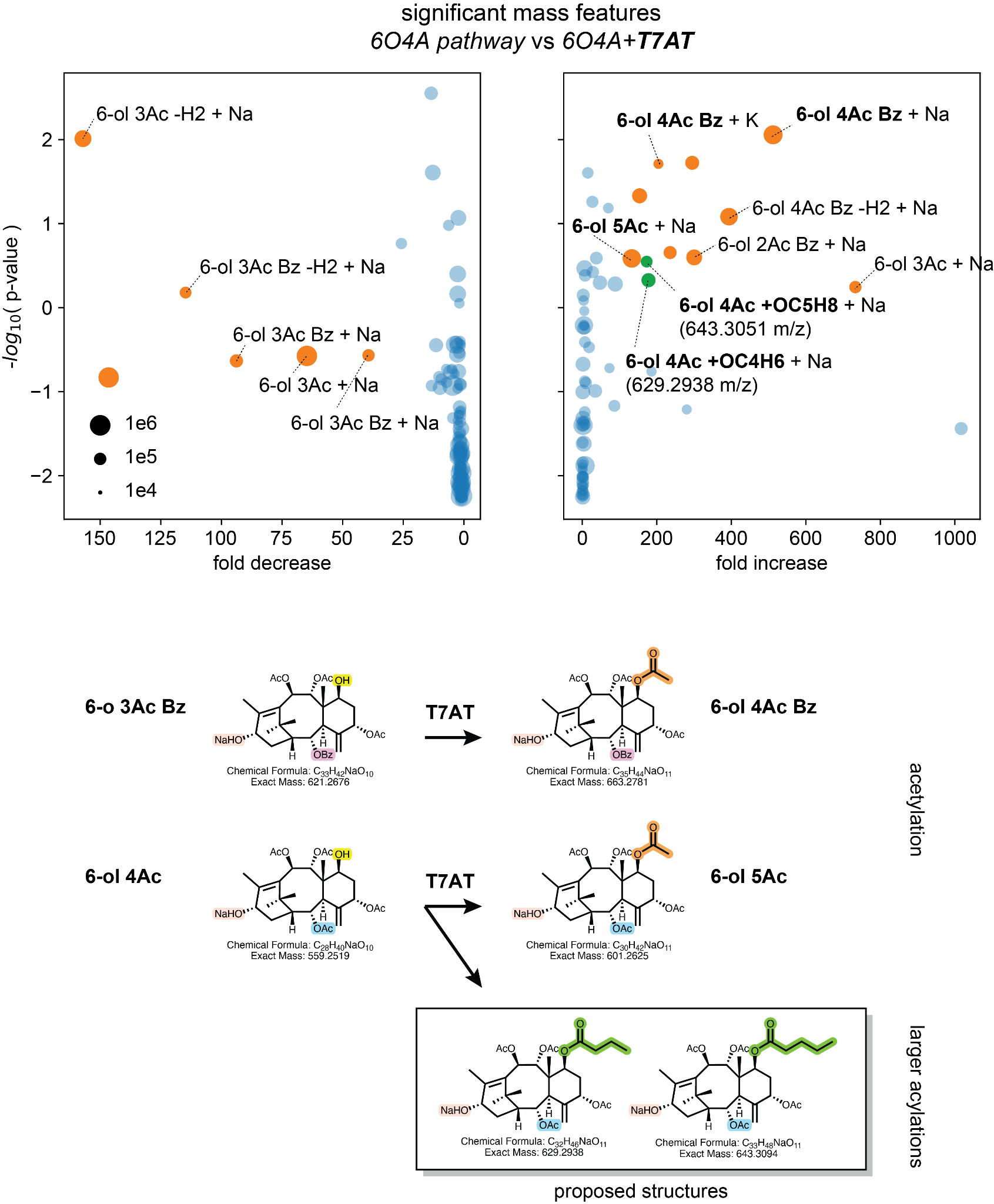


**Fig. S13.** **T7AT is an acyltransferase with multiple acylation activities.** Untransformed volcano plot comparing *N. benthamiana* leaves expressing the pathway to 6O4A with and without T7AT. Each mass feature is shown as a dot whose size indicates EIC integrated area. While the dominant product is the on-pathway 6-ol 4Ac Bz (m/z 663.2781 for M+Na adduct), additional minor products (green dots) correspond to the masses of C4 and C5 acylation products, suggesting that T7AT is capable of multiple acylation activities. Possible acylation structures are illustrated. Mass features corresponding to loss of two protons (-H2), which are likely C-13-ketone derivative,[^1^](https://paperpile.com/c/CAQ5G4/vB77) also show significant changes. P-values calculated by t-test with a Bonferroni correction for multiple hypothesis testing.

**
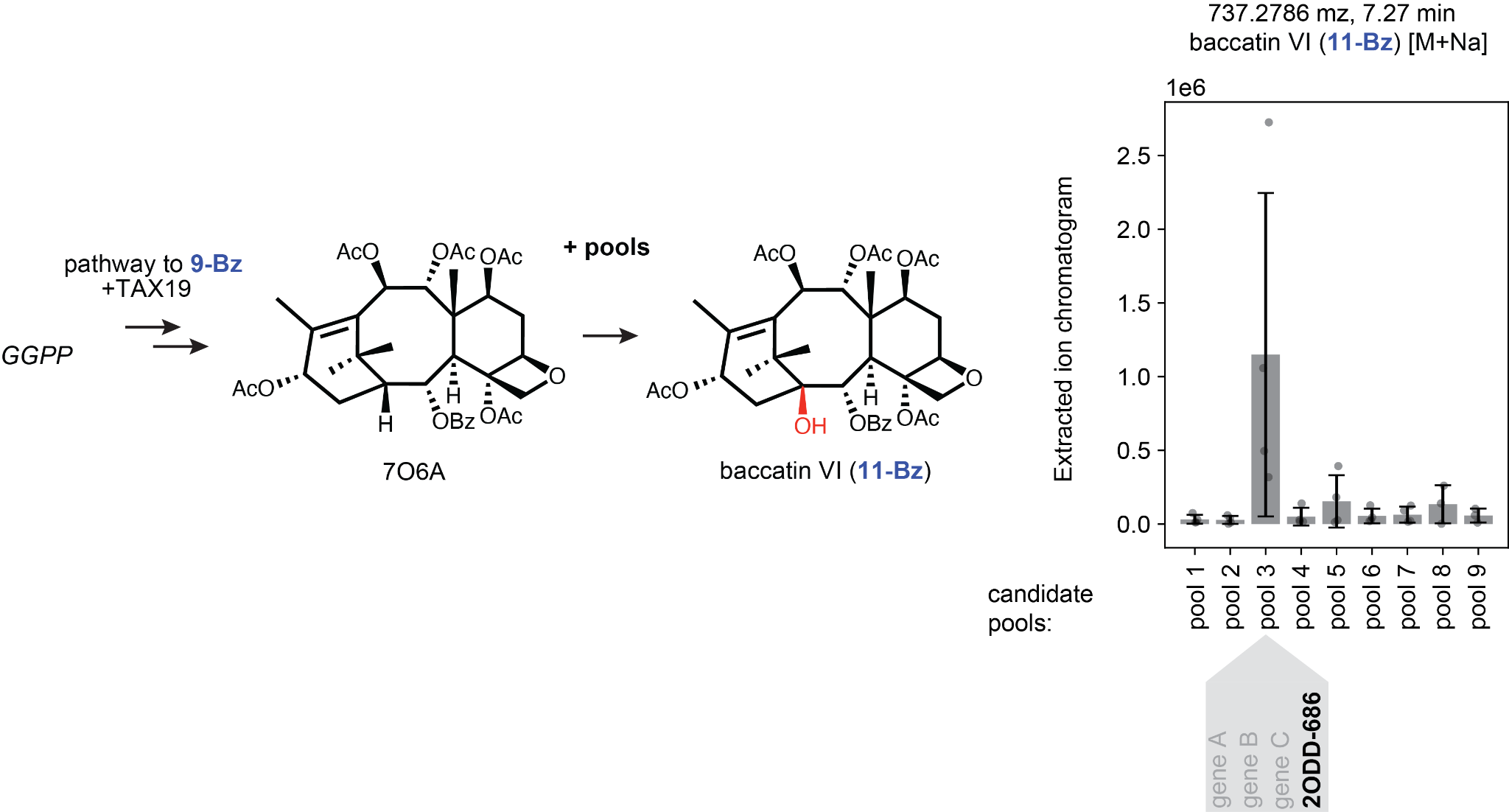
**

**Fig. S14**. **Representative batch screening of T1βΗs candidates**. Candidate genes were screened in pools of 4 genes by co-expression with pathway enzymes in *N. benthamiana*. T1βΗ-686 (2ODD-686) was originally identified by oxidation of 7O6A to baccatin VI (**11-Bz**)**.**


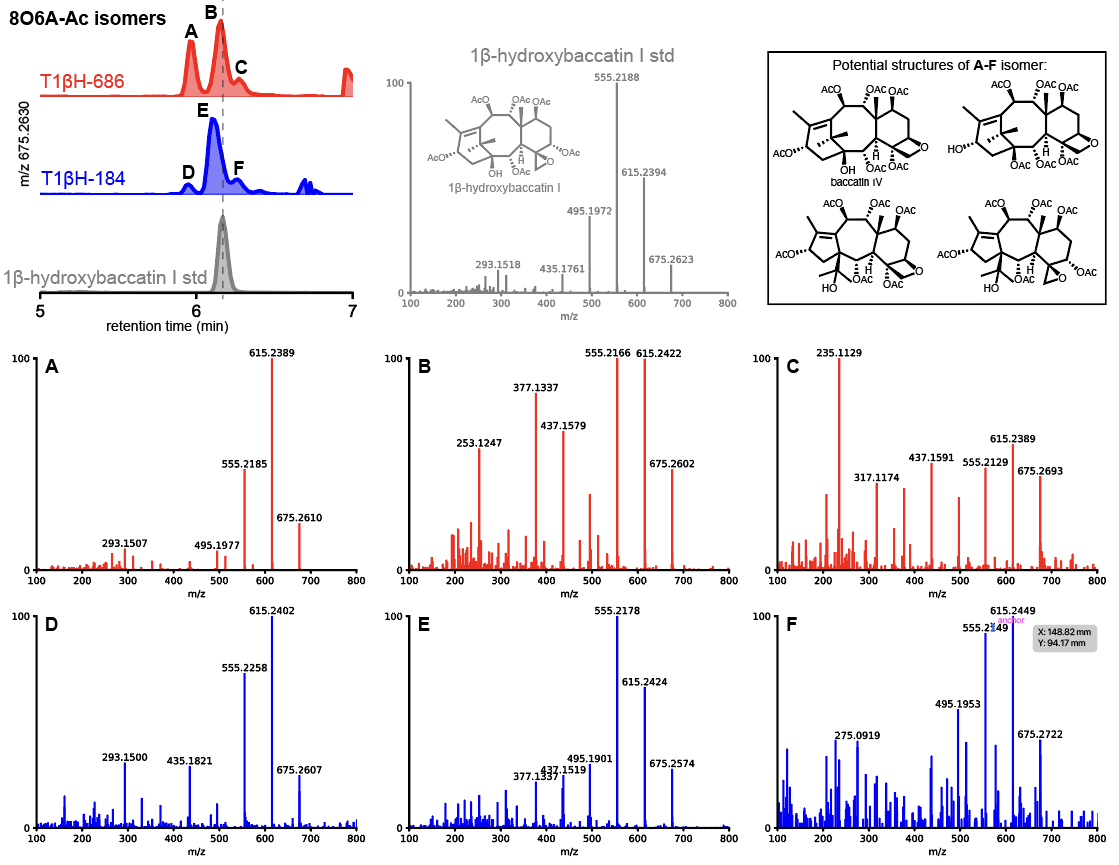


**Fig. S15**. **Production of hexaoxygenated hexacetylated (8O6A-Ac) isomers by the two T1βΗs**. Both T1βH-184 and T1βH-686 produce 3 major 8O6A-Ac products (peak A-F). MSMS fragmentation patterns of all products and 1β-hydroxybaccatin I standard are shown. MSMS fragmentations were generated using [M+Na]^+^ (m/z = 675.2630) as the precursor ion and fragmented with a collision energy of 30 eV. While peak B shares a similar retention time as 1β-hydroxybaccatin I, they show different MSMS patterns, thus are deemed as different compounds. The following gene sets were heterologously expressed in *N. benthamiana* via *Agrobacterium-*mediated transient expression:tHMGR, GGPPS, TDS, FoTO1, T5αH, TAT, T10βH, DBAT, T13αΗ, TAX19, T2αΗ, ΤΒΤ, Τ7βΗ, Τ7ΑΤ, ΤΟΤ, T1βH-184 or T1βΗ-686.

**
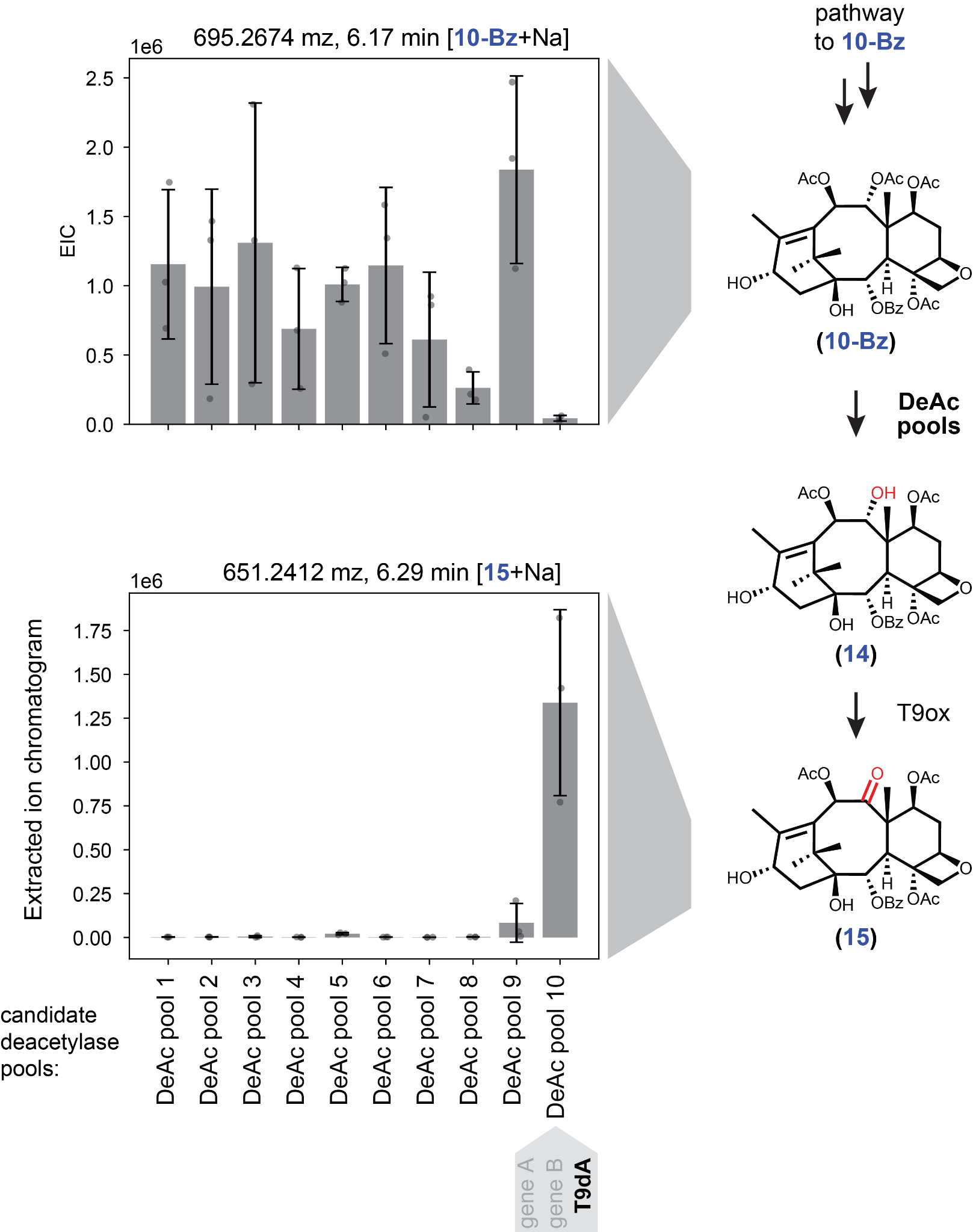
Fig. S16**. **Representative batch screening of deacetylase candidates**. Candidate genes were screened in pools of 3-4 genes by co-expression with pathway enzymes in *N. benthamiana*. T9dA was originally identified by deacetylation of **10-Bz** to **14**, and, due to inclusion of T9ox in this screen, accumulation of 7β-*O*-acetyl-baccatin III (**15**)**.**

**
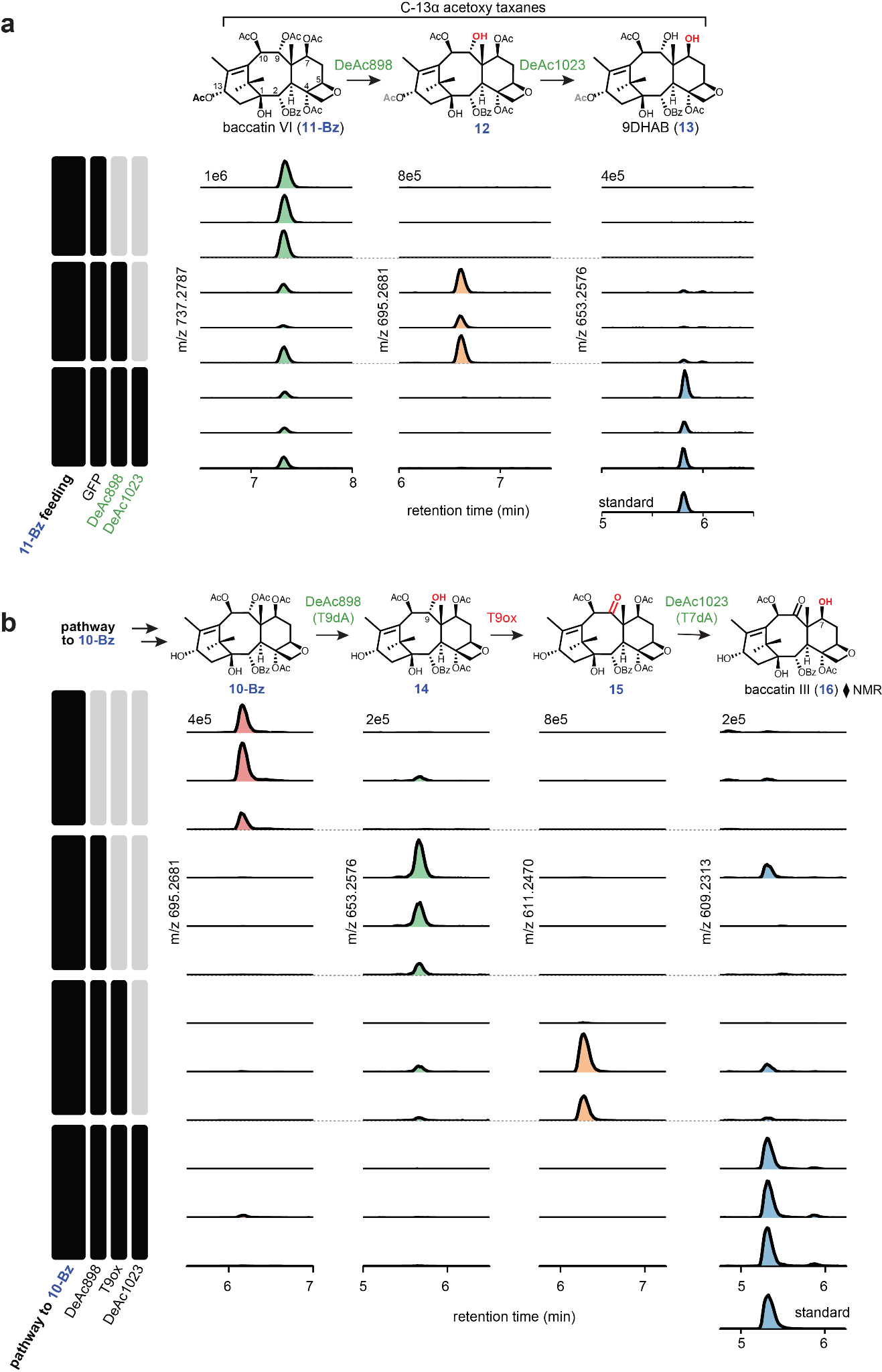
**

**Fig. S17.** **Replicate EIC for Figure 5.** Extracted ion chromatogram (EIC) of indicated masses of extracts from three replicate *N. benthamiana* leaves expressing the indicated enzyme combinations. (**a**) Stepwise deacetylation and oxidation of fed baccatin VI (**11-Bz**) to 9-dihydro-13α-acetylbaccatin III (9DHAB, **13**). Related to **Extended Data Figure 6a**. (**b**) Replicates of the four steps in reconstituted baccatin III biosynthesis, starting from the reconstituted pathway of **10-Bz**. Related to **Fig. 5c**.

**
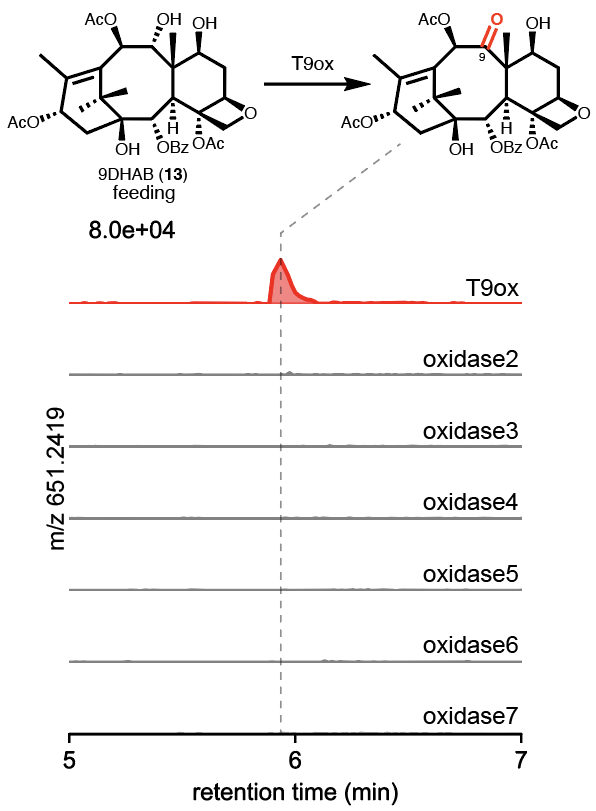
**

**Fig. S18**. **Screening of oxidases identified the taxane C-9 oxidase (T9ox)**. *N. benthamiana* leaves expressing individual candidate oxidase via *Agrobacterium-*mediated infiltration and fed with 9DHAB (**13**) at 3 day post infiltration (dpi). Over 30 P450s and 2-ODDs were screened in batches and the only active batch with 8 candidates was deconvoluted as shown here. EIC of C-9 ketone product (m/z 651.2419) was shown.

**
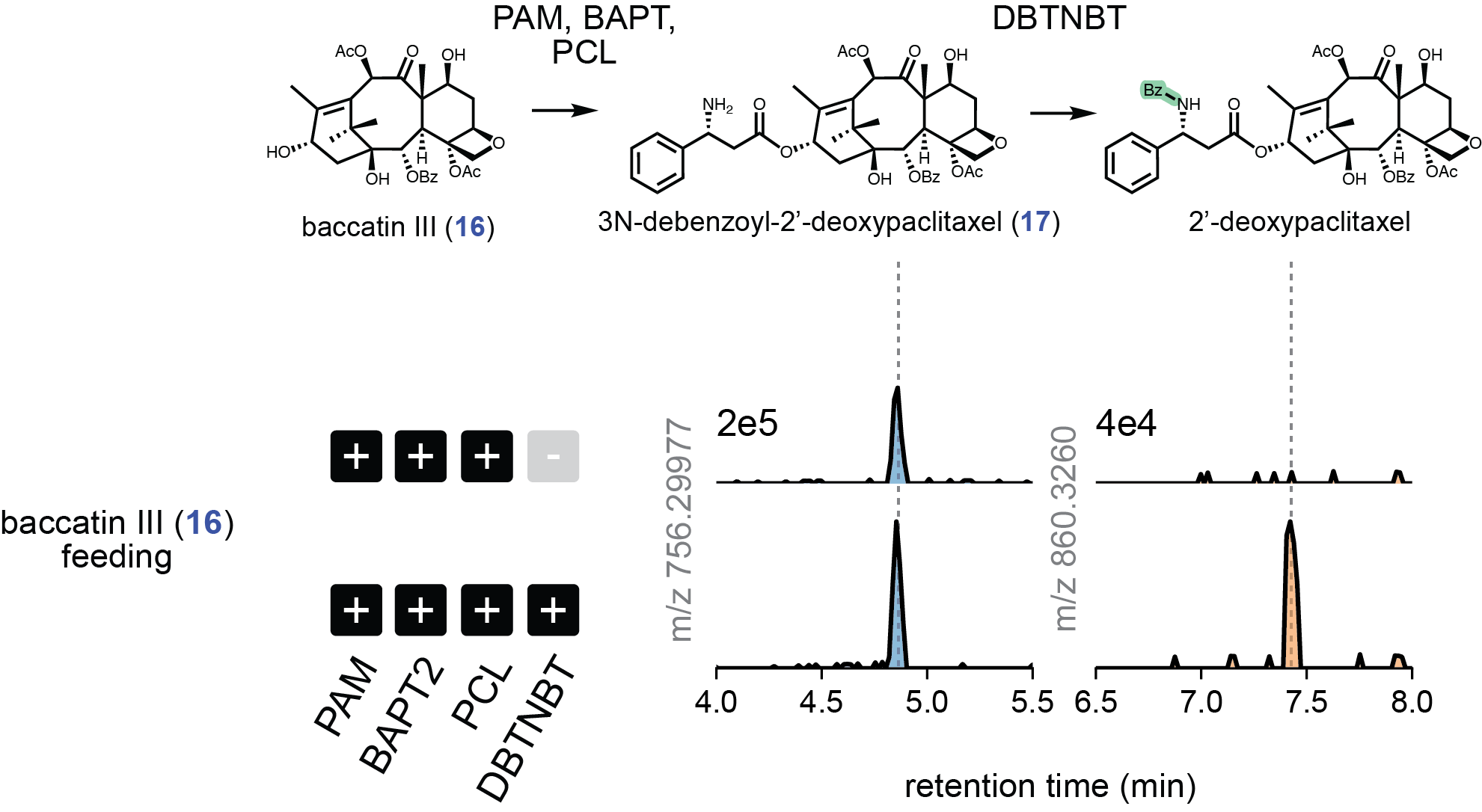
**

**Fig. S19**. **Production of putative 2’-deoxypaclitaxel at low levels**. EICs of *N. benthamiana* leaves expressing side chain enzymes (PAM, BAPT, PCL) with or without DBTNBT via *Agrobacterium-*mediated infiltration and fed with 25 μM baccatin III (**16**) at 3 day post infiltration (dpi). DBTNBT yields a new product whose mass matches the expected product, 2’-deoxypaclitaxel The low conversion from 3’-*N*-debenzoyl-2’-deoxypaclitaxel (**17**) to 2’-deoxypaclitaxel suggests that 2’-α-hydroxylation is a prerequisite for 3’-*N*-benzoylation, which is consistent with previous hypothesis[^35^](https://paperpile.com/c/CAQ5G4/gOhBC).

**
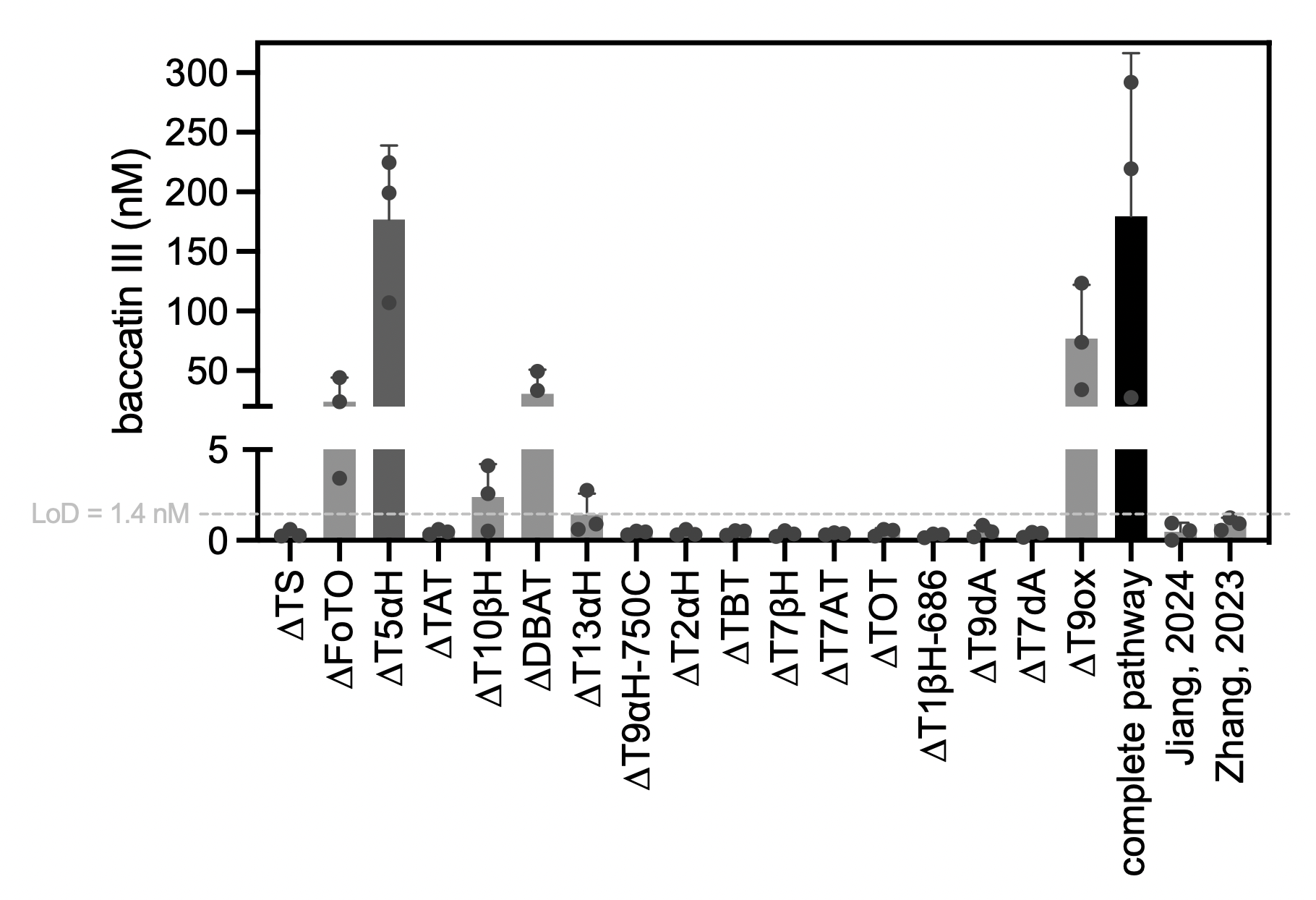
**

**Fig. S20. Bar graph of baccatin III (16) concentrations of the samples in Fig. 5i.** Baccatin III (**16**) concentration of *N. benthamiana* leaves expressing the indicated strain combinations via *Agrobacterium-*mediated infiltration. Leaf discs (6*1 cm diameter per sample; average dried weight = 10 mg) were harvested and extracted with 700 uL extraction buffer (75% ACN/water) and subjected to Agilent 6470 QQQ LCMS analysis. Limit of detection (LoD) is calculated using equation LoD = 3.3*SD/slope of calibration, where SD is the standard deviation (SD) of low concentration samples (10 pM baccatin III standard, n=10). Data are shown as the mean ± standard deviation, n = 3. See **Methods** and **Source Data** for detailed experimental setup and raw values.

**
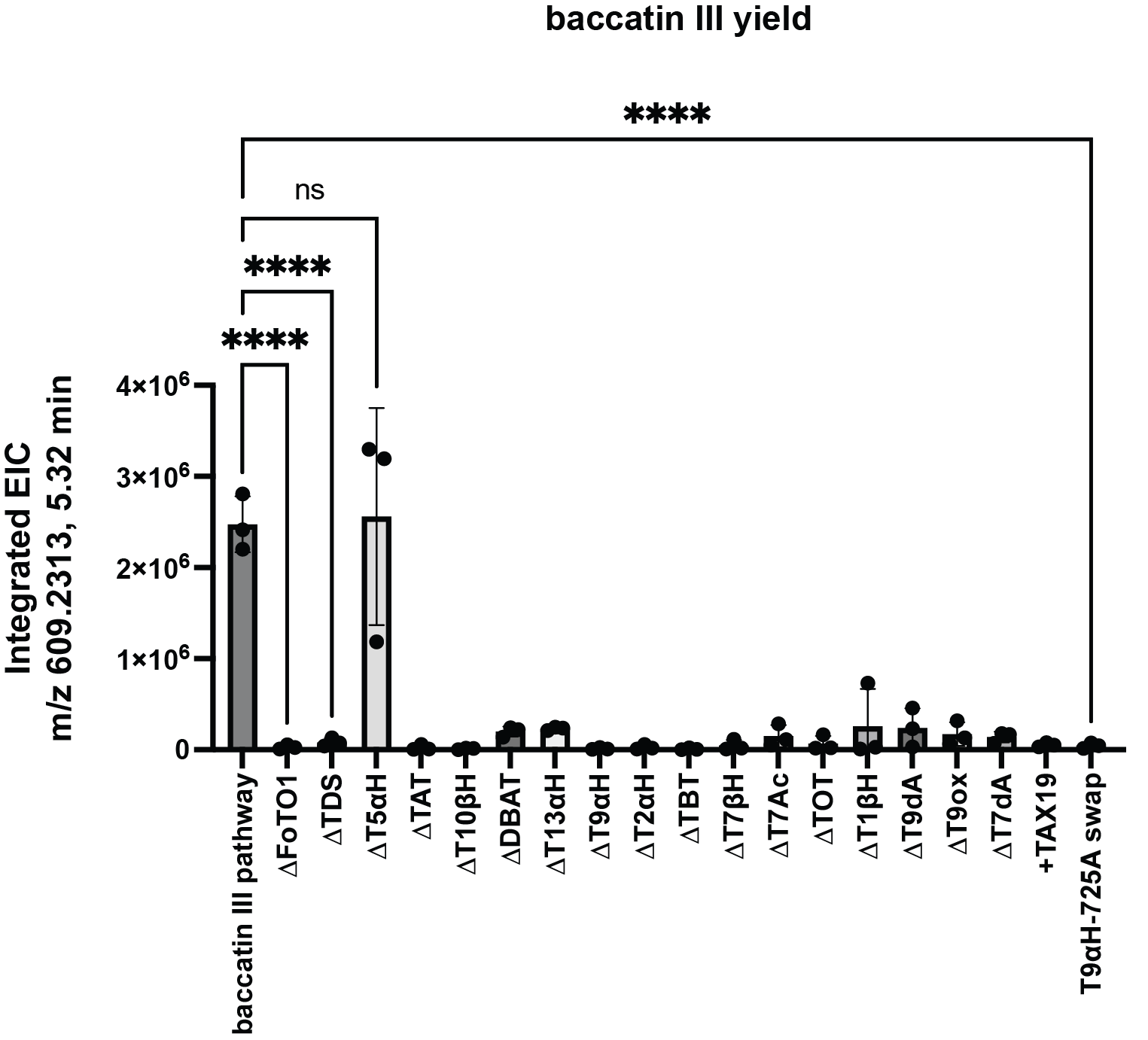
**

**Fig. S21. Independent replicate data for full-pathway gene dropout experiment.** EIC peak area of baccatin III (**16**) from *N. benthamiana* leaves expressing the full 17-gene baccatin III pathway, as well as single gene dropouts, addition of Tax19, or exchanging our T9αH-750C for the alternative T9αH-725A. Samples were subjected to Agilent 6520 Q-TOF LCMS analysis. Significance indicates results of an ordinary one-way ANOVA comparison to the full pathway (**** indicates p < 0.0001).

**
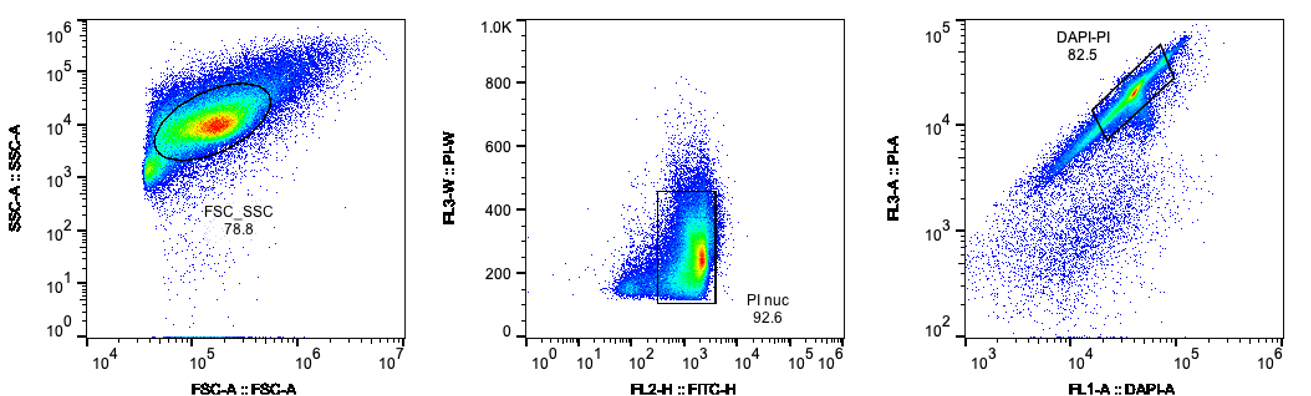
**

**Fig. S22.** **Gating strategy for nuclei sorting**. FACS plots generated with FlowJo v10 indicating the three step gating strategy to sort intact Taxus nuclei. Following the tissue disruption and lysate filtering outlined in **Methods**, nuclei are stained with 5ng/uL 4,6-diamidino-2-phenylindole (DAPI) and 5ng/uL propidium iodide (PI). Nuclei are gated on three subsequent gates: (i) size selection using forward scatter (FSC) vs side scatter (SSC), (ii) singlet selection using PI fluorescence height and width, and (iii) co-staining with DAPI and PI to identify clean nuclei.

# Supplementary Figures - NMR spectrum


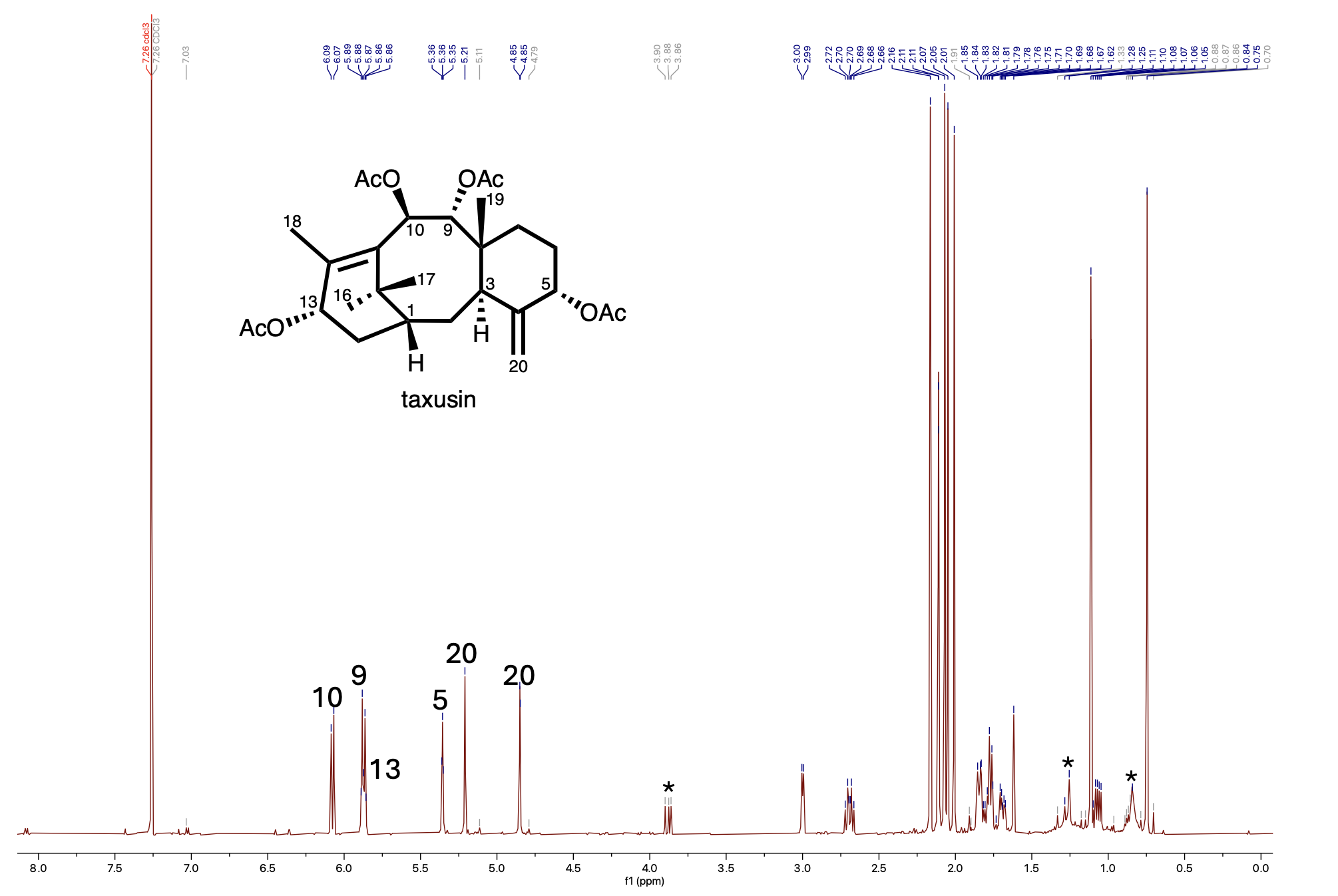


**Fig. S23. ^1^H-NMR spectrum of taxusin (6) (CDCl_3_, 600 ΜΗz, 298 K).** Asterisk indicates impurities.


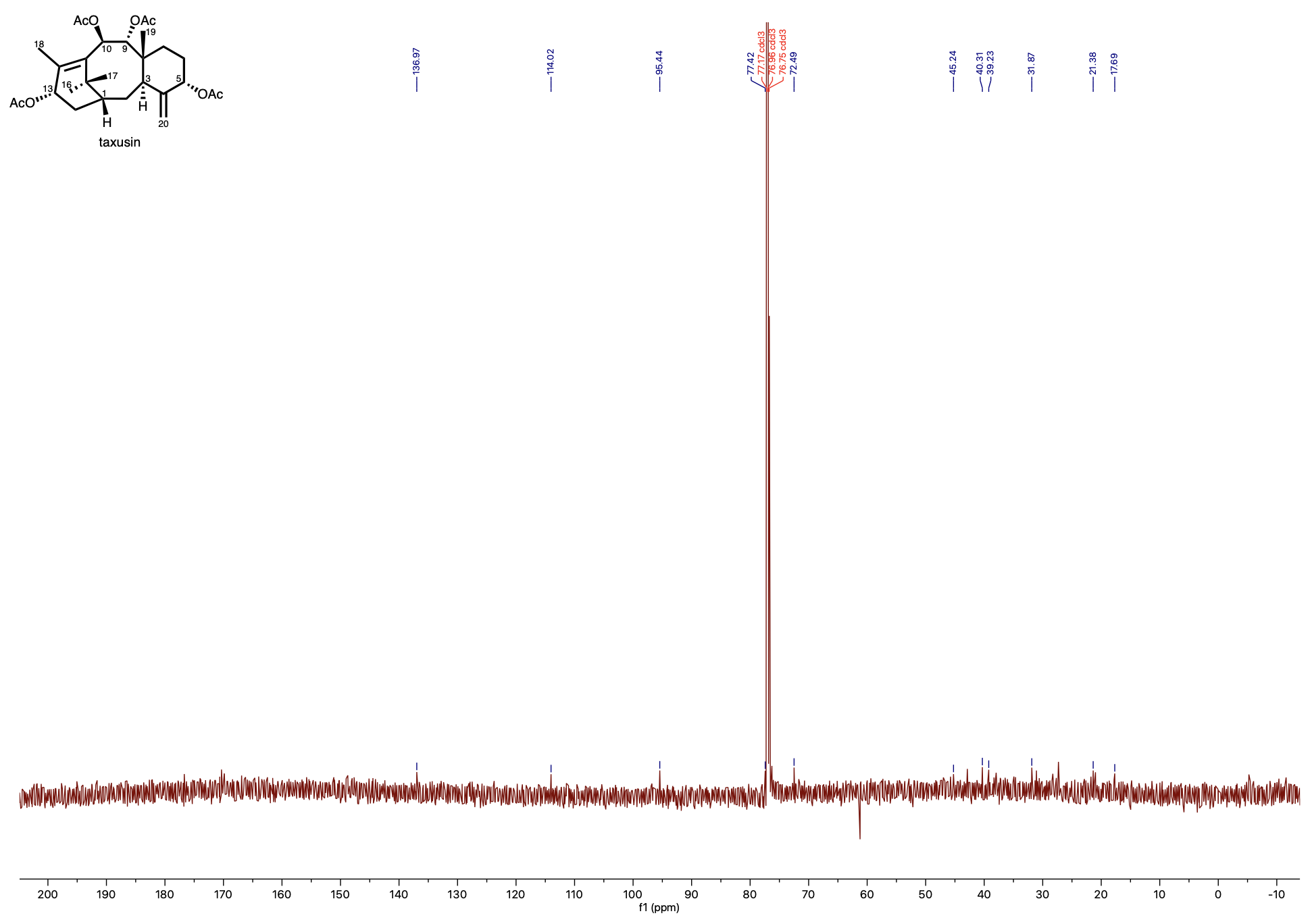


**Fig. S24. ^13^C-NMR spectrum of taxusin (6) (CDCl_3_, 600 ΜΗz, 298 K).**

**
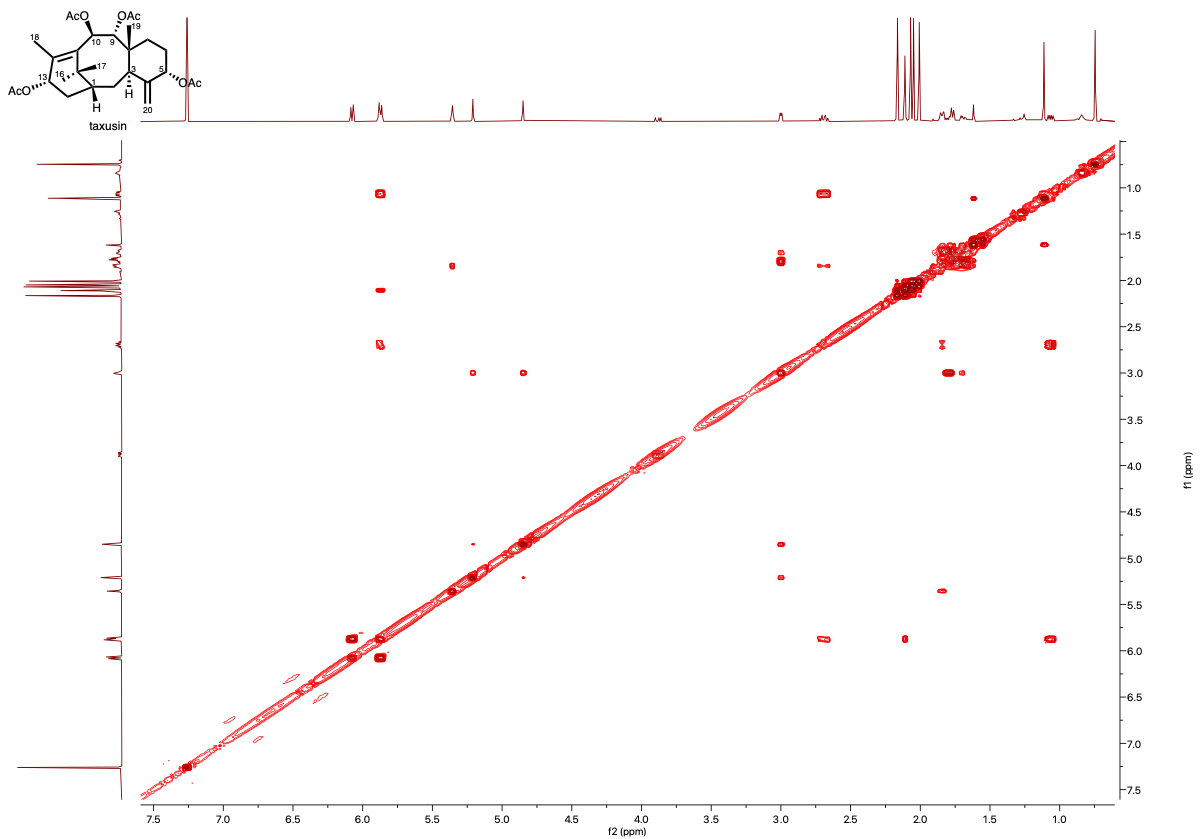
**

**Fig. S25. COSY spectrum of taxusin (6) (CDCl_3_, 600 ΜΗz, 298 K).**

**
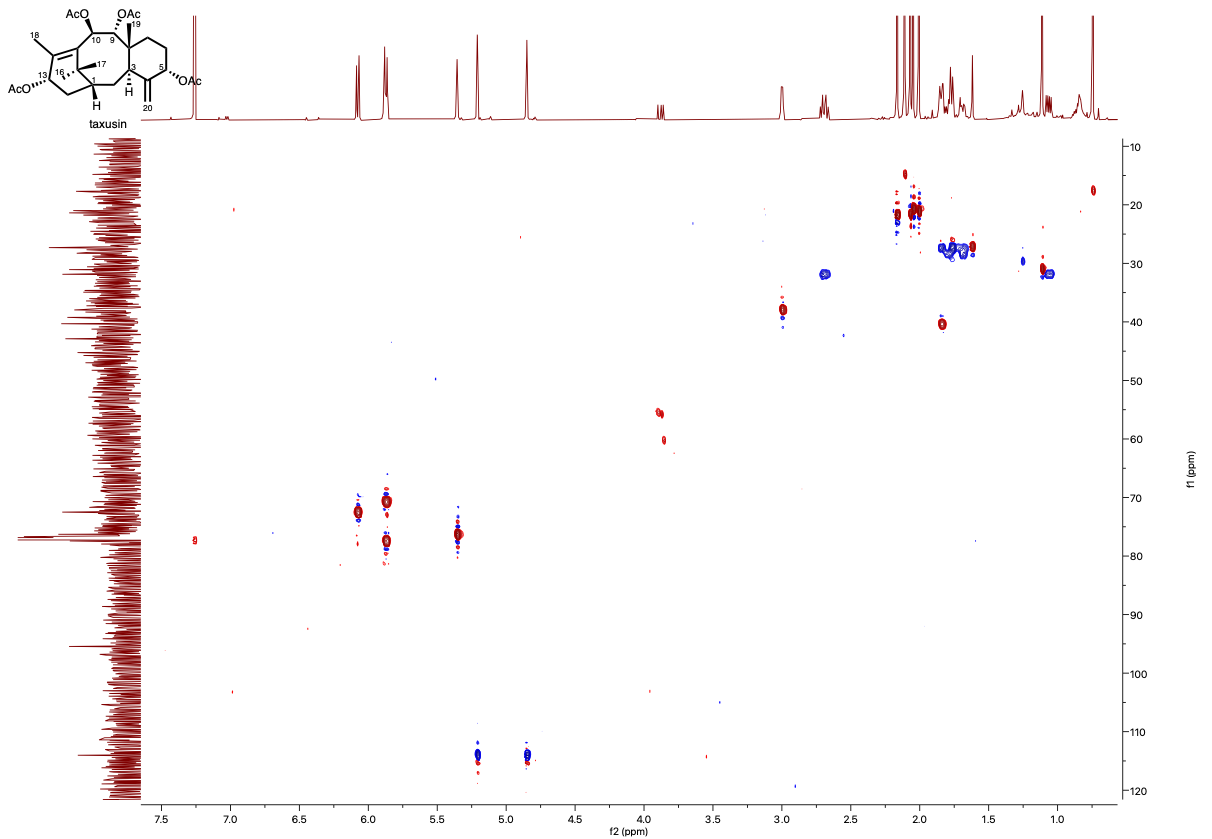
**

**Fig. S26. HSQC spectrum of taxusin (6) (CDCl_3_, 600 ΜΗz, 298 K).**

**
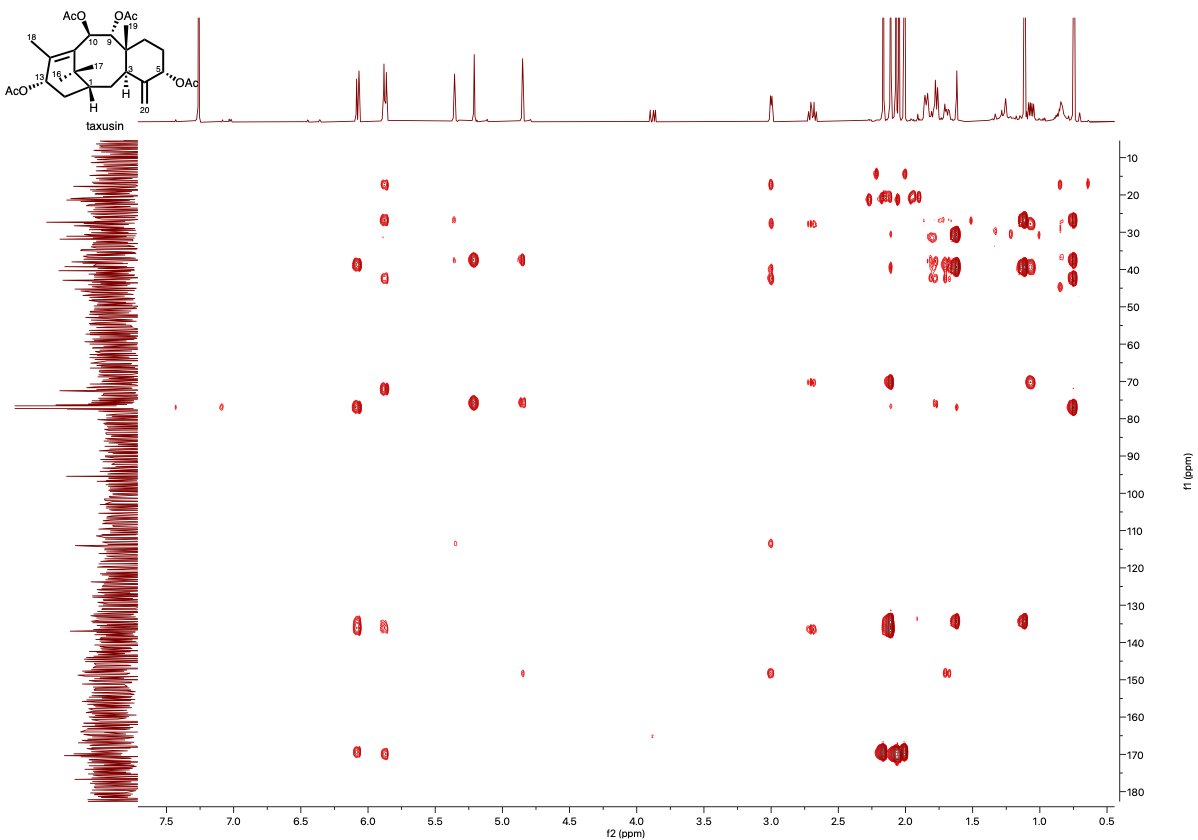
**

**Fig. S27. HMBC spectrum of taxusin (6) (CDCl_3_, 600 ΜΗz, 298 K).**

**
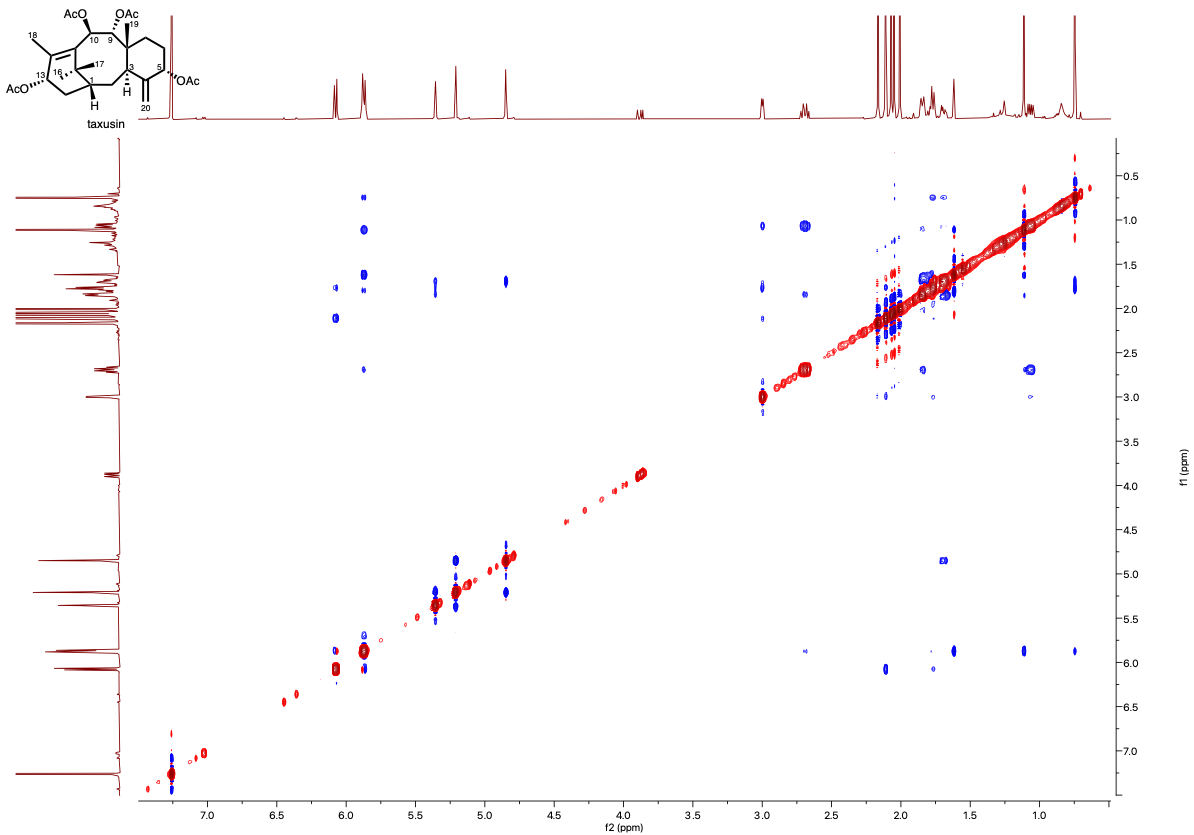
**

**Fig. S28. ROESY spectrum of taxusin (6) (CDCl_3_, 600 ΜΗz, 298 K).**

**
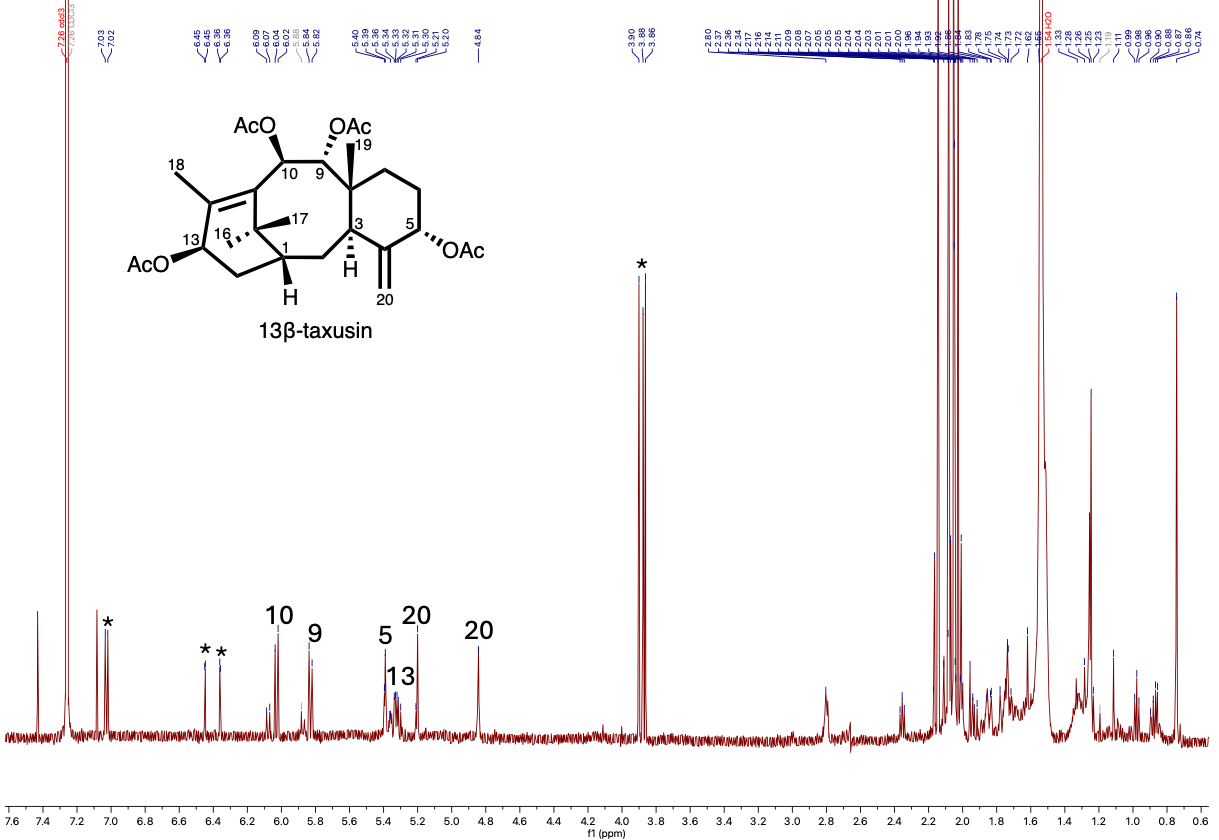
**

**Fig. S29. ^1^H-NMR spectrum of 13β-taxusin (6’) (CDCl_3_, 600 ΜΗz, 298 K).** Asterisk indicates impurities.


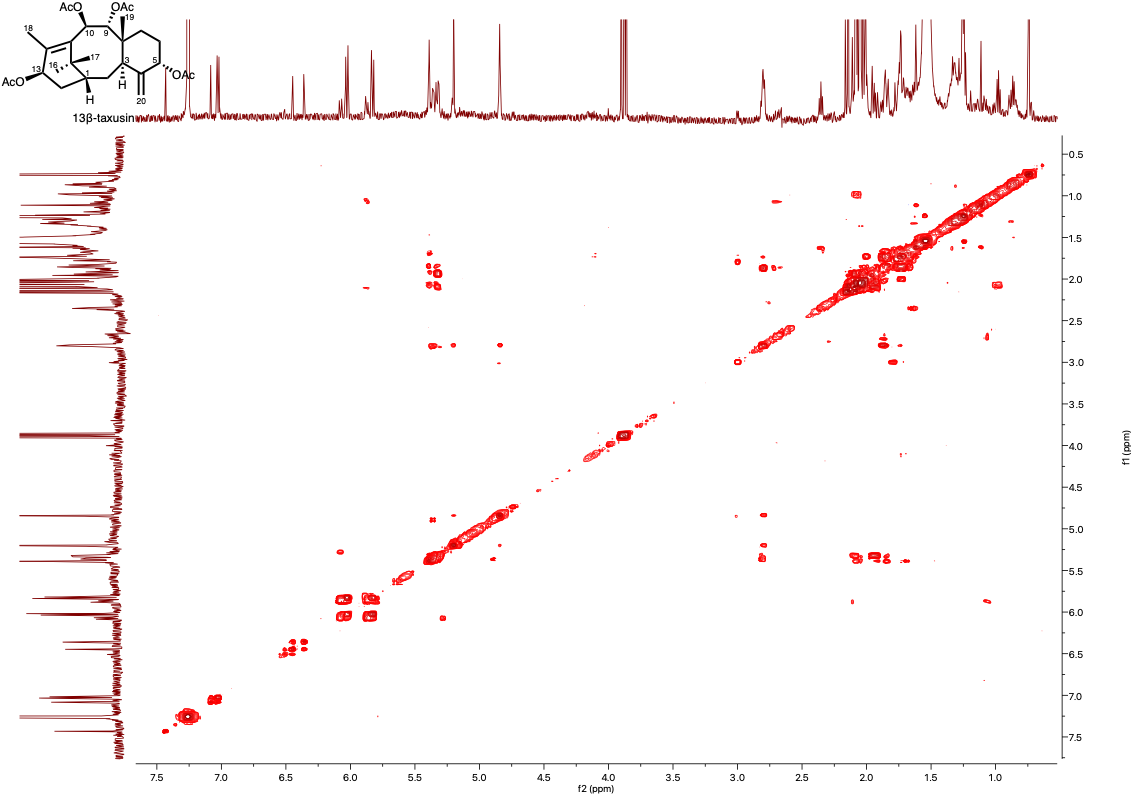


**Fig. S30. COSY spectrum of 13β-taxusin (6’) (CDCl_3_, 600 ΜΗz, 298 K).**

**
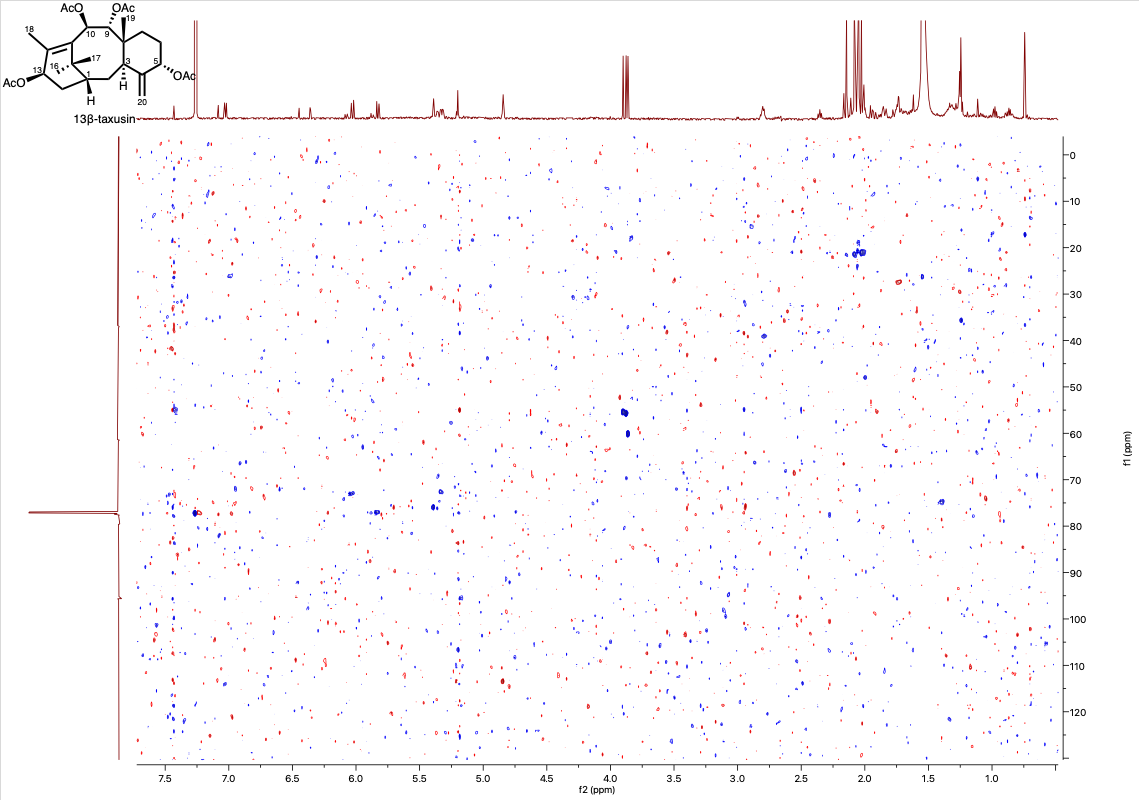
**

**Fig. S31. HSQC spectrum of 13β-taxusin (6’) (CDCl_3_, 600 ΜΗz, 298 K).**

**
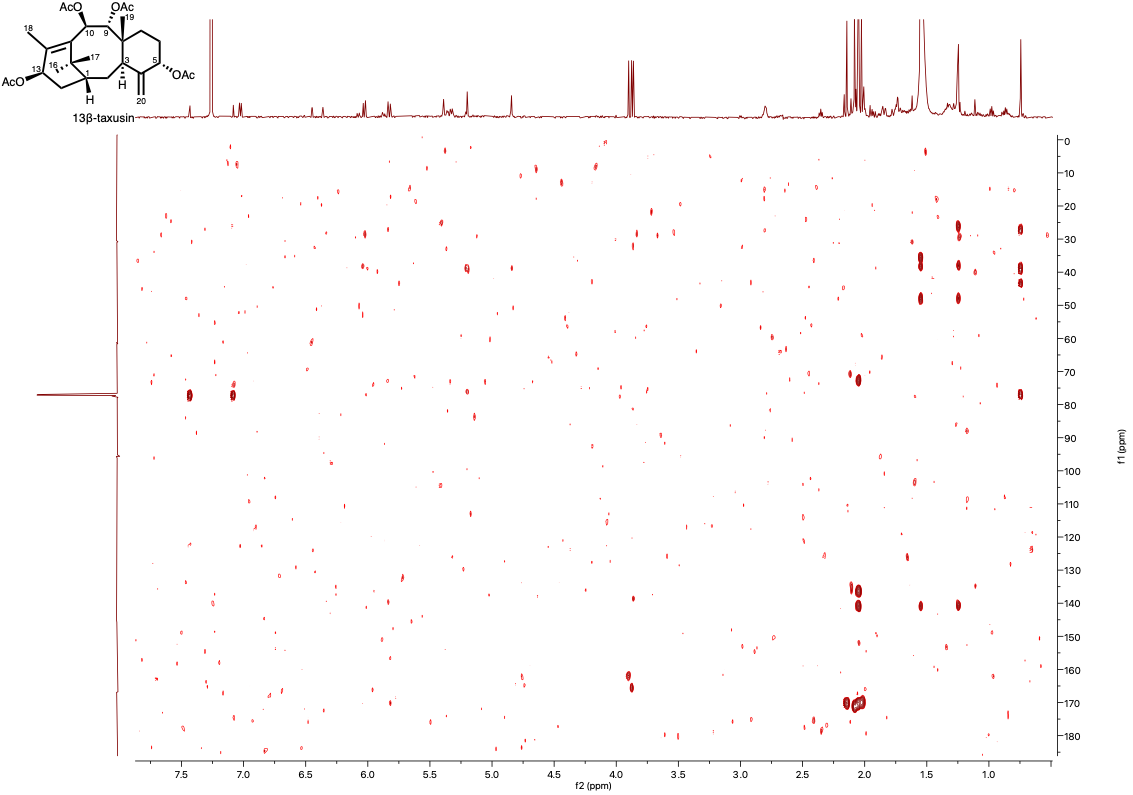
**

**Fig. S32. HMBC spectrum of 13β-taxusin (6’) (CDCl_3_, 600 ΜΗz, 298 K).**

**
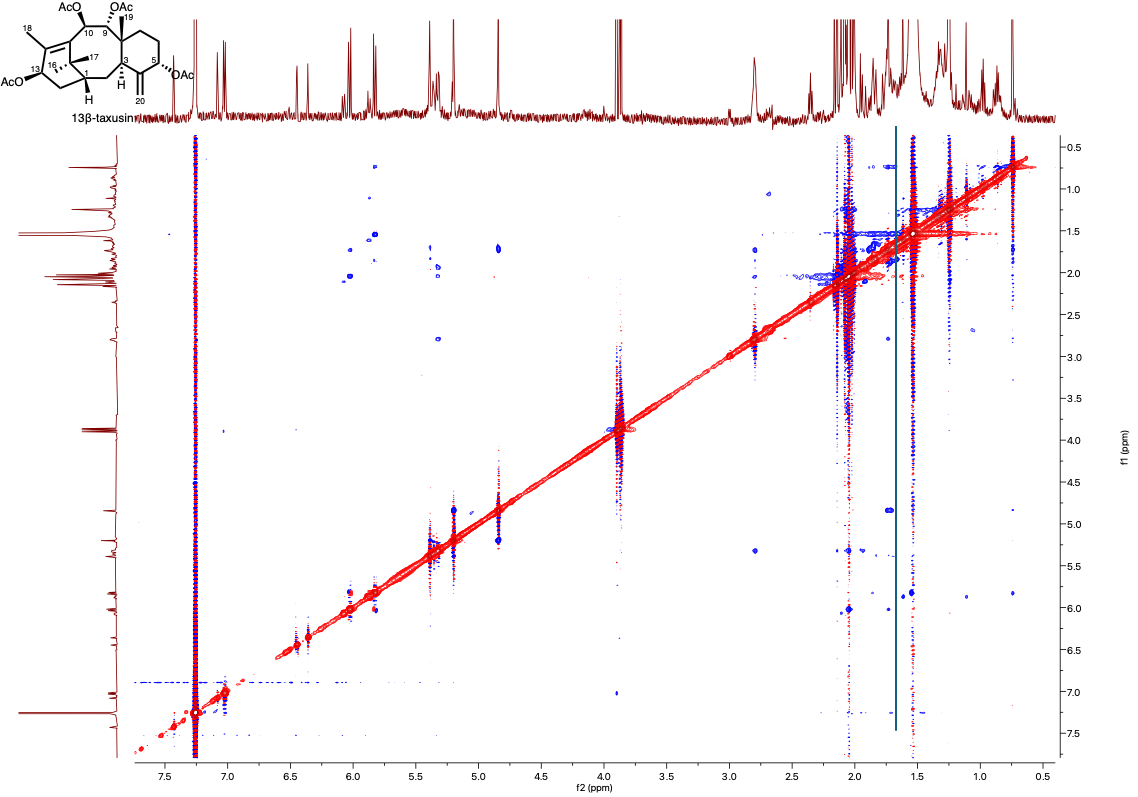
**

**Fig. S33. ROESY spectrum of 13β-taxusin (6’) (CDCl_3_, 600 ΜΗz, 298 K).**

**
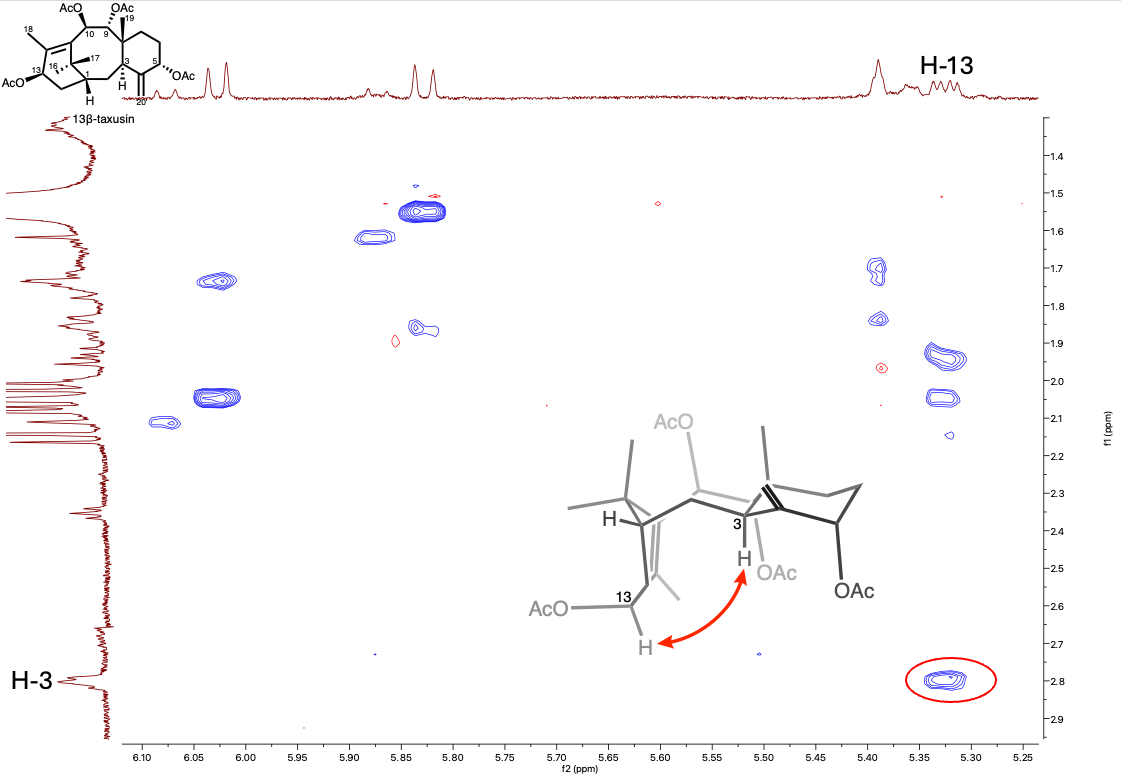
**

**Fig. S34. ROESY spectrum of 13β-taxusin (6’) (CDCl_3_, 600 ΜΗz, 298 K) showing key ROESY correlations.**

**
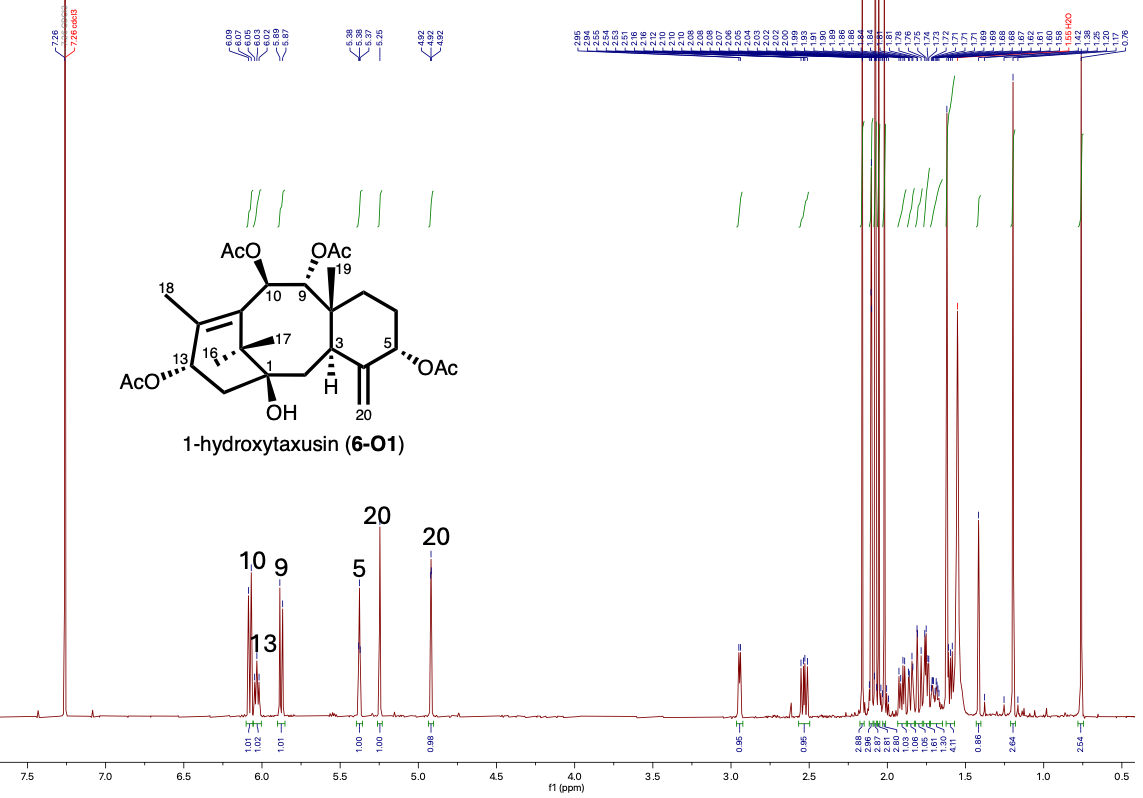
**

**Fig. S35. ^1^H-NMR spectrum of 1β-hydroxytaxusin (6-O1) (CDCl_3_, 600 ΜΗz, 298 K).**

**
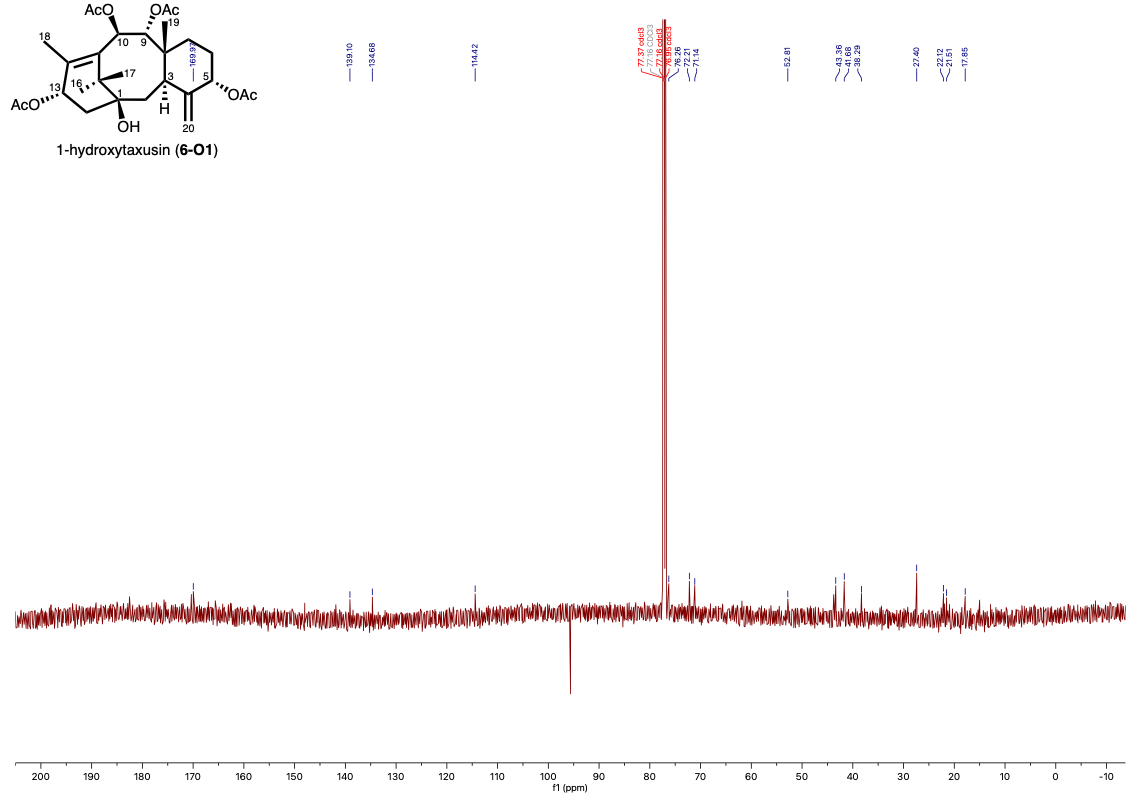
**

**Fig. S36. ^13^C-NMR spectrum of 1β-hydroxytaxusin (6-O1) (CDCl_3_, 600 ΜΗz, 298 K).**

**
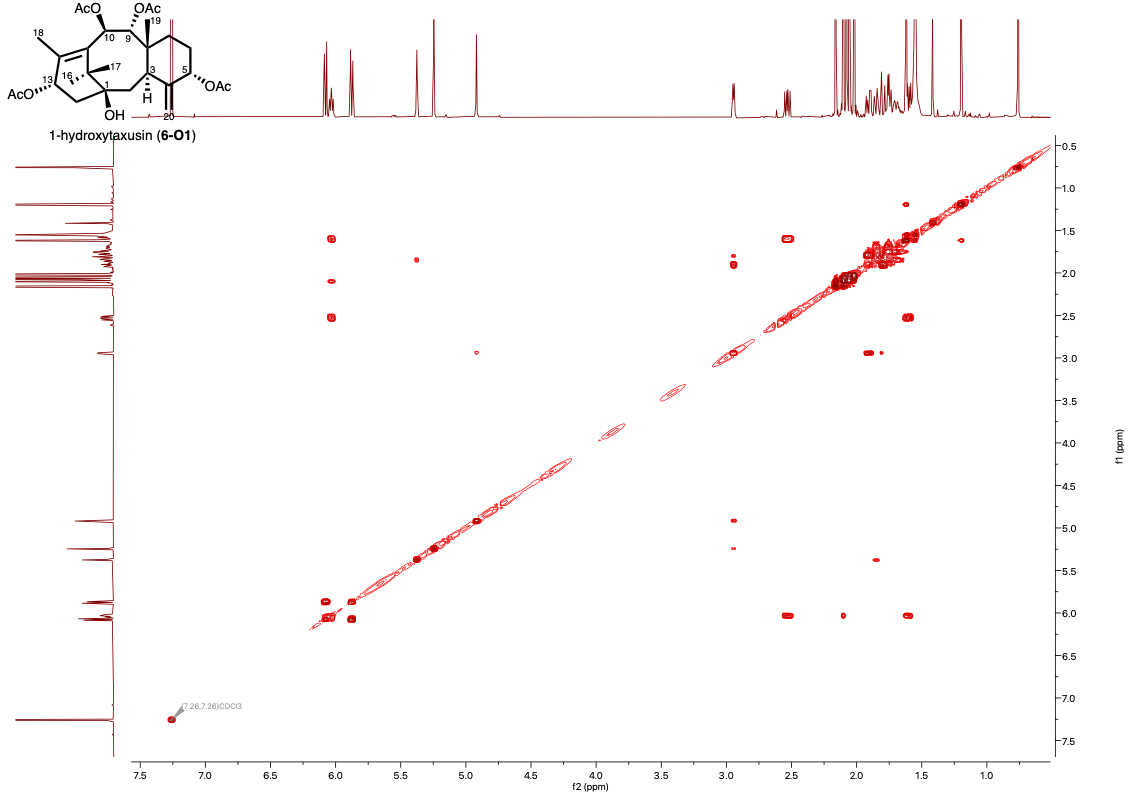
**

**Fig. S37. COSY spectrum of 1β-hydroxytaxusin (6-O1) (CDCl_3_, 600 ΜΗz, 298 K).**

**
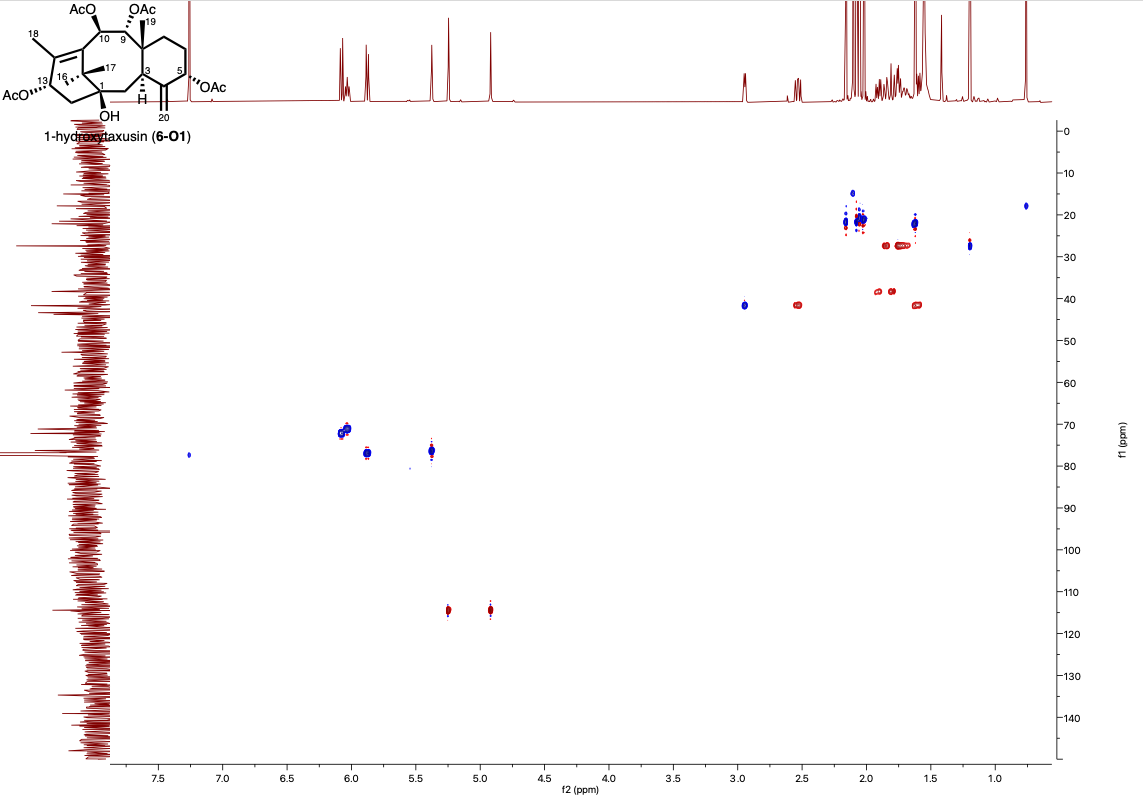
**

**Fig. S38. HSQC spectrum of 1β-hydroxytaxusin (6-O1) (CDCl_3_, 600 ΜΗz, 298 K).**

**
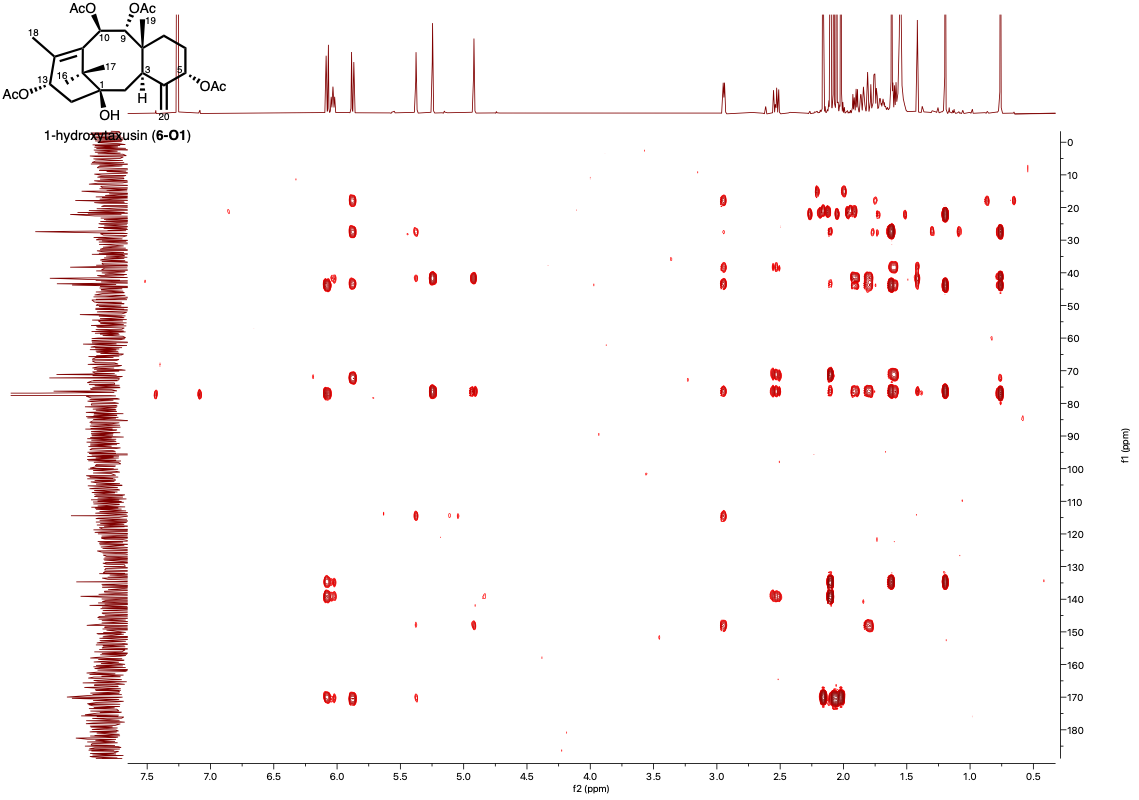
**

**Fig. S39. HMBC spectrum of 1β-hydroxytaxusin (6-O1) (CDCl_3_, 600 ΜΗz, 298 K).**

**
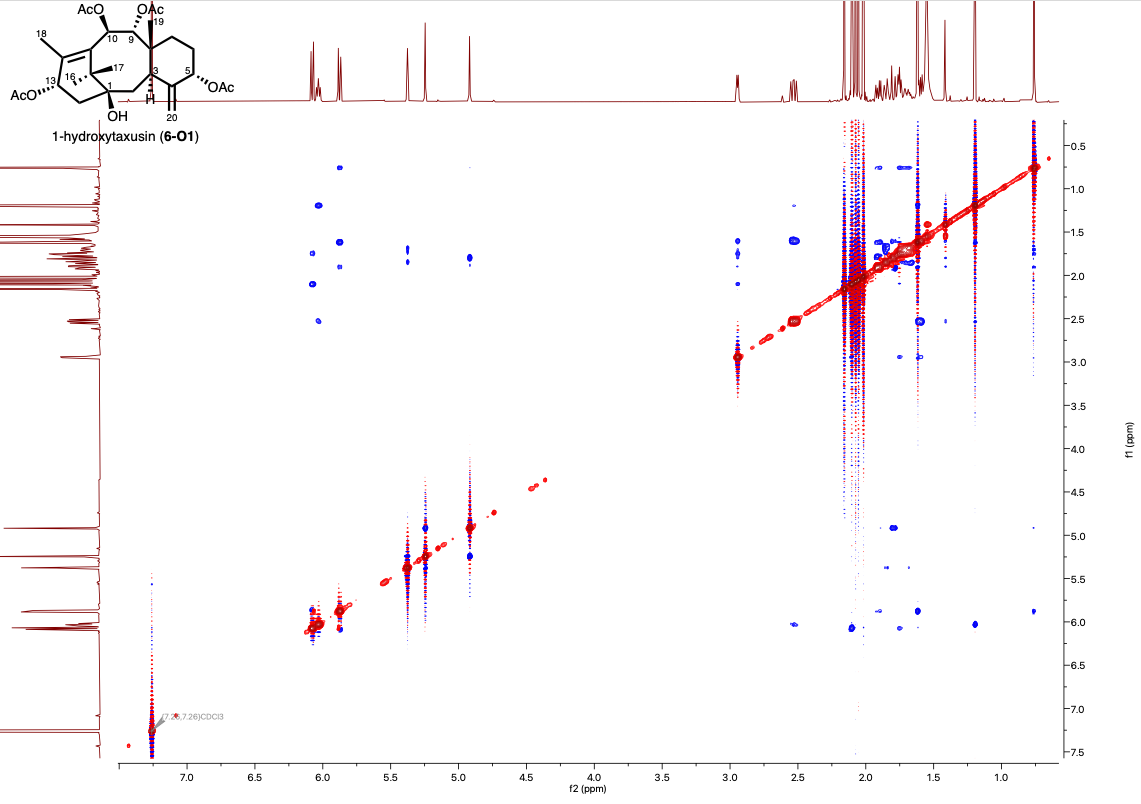
**

**Fig. S40. ROESY spectrum of 1β-hydroxytaxusin (6-O1) (CDCl_3_, 600 ΜΗz, 298 K).**

**
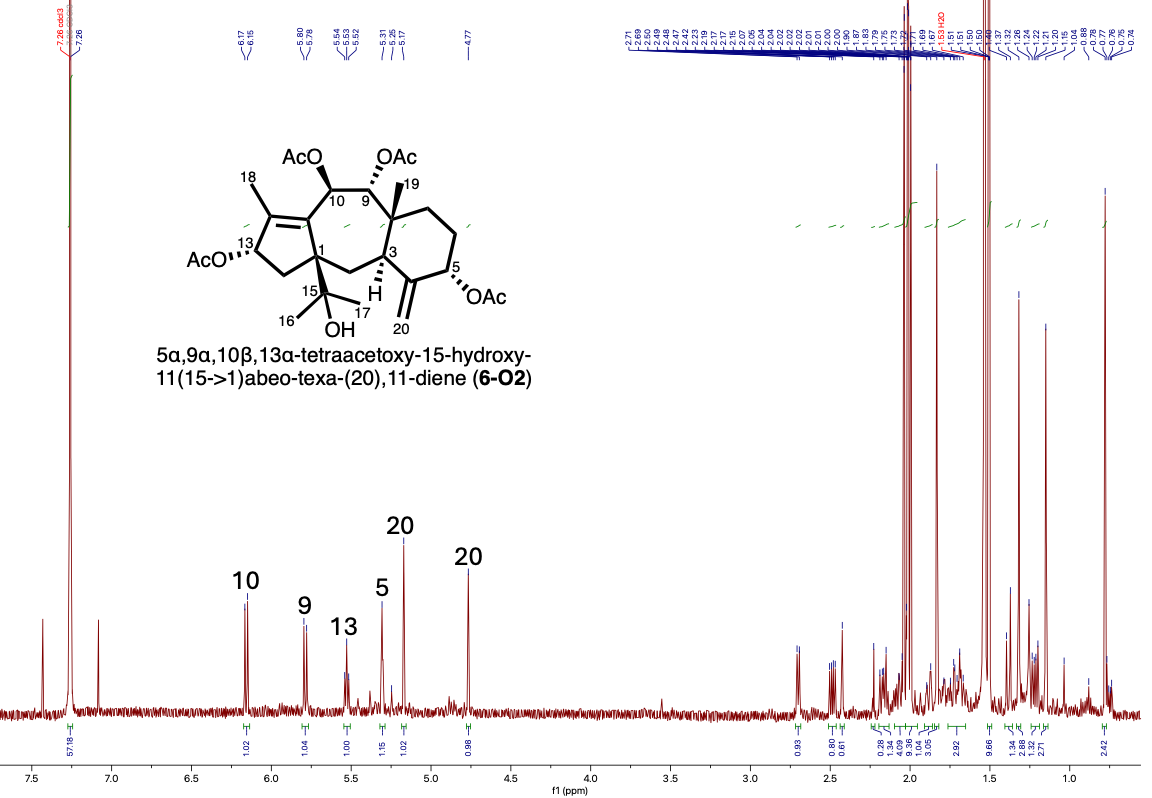
**

**Fig. S41. ^1^H-NMR spectrum of 15-hydroxy-11(15→1)*abeo*-taxusin (6-O2) (CDCl_3_, 600 ΜΗz, 298 K).**

**
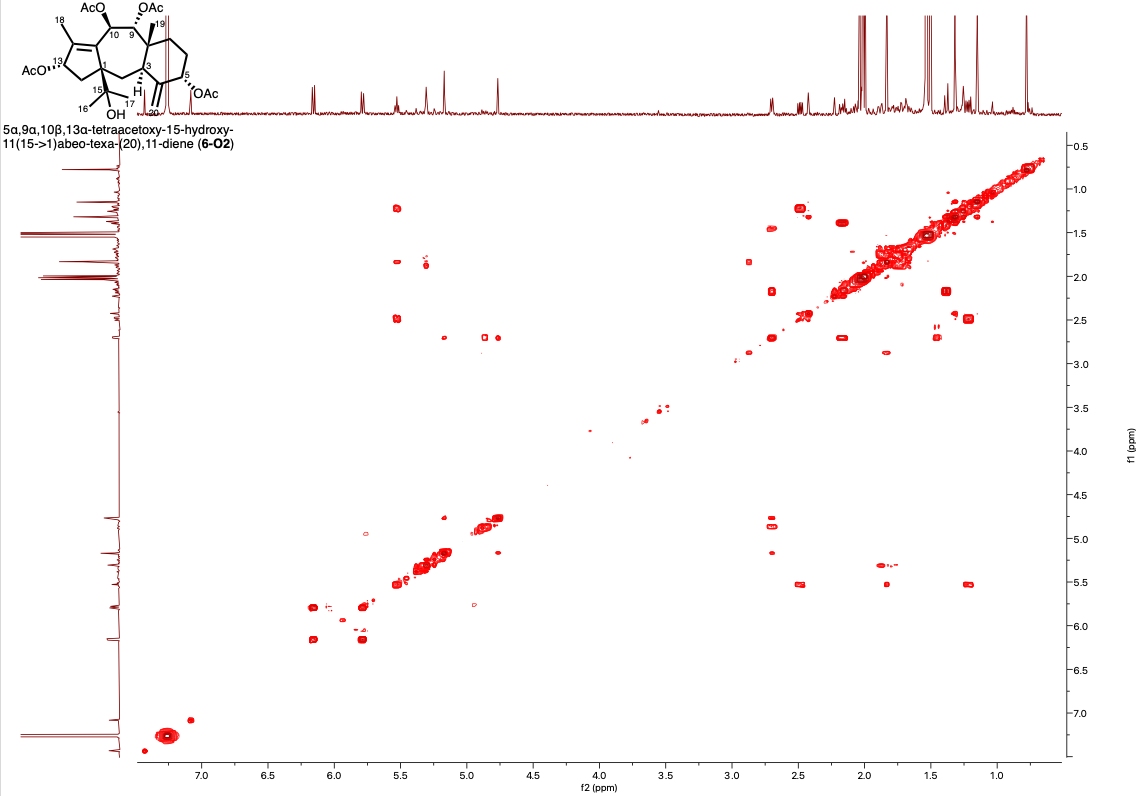
**

**Fig. S42. COSY spectrum of 15-hydroxy-11(15→1)*abeo*-taxusin (6-O2) (CDCl_3_, 600 ΜΗz, 298 K).**

**
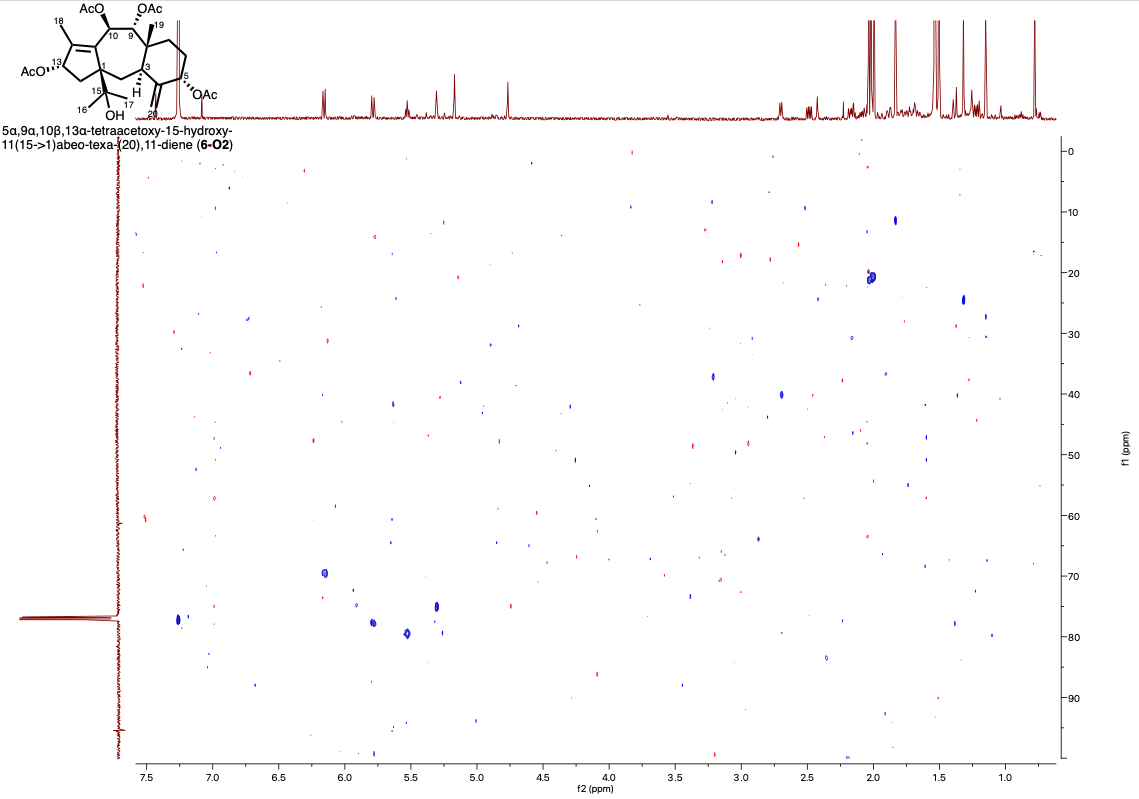
**

**Fig. S43. HSQC spectrum of 15-hydroxy-11(15→1)*abeo*-taxusin (6-O2) (CDCl_3_, 600 ΜΗz, 298 K).**

**
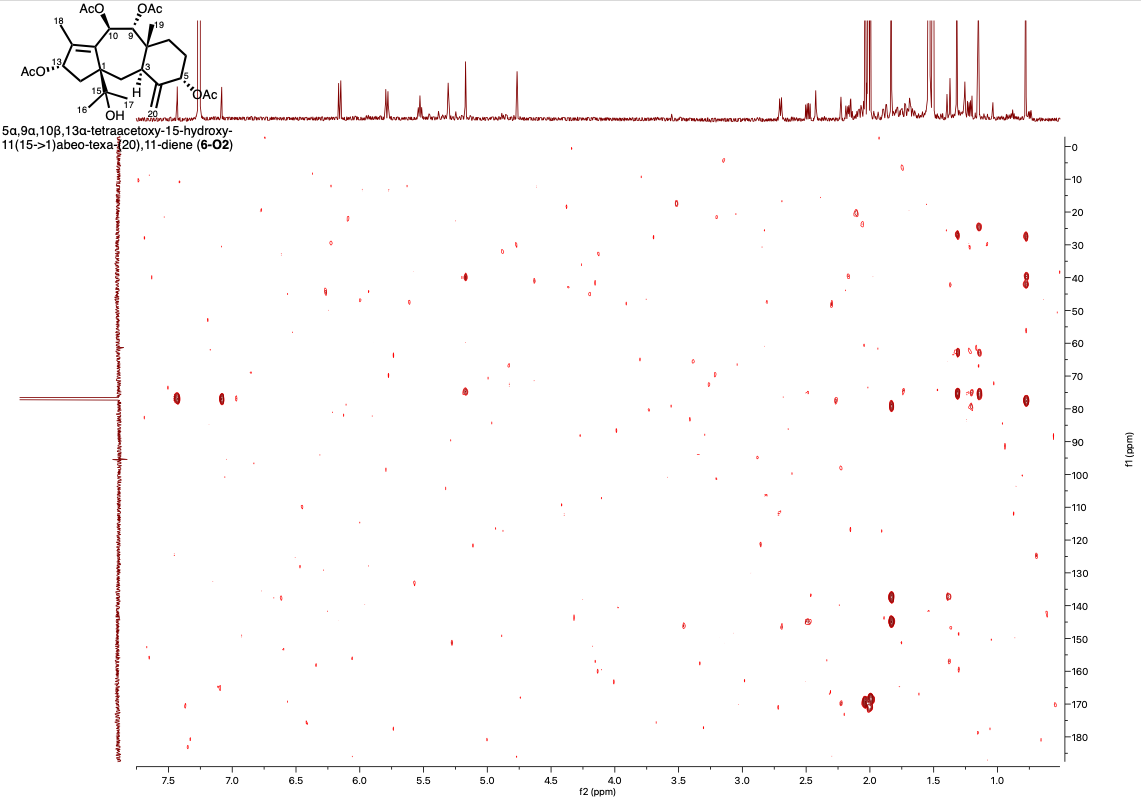
**

**Fig. S44. HMBC spectrum of 15-hydroxy-11(15→1)*abeo*-taxusin (6-O2) (CDCl_3_, 600 ΜΗz, 298 K).**

**
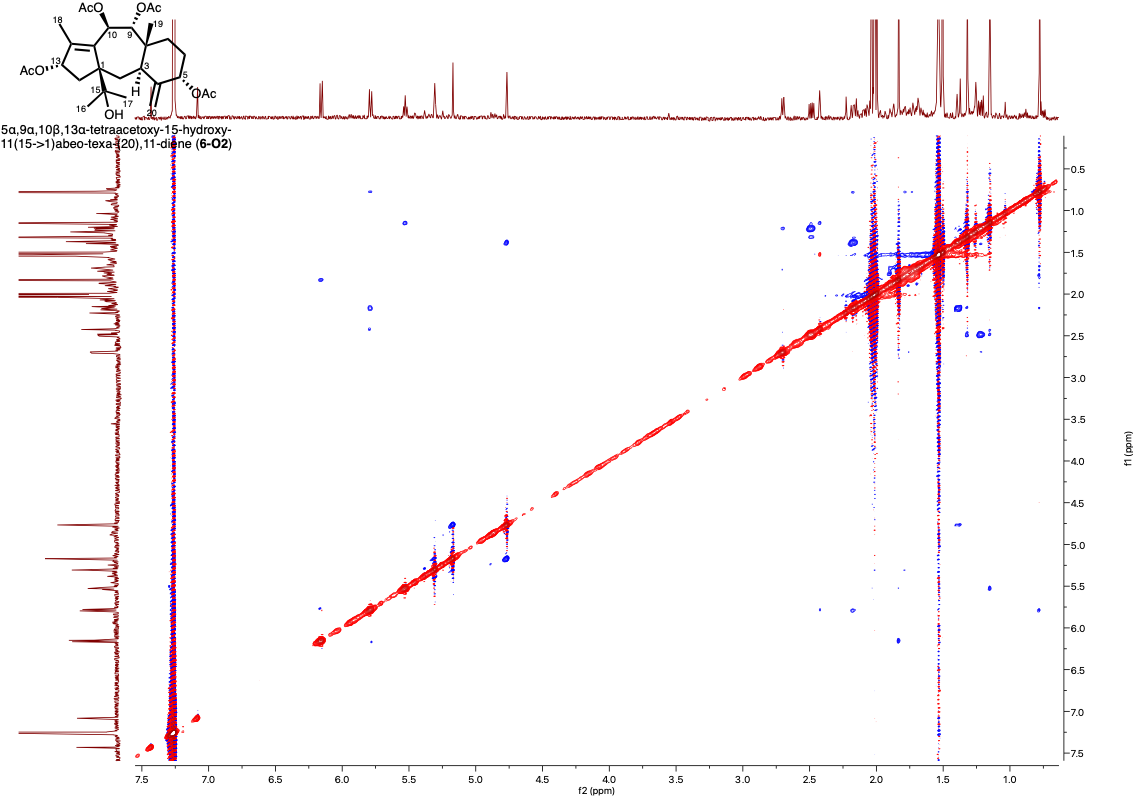
**

**Fig. S45. ROESY spectrum of 15-hydroxy-11(15→1)*abeo*-taxusin (6-O2) (CDCl_3_, 600 ΜΗz, 298 K).**

**
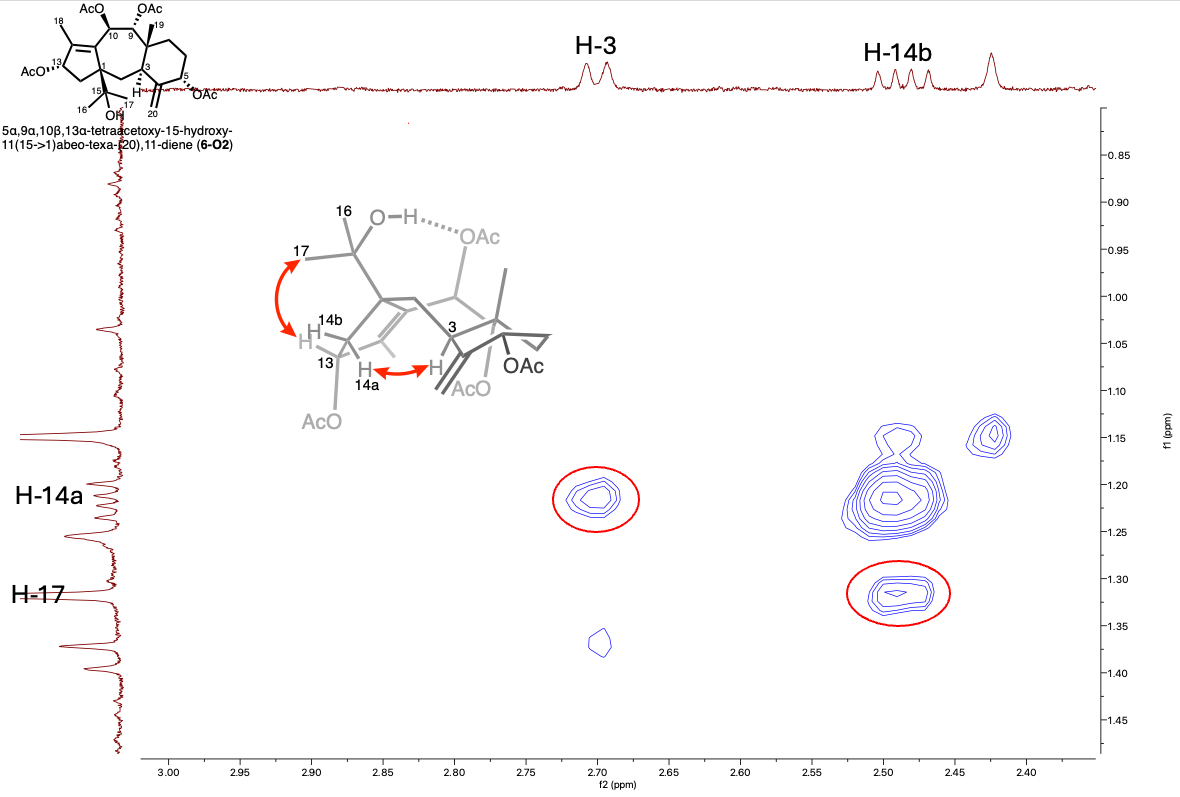
**

**Fig. S46. ROESY spectrum of 15-hydroxy-11(15→1)*abeo*-taxusin (6-O2) (CDCl_3_, 600 ΜΗz, 298 K) showing key ROESY correlations.**

**
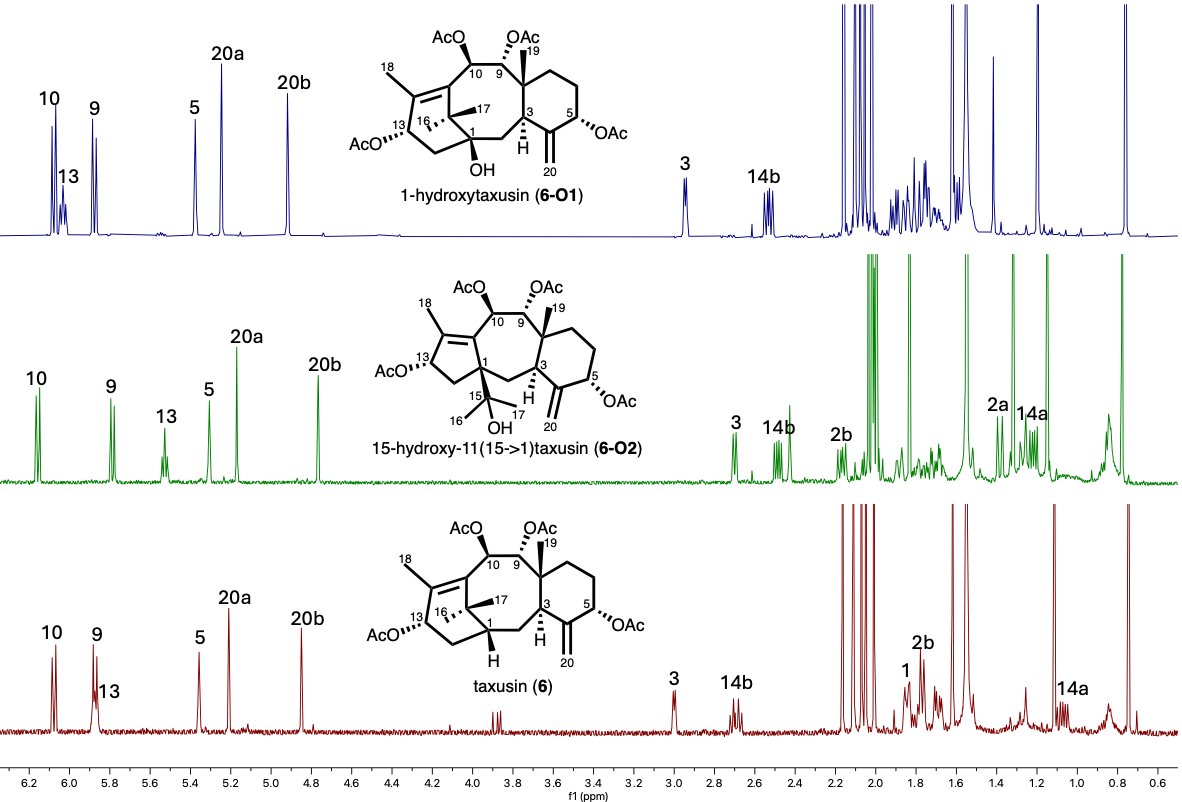
**

**Fig. S47. ^1^H-NMR spectra of taxusin (6), 1β-hydroxytaxusin (6-O1), and 15-hydroxy-11(15→1)*abeo*-taxusin (6-O2) in CDCl_3_.**


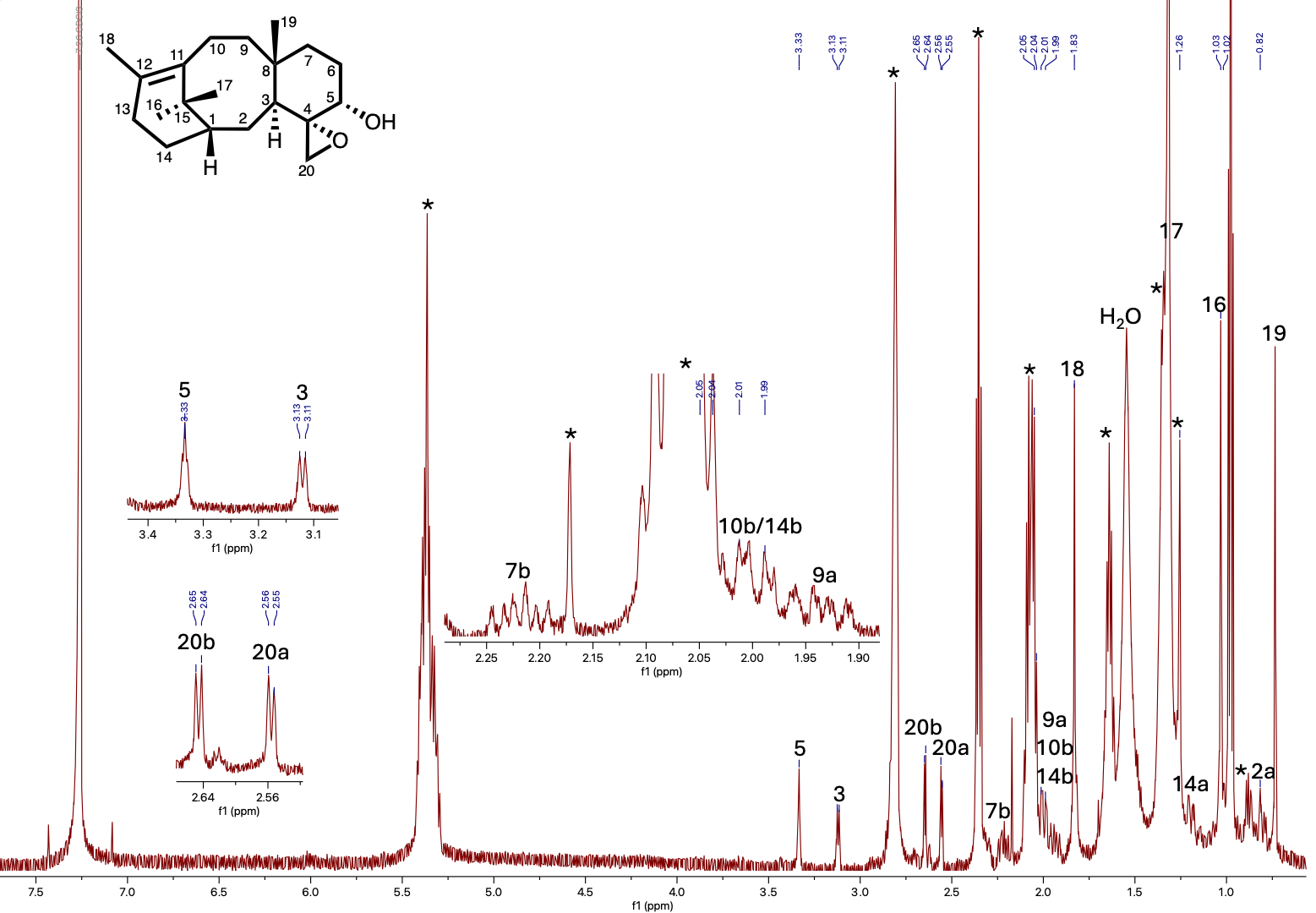


**Fig. S48. ^1^H-NMR spectrum of 4α,20-epoxy-taxadien-5α-ol (compound 2’d) (CDCl_3_, 600 ΜΗz, 298 K). Asterisk indicates co-eluting impurities.**

**
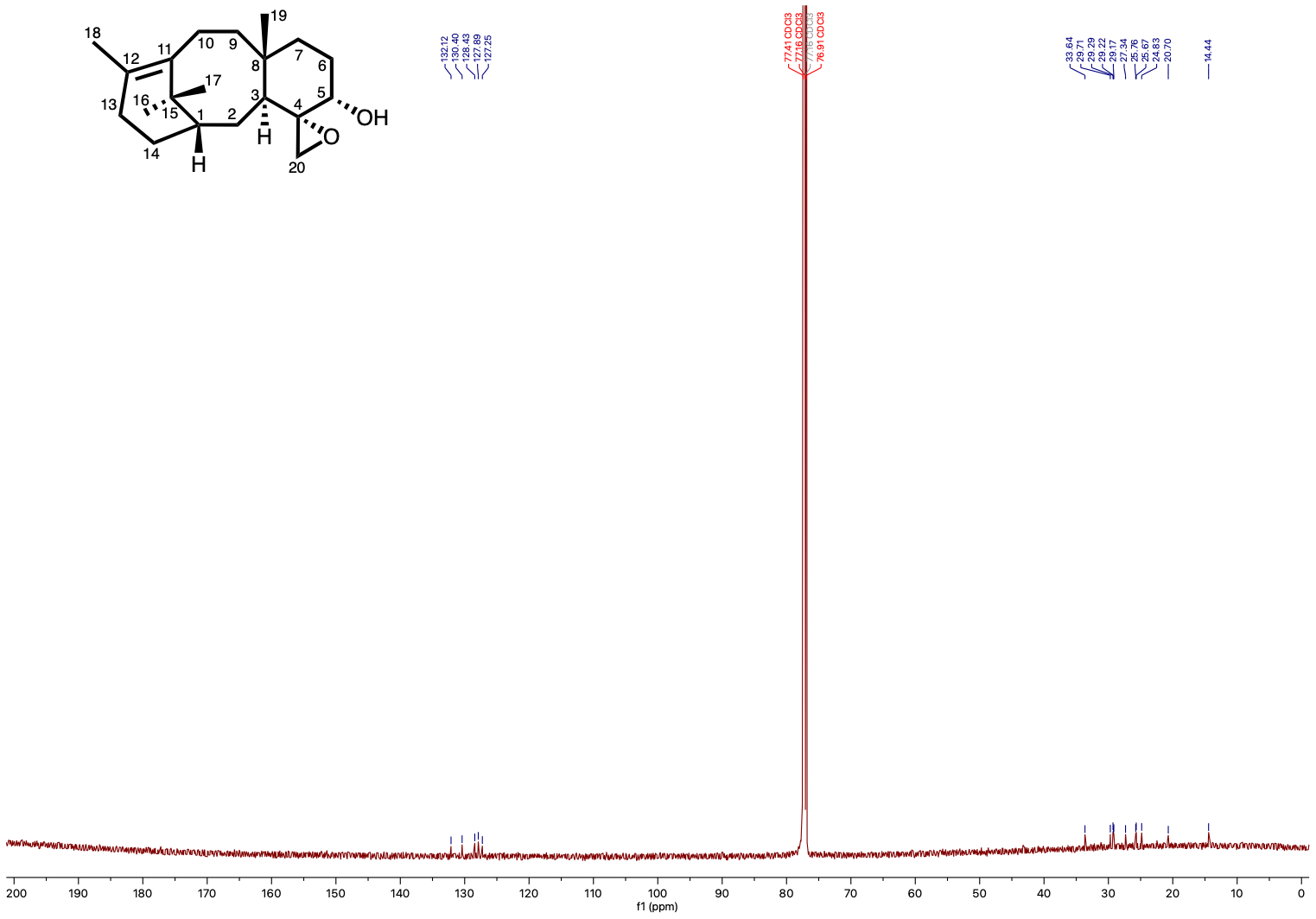
**

**Fig. S49. ^13^C-NMR spectrum of 4α,20-epoxy-taxadien-5α-ol (CDCl_3_, 500 ΜΗz, 298 K).**

**
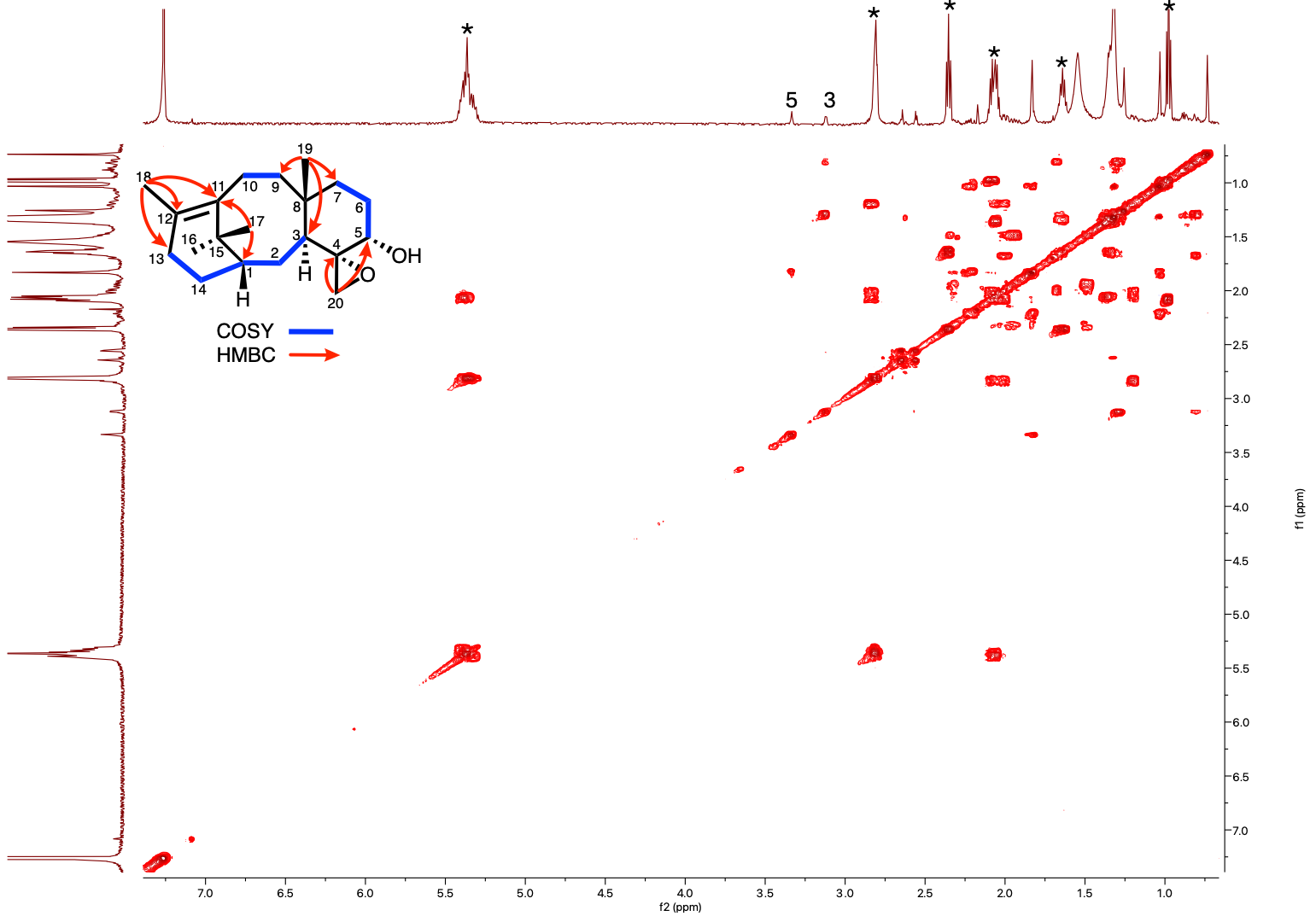
**

**Fig. S50. COSY spectrum of 4α,20-epoxy-taxadien-5α-ol (CDCl_3_, 600 ΜΗz, 298 K). Asterisk indicates co-eluting impurities.**

**
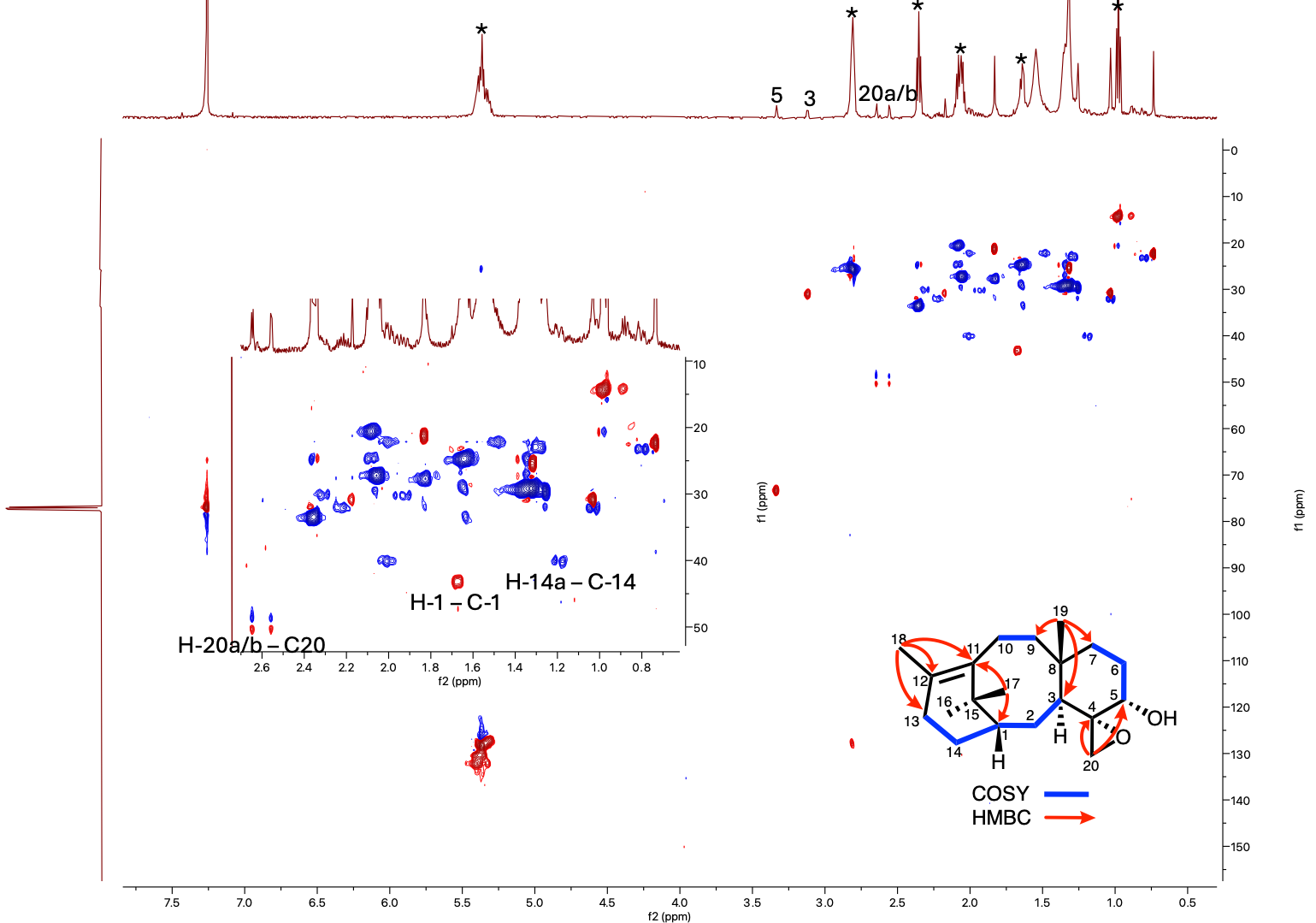
**

**Fig. S51. HSQC spectrum of 4α,20-epoxy-taxadien-5α-ol (CDCl_3_, 500 ΜΗz, 298 K). Asterisk indicates co-eluting impurities.**

**
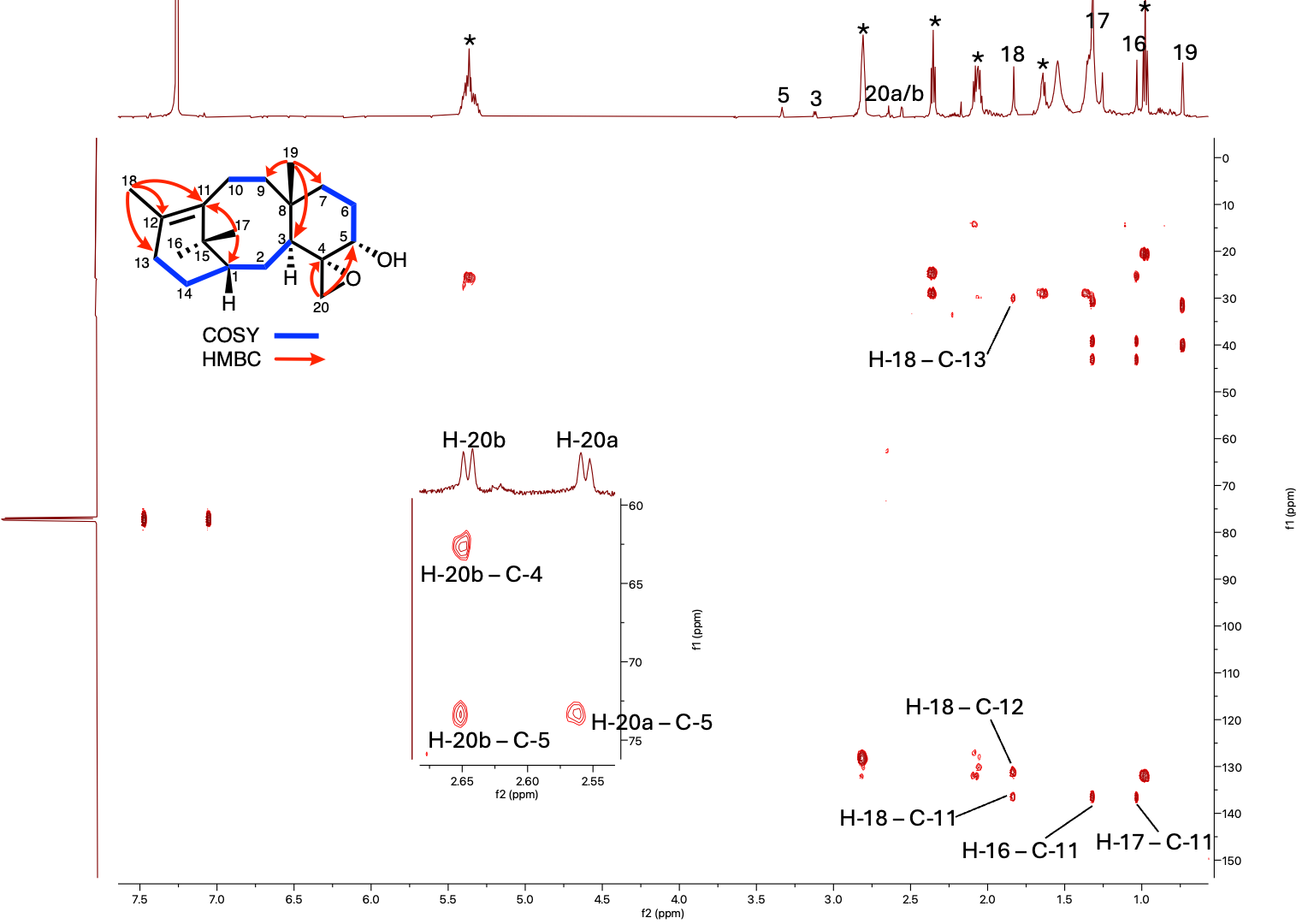
**

**Fig. S52. HMBC spectrum of 4α,20-epoxy-taxadien-5α-ol (CDCl_3_, 500 ΜΗz, 298 K). Asterisk indicates co-eluting impurities.**

**
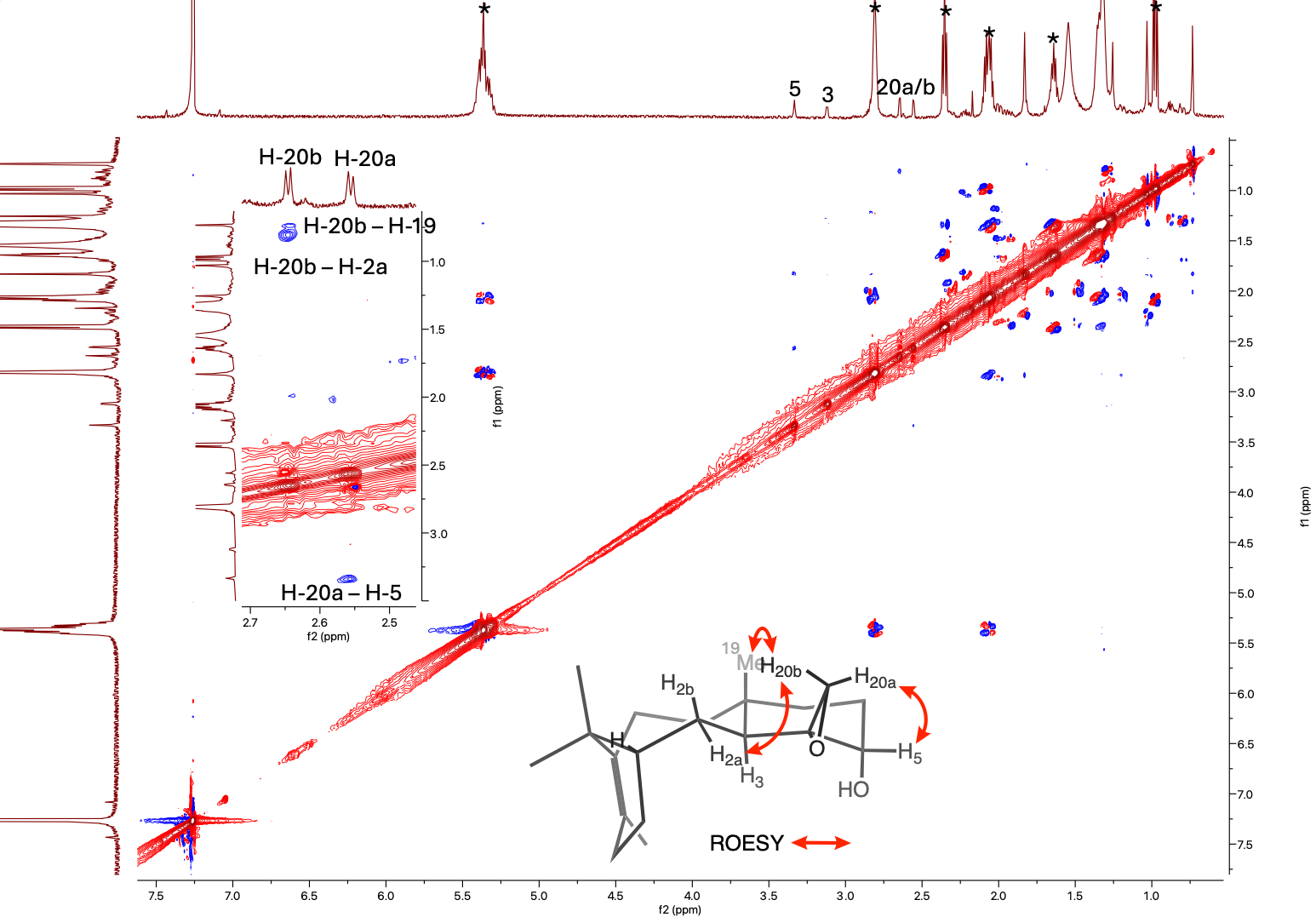
**

**Fig. S53. ROESY spectrum of 4α,20-epoxy-taxadien-5α-ol (CDCl_3_, 500 ΜΗz, 298 K). Asterisk indicates co-eluting impurities.**

**
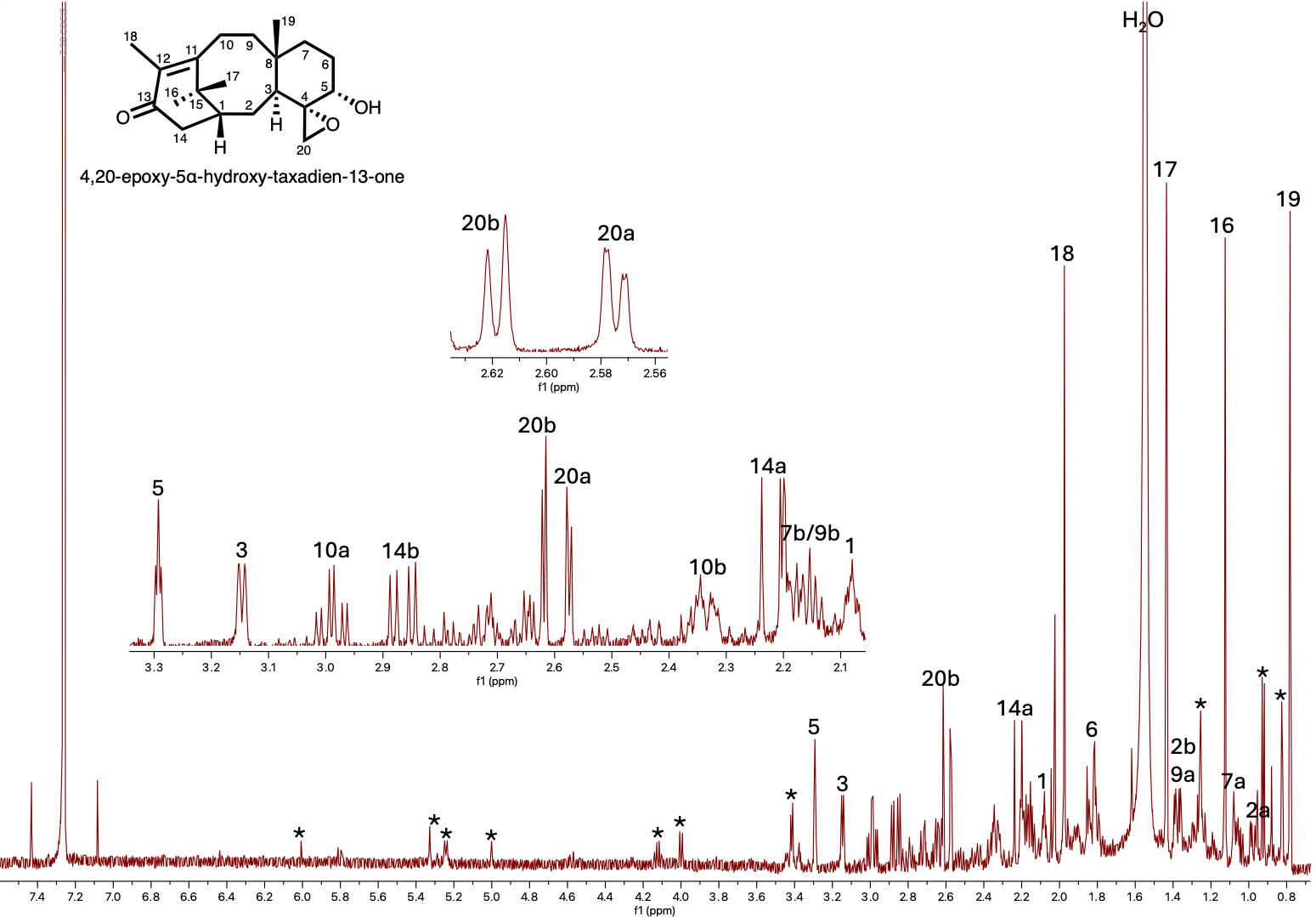
**

**Fig. S54. ^1^H-NMR spectrum of 4α,20-epoxy-5α-hydroxy-taxadien-13-one (CDCl_3_, 600 ΜΗz, 298 K). Asterisk indicates co-eluting impurities.**

**
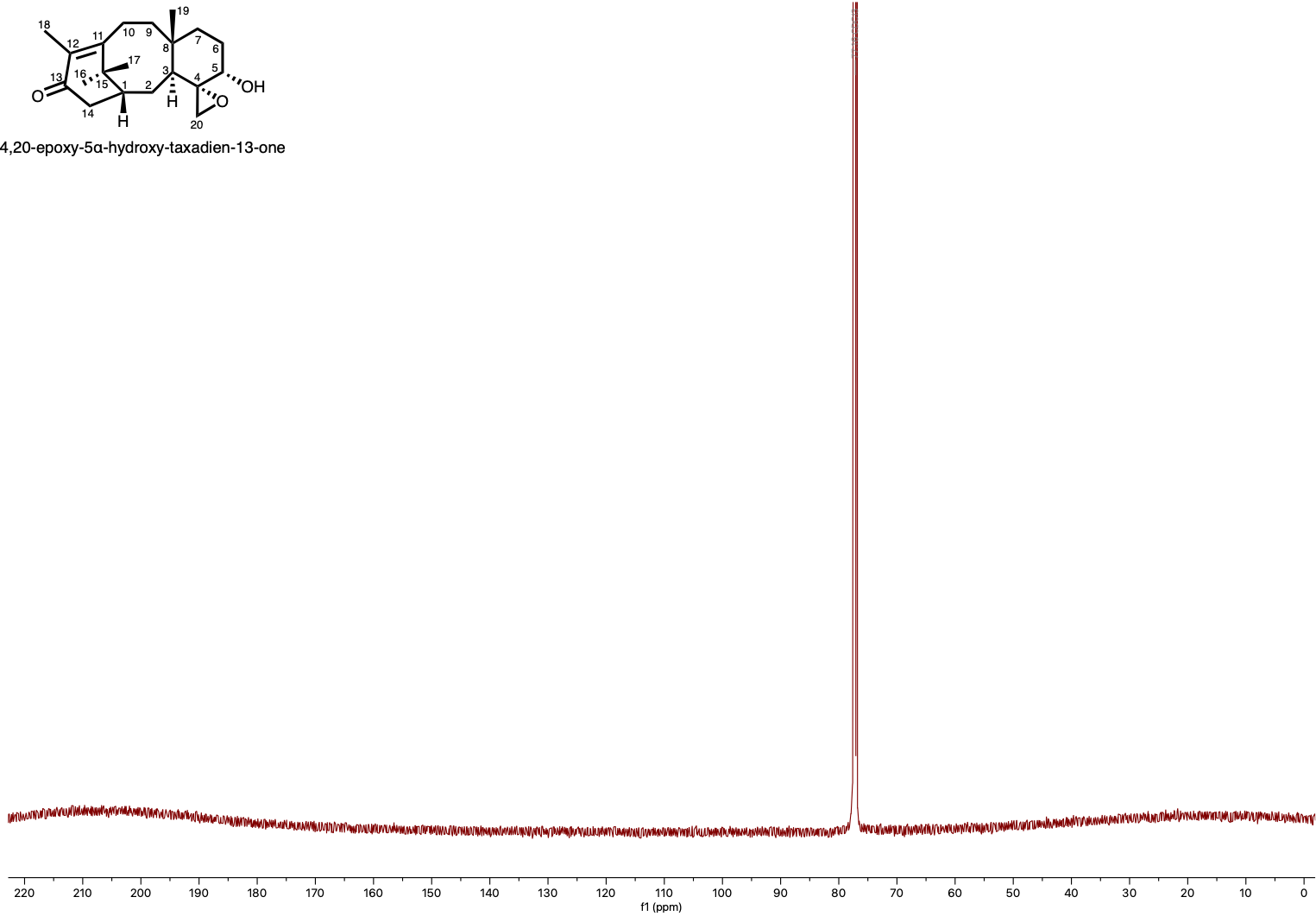
**

**Fig. S55. ^13^C-NMR spectrum of 4α,20-epoxy-5α-hydroxy-taxadien-13-one (CDCl_3_, 500 ΜΗz, 298 K).
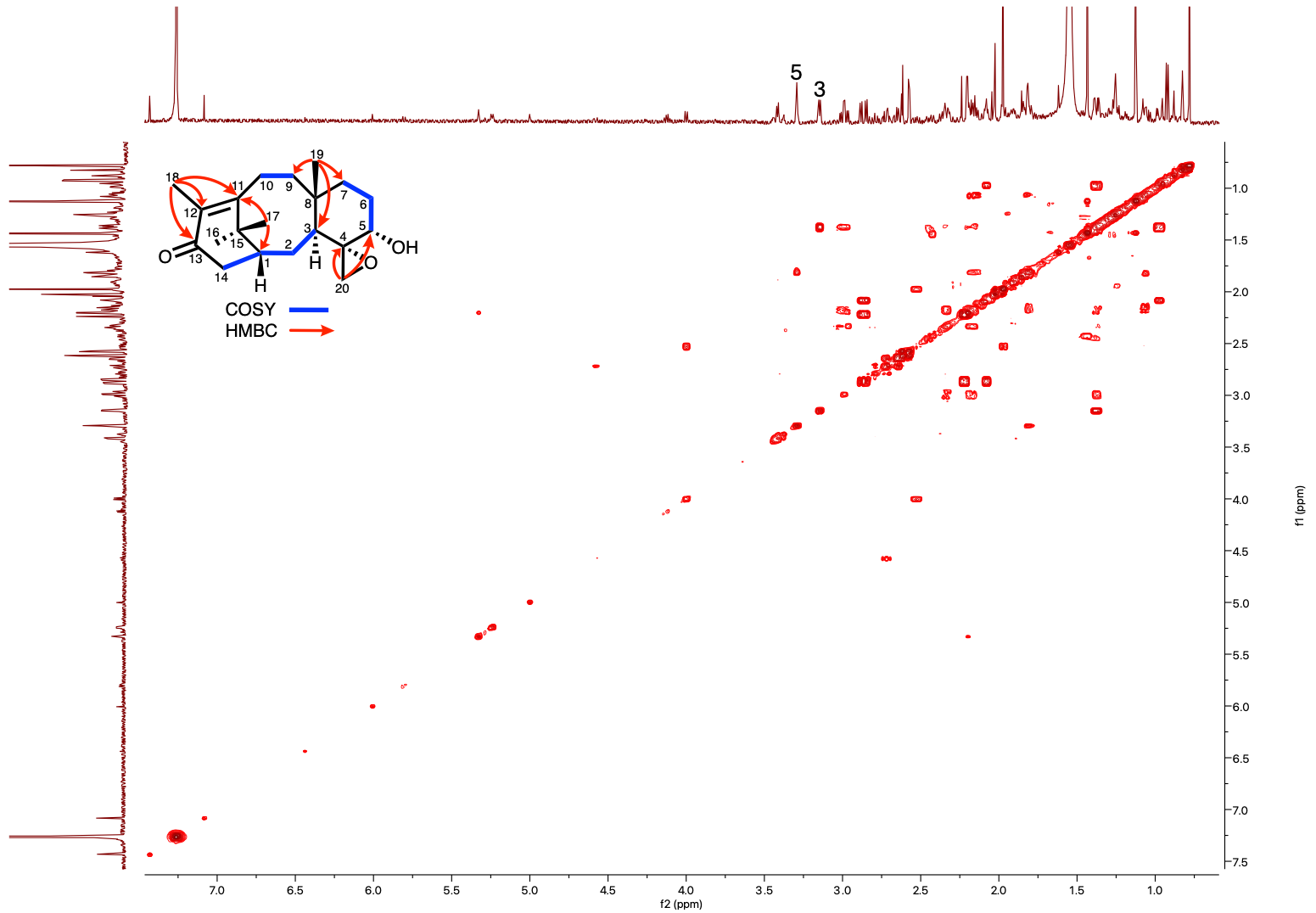
**

**Fig. S56. COSY spectrum of 4α,20-epoxy-5α-hydroxy-taxadien-13-one (CDCl_3_, 600 ΜΗz, 298 K).
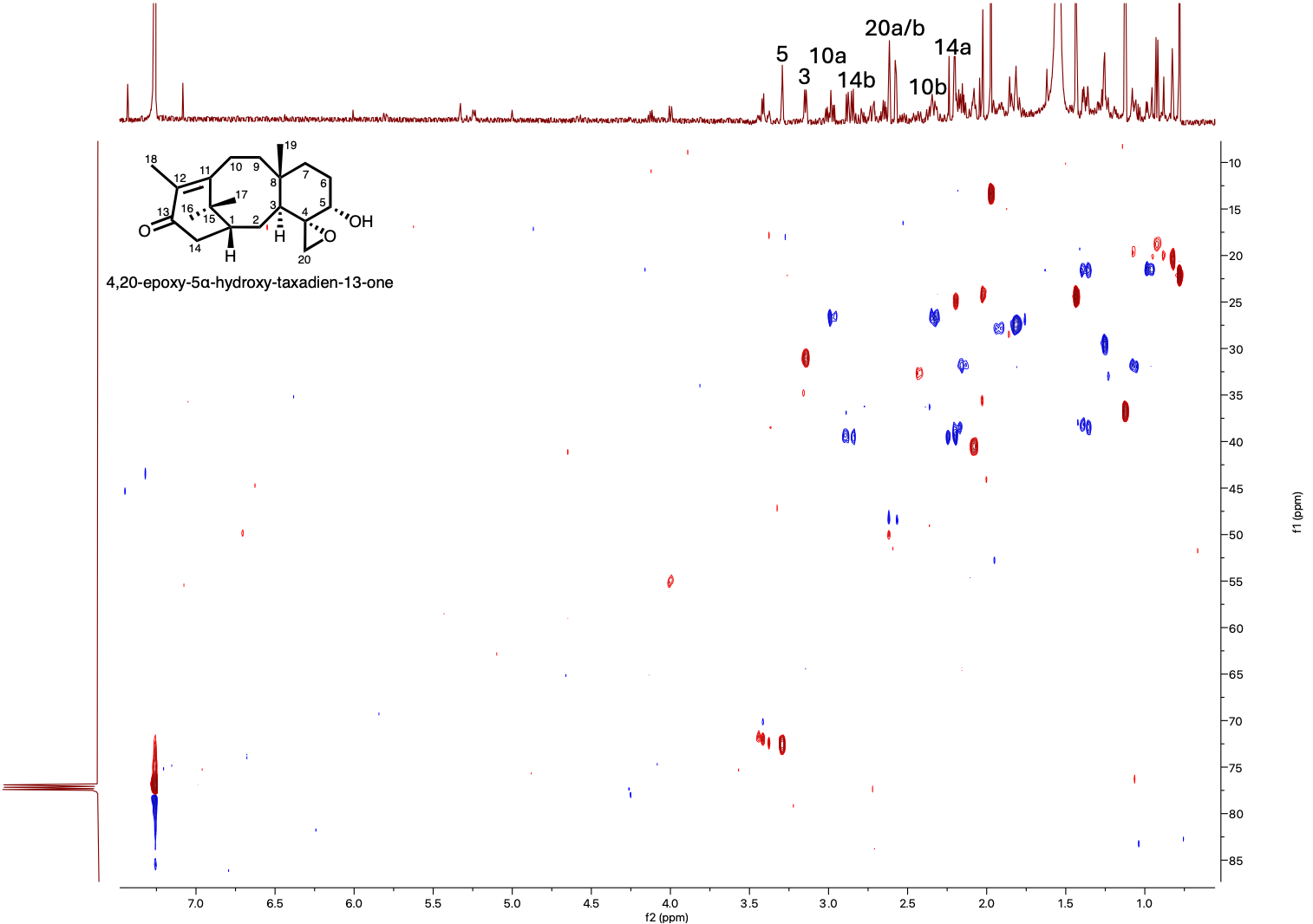
**

**Fig. S57. HSQC spectrum of 4α,20-epoxy-5α-hydroxy-taxadien-13-one (CDCl_3_, 500 ΜΗz, 298 K).**

**
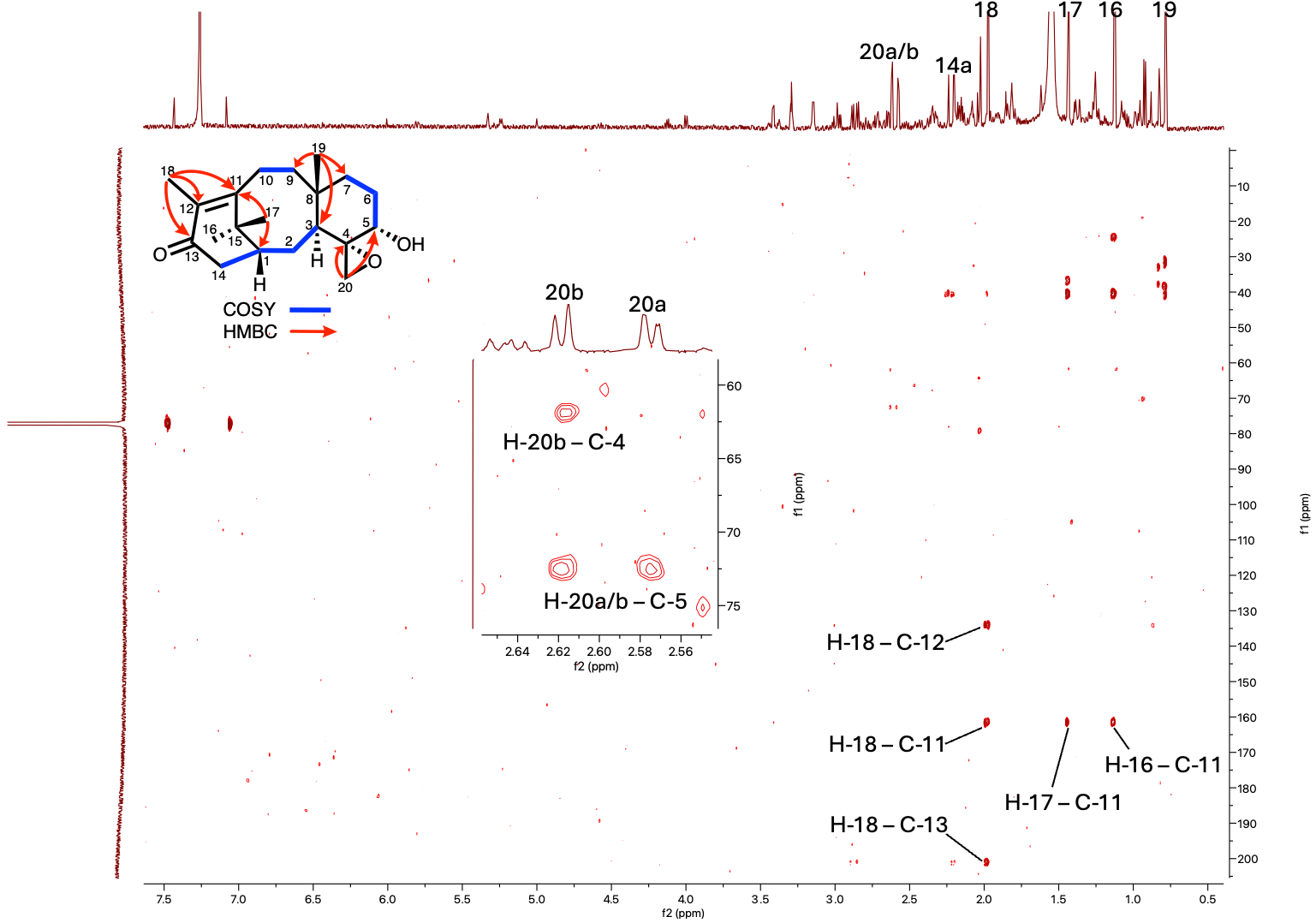
**

**Fig. S58. HMBC spectrum of 4α,20-epoxy-5α-hydroxy-taxadien-13-one (CDCl_3_, 500 ΜΗz, 298 K).**

**
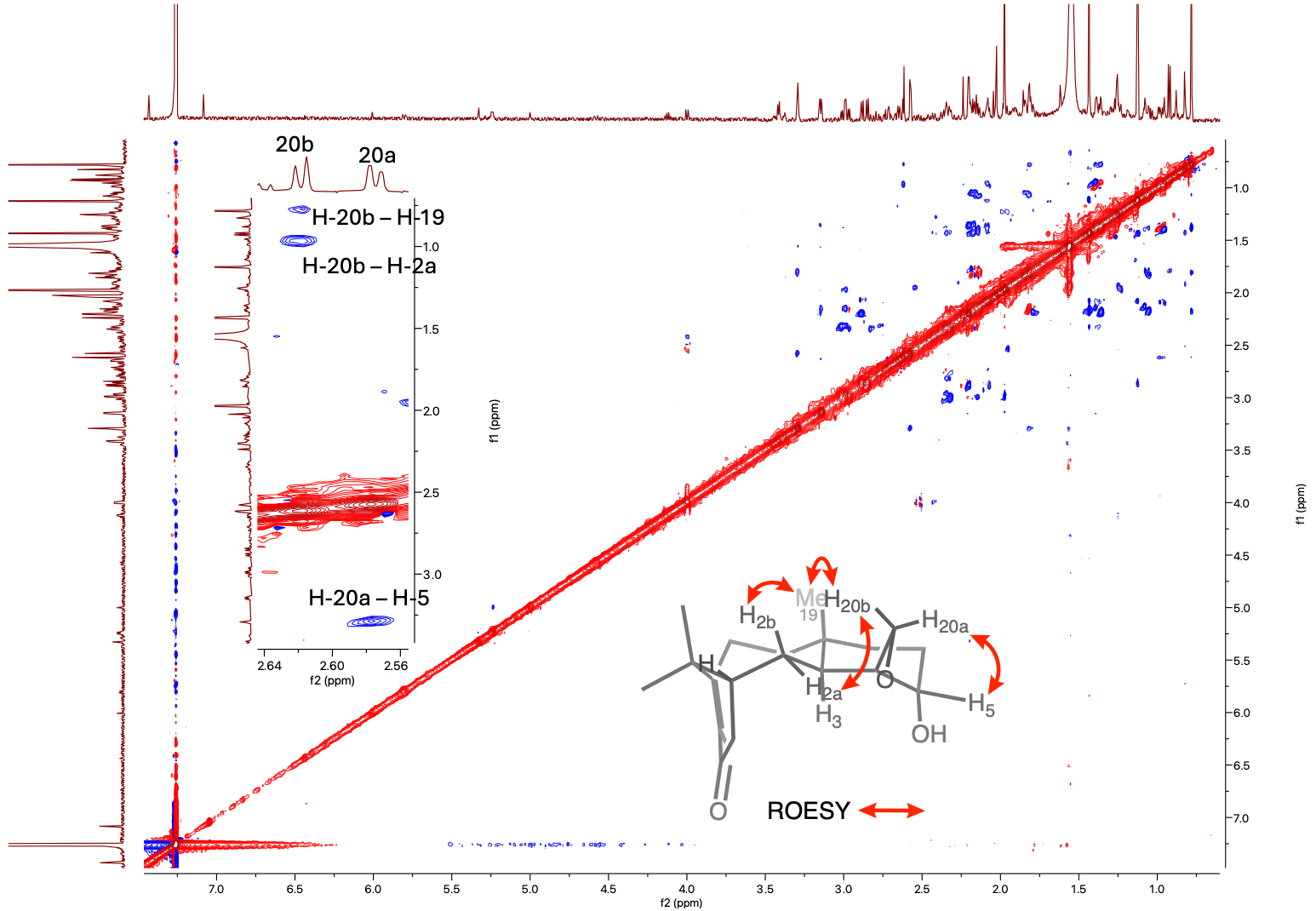
**

**Fig. S59. ROESY spectrum of 4α,20-epoxy-5α-hydroxy-taxadien-13-one (CDCl_3_, 500 ΜΗz, 298 K).**

**
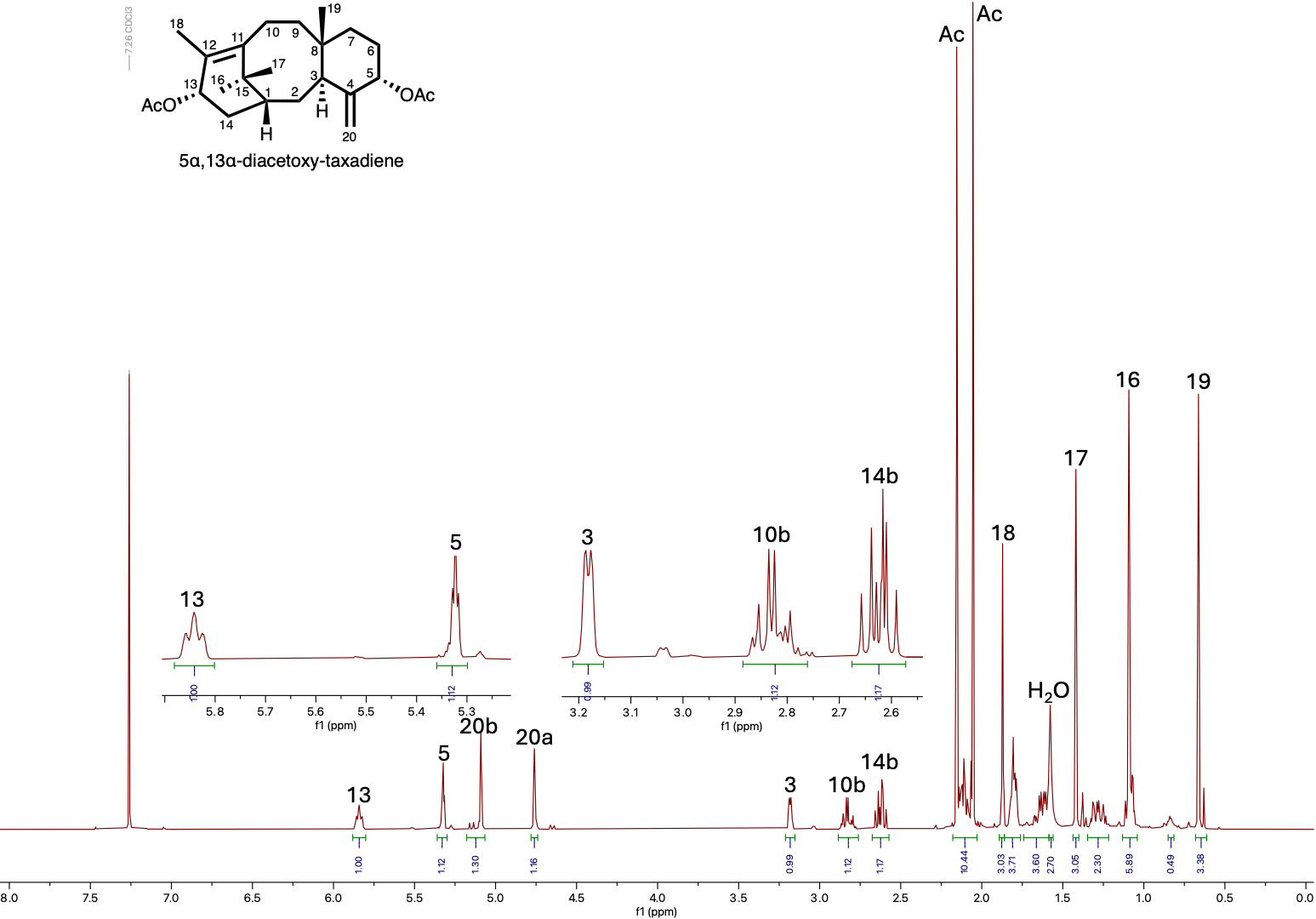
**

**Fig. S60. ^1^H-NMR spectrum of 5α,13α-diacetoxy-taxadiene (CDCl_3_, 500 ΜΗz, 298 K).**

**
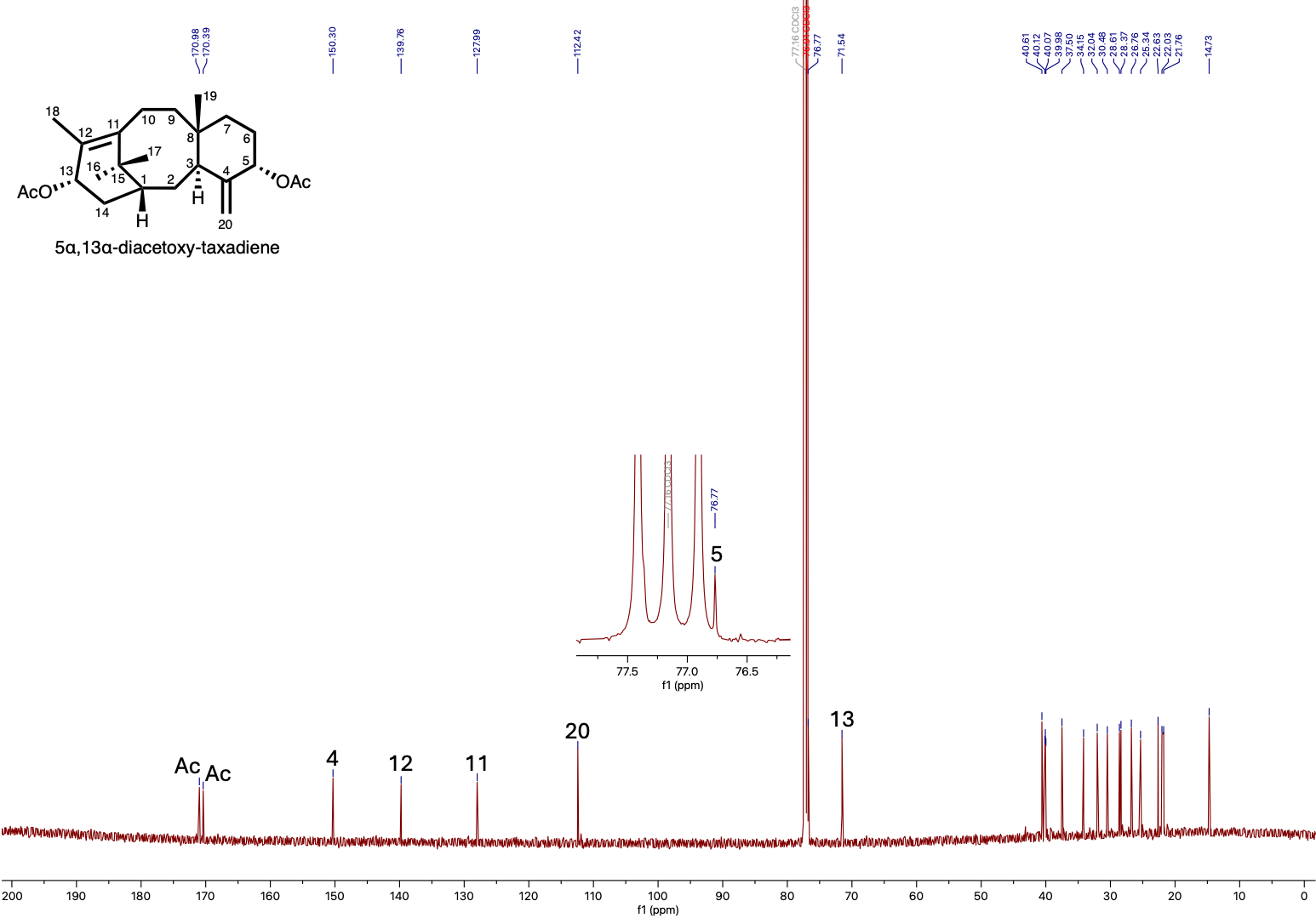
**

**Fig. S61. ^13^C-NMR spectrum of 5α,13α-diacetoxy-taxadiene (CDCl_3_, 500 ΜΗz, 298 K).**

**
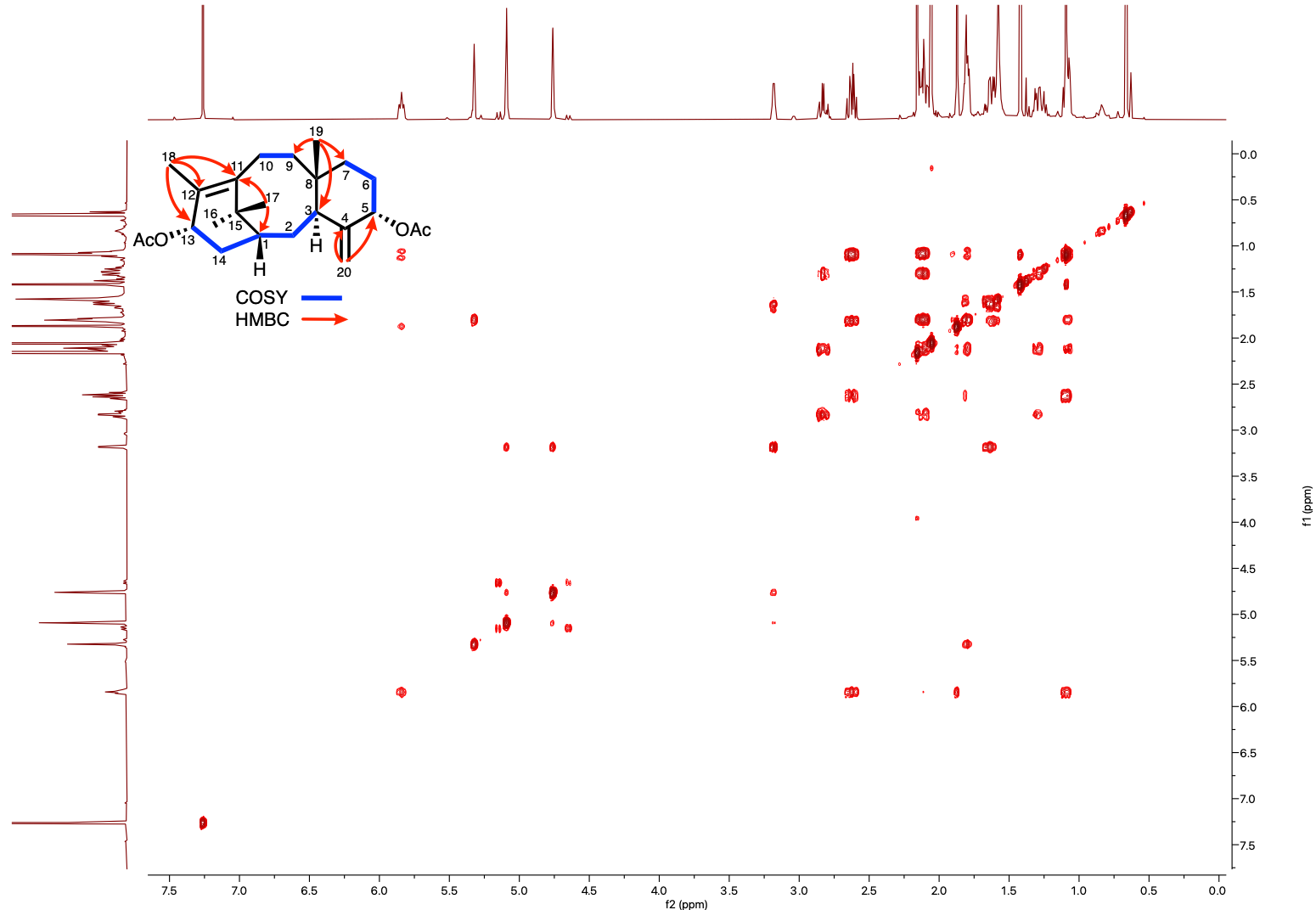
**

**Fig. S62. COSY spectrum of 5α,13α-diacetox-taxadiene (CDCl_3_, 500 ΜΗz, 298 K).**

**
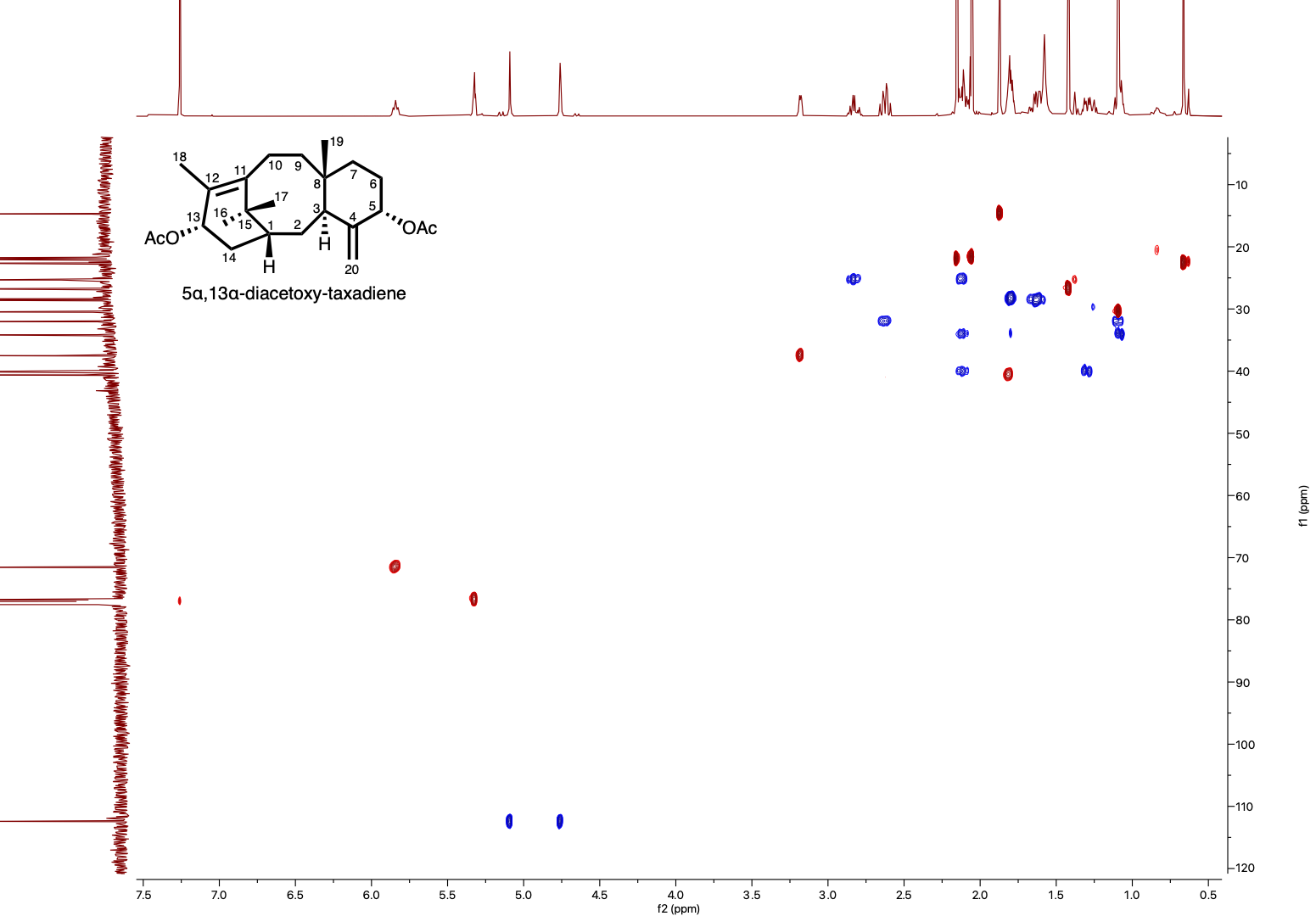
**

**Fig. S63. HSQC spectrum of 5α,13α-diacetoxy-taxadiene (CDCl_3_, 500 ΜΗz, 298 K).**

**
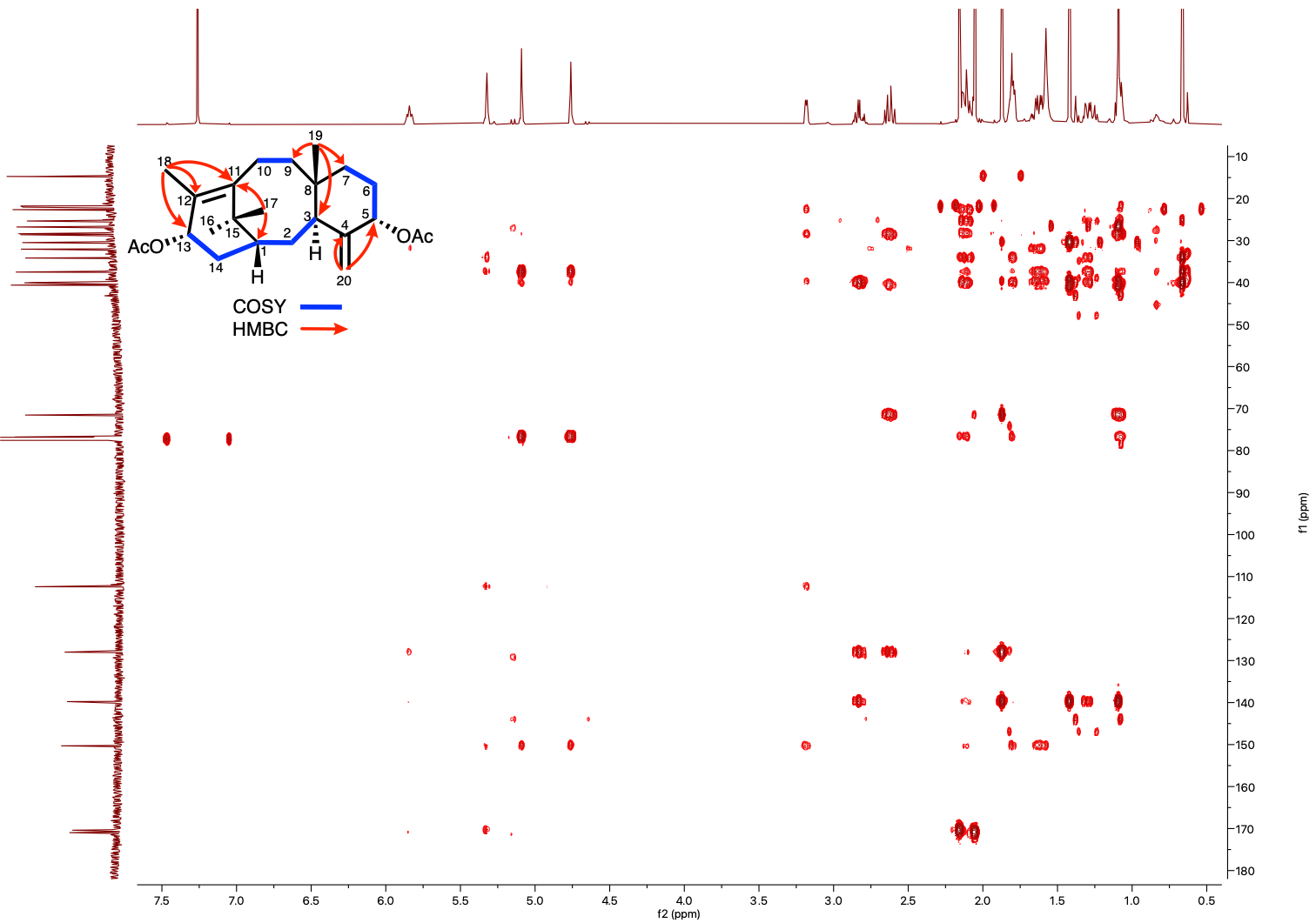
**

**Fig. S64. HMBC spectrum of 5α,13α-diacetoxy-taxadiene (CDCl_3_, 500 ΜΗz, 298 K).**

**
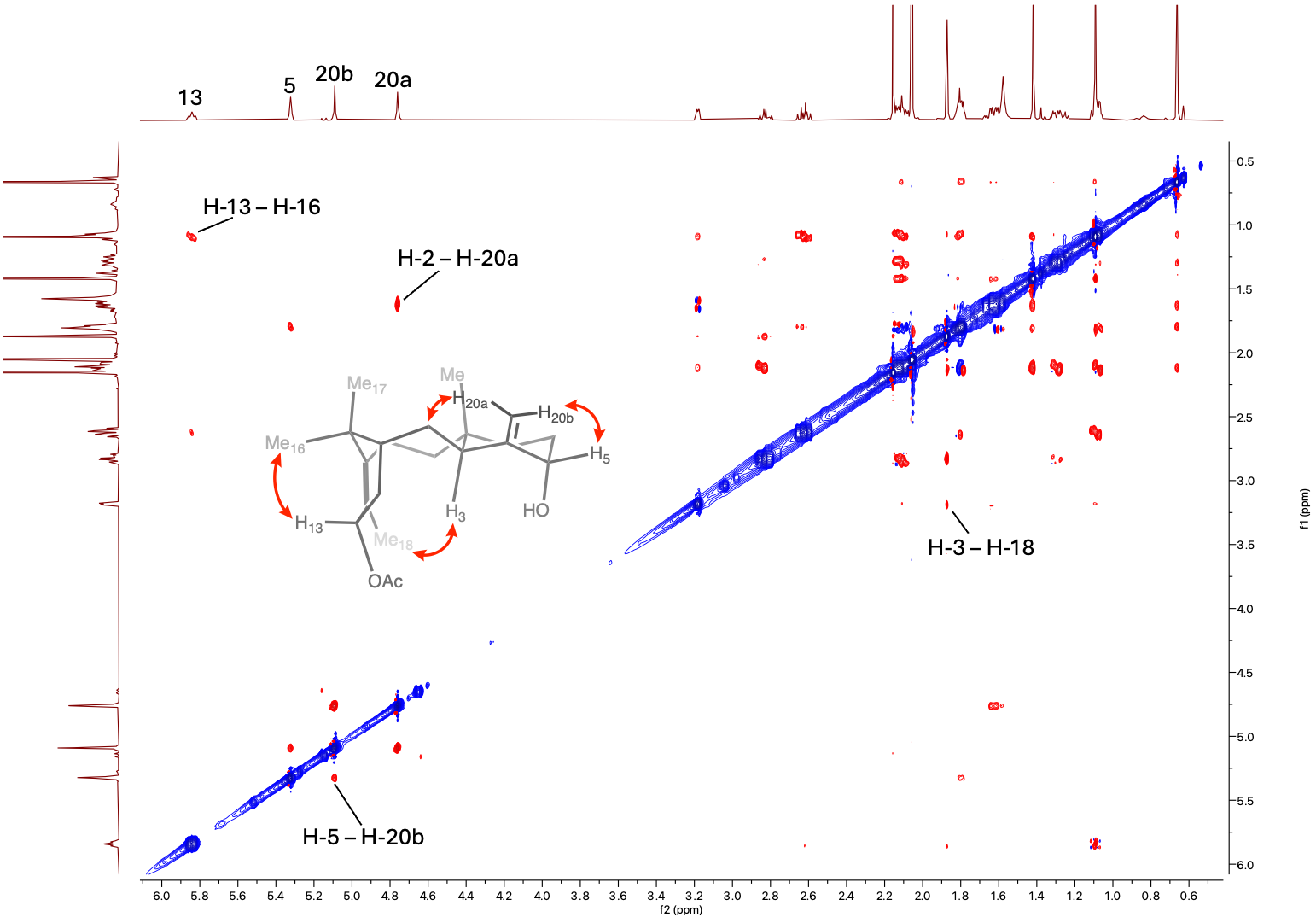
**

**Fig. S65. ROESY spectrum of 5α,13α-diacetoxy-taxadiene (CDCl_3_, 500 ΜΗz, 298 K).**

# References

1. [Liu, J. C.-T., De La Peña, R., Tocol, C. & Sattely, E. S. Reconstitution of early paclitaxel biosynthetic network. *Nat. Commun.* **15**, 1419 (2024).](http://paperpile.com/b/CAQ5G4/vB77)

2. [Chau, M., Walker, K., Long, R. & Croteau, R. Regioselectivity of taxoid-O-acetyltransferases: heterologous expression and characterization of a new taxadien-5alpha-ol-O-acetyltransferase. *Arch. Biochem. Biophys.* **430**, 237–246 (2004).](http://paperpile.com/b/CAQ5G4/z8vLh)

3. [Barton, N. A. *et al.* Accessing low-oxidation state taxanes: is taxadiene-4(5)-epoxide on the taxol biosynthetic pathway? *Chem. Sci.* **7**, 3102–3107 (2016).](http://paperpile.com/b/CAQ5G4/0Mifs)

4. [Edgar, S. *et al.* Mechanistic Insights into Taxadiene Epoxidation by Taxadiene-5α-Hydroxylase. *ACS Chem. Biol.* **11**, 460–469 (2016).](http://paperpile.com/b/CAQ5G4/popoc)

5. [Jennewein, S., Long, R. M., Williams, R. M. & Croteau, R. Cytochrome p450 taxadiene 5alpha-hydroxylase, a mechanistically unusual monooxygenase catalyzing the first oxygenation step of taxol biosynthesis. *Chem. Biol.* **11**, 379–387 (2004).](http://paperpile.com/b/CAQ5G4/mYWpv)

6. [Wang, Y.-F. *et al.* Natural taxanes: developments since 1828. *Chem. Rev.* **111**, 7652–7709 (2011).](http://paperpile.com/b/CAQ5G4/9Jibm)

7. [Zhao, Y. *et al.* Oxetane Ring Formation in Taxol Biosynthesis Is Catalyzed by a Bifunctional Cytochrome P450 Enzyme. *J Am Chem Soc* **146**, 801–810 (2024).](http://paperpile.com/b/CAQ5G4/zExWv)

8. [Kennedy, D. *et al.* Characterization of G3BPs: tissue specific expression, chromosomal localisation and rasGAP(120) binding studies. *J. Cell. Biochem.* **84**, 173–187 (2001).](http://paperpile.com/b/CAQ5G4/oJLCS)

9. [Reuper, H., Amari, K. & Krenz, B. Analyzing the G3BP-like gene family of Arabidopsis thaliana in early turnip mosaic virus infection. *Sci. Rep.* **11**, 2187 (2021).](http://paperpile.com/b/CAQ5G4/KCC0a)

10. [Zhao, Q., Leung, S., Corbett, A. H. & Meier, I. Identification and characterization of the Arabidopsis orthologs of nuclear transport factor 2, the nuclear import factor of ran. *Plant Physiol.* **140**, 869–878 (2006).](http://paperpile.com/b/CAQ5G4/2j1II)

11. [Ha, N. C., Choi, G., Choi, K. Y. & Oh, B. H. Structure and enzymology of Delta5-3-ketosteroid isomerase. *Curr. Opin. Struct. Biol.* **11**, 674–678 (2001).](http://paperpile.com/b/CAQ5G4/X0YD8)

12. [Mori, T. *et al.* Molecular basis for the unusual ring reconstruction in fungal meroterpenoid biogenesis. *Nat. Chem. Biol.* **13**, 1066–1073 (2017).](http://paperpile.com/b/CAQ5G4/Zl1eg)

13. [Yeh, A. H.-W. *et al.* De novo design of luciferases using deep learning. *Nature* **614**, 774–780 (2023).](http://paperpile.com/b/CAQ5G4/ioaFb)

14. [Yu, S., Zhu, Y.-X., Peng, C. & Li, J. Two new sterol derivatives isolated from the endophytic fungus Aspergillus tubingensis YP-2. *Nat. Prod. Res.* **35**, 3277–3284 (2021).](http://paperpile.com/b/CAQ5G4/dK2nc)

15. [Williams, D. C. *et al.* Heterologous expression and characterization of a ‘Pseudomature’ form of taxadiene synthase involved in paclitaxel (Taxol) biosynthesis and evaluation of a potential intermediate and inhibitors of the multistep diterpene cyclization reaction. *Arch Biochem Biophys* **379**, 137–146 (2000).](http://paperpile.com/b/CAQ5G4/QYnjU)

16. [Walker, K., Schoendorf, A. & Croteau, R. Molecular cloning of a taxa-4(20),11(12)-dien-5alpha-ol-O-acetyl transferase cDNA from Taxus and functional expression in Escherichia coli. *Arch Biochem Biophys* **374**, 371–380 (2000).](http://paperpile.com/b/CAQ5G4/yqB7Y)

17. [Schoendorf, A., Rithner, C. D., Williams, R. M. & Croteau, R. B. Molecular cloning of a cytochrome P450 taxane 10β-hydroxylase cDNA from Taxus and functional expression in yeast. *Proceedings of the National Academy of Sciences* **98**, 1501–1506 (2001).](http://paperpile.com/b/CAQ5G4/34e0G)

18. [Walker, K. & Croteau, R. Molecular cloning of a 10-deacetylbaccatin III-10-O-acetyl transferase cDNA from Taxus and functional expression in Escherichia coli. *Proc Natl Acad Sci U S A* **97**, 583–587 (2000).](http://paperpile.com/b/CAQ5G4/1dRiL)

19. [Jennewein, S., Rithner, C. D., Williams, R. M. & Croteau, R. B. Taxol biosynthesis: taxane 13 alpha-hydroxylase is a cytochrome P450-dependent monooxygenase. *Proc Natl Acad Sci U S A* **98**, 13595–13600 (2001).](http://paperpile.com/b/CAQ5G4/iBtLH)

20. [Jiang, B. *et al.* Characterization and heterologous reconstitution of biosynthetic enzymes leading to baccatin III. *Science* eadj3484 (2024).](http://paperpile.com/b/CAQ5G4/lO8He)

21. [Yang, C. *et al.* Biosynthesis of the highly oxygenated tetracyclic core skeleton of Taxol. *Nat Commun* **15**, 2339 (2024).](http://paperpile.com/b/CAQ5G4/q4Pth)

22. [Synthetic biology identifies the minimal gene set required for paclitaxel biosynthesis in a plant chassis. *Mol. Plant* **16**, 1951–1961 (2023).](http://paperpile.com/b/CAQ5G4/iWLLP)

23. [Chau, M. & Croteau, R. Molecular cloning and characterization of a cytochrome P450 taxoid 2alpha-hydroxylase involved in Taxol biosynthesis. *Arch. Biochem. Biophys.* **427**, 48–57 (2004).](http://paperpile.com/b/CAQ5G4/15i1K)

24. [Walker, K. & Croteau, R. Taxol biosynthesis: molecular cloning of a benzoyl-CoA:taxane 2alpha-O-benzoyltransferase cDNA from taxus and functional expression in Escherichia coli. *Proc. Natl. Acad. Sci. U. S. A.* **97**, 13591–13596 (2000).](http://paperpile.com/b/CAQ5G4/mAMPZ)

25. [Chau, M., Jennewein, S., Walker, K. & Croteau, R. Taxol biosynthesis: Molecular cloning and characterization of a cytochrome P450 taxoid 7 beta-hydroxylase. *Chem. Biol.* **11**, 663–672 (2004).](http://paperpile.com/b/CAQ5G4/bXLlt)

26. [Li, C. *et al.* A Cytochrome P450 Enzyme Catalyses Oxetane Ring Formation in Paclitaxel Biosynthesis. *Angew. Chem. Int. Ed Engl.* **63**, e202407070 (2024).](http://paperpile.com/b/CAQ5G4/ayjUj)

27. [Walker, K. D., Klettke, K., Akiyama, T. & Croteau, R. Cloning, heterologous expression, and characterization of a phenylalanine aminomutase involved in Taxol biosynthesis. *J Biol Chem* **279**, 53947–53954 (2004).](http://paperpile.com/b/CAQ5G4/02l0J)

28. [Walker, K., Fujisaki, S., Long, R. & Croteau, R. Molecular cloning and heterologous expression of the C-13 phenylpropanoid side chain-CoA acyltransferase that functions in Taxol biosynthesis. *Proc Natl Acad Sci U S A* **99**, 12715–12720 (2002).](http://paperpile.com/b/CAQ5G4/801p0)

29. [Sanchez-Muñoz, R. *et al.* A Novel Hydroxylation Step in the Taxane Biosynthetic Pathway: A New Approach to Paclitaxel Production by Synthetic Biology. *Front Bioeng Biotechnol* **8**, 410 (2020).](http://paperpile.com/b/CAQ5G4/ntays)

30. [Walker, K., Long, R. & Croteau, R. The final acylation step in taxol biosynthesis: cloning of the taxoid C13-side-chain N-benzoyltransferase from Taxus. *Proc. Natl. Acad. Sci. U. S. A.* **99**, 9166–9171 (2002).](http://paperpile.com/b/CAQ5G4/AU87M)

31. ReHman, H.-U., Rehman, A.-U., Choudhary, M.I. Structure Determination of New Texanes from Taxus wallichiana Zucc. *Jour. Chem. Soc. Pak.* **26**, 3, 302-307 (2004).

32. [Kotliar, D. *et al.* Identifying gene expression programs of cell-type identity and cellular activity with single-cell RNA-Seq. *Elife* **8**, (2019).](http://paperpile.com/b/CAQ5G4/XrOuW)

33. [Price, M. N., Dehal, P. S. & Arkin, A. P. FastTree 2--approximately maximum-likelihood trees for large alignments. *PLoS One* **5**, e9490 (2010).](http://paperpile.com/b/CAQ5G4/lWaeo)

34. [Al-Hilfi, A., Li, Z., Merz, K. M., Nawarathne, I. N. & Walker, K. D. Biocatalytic and Regioselective Exchange of 2-O-Benzoyl for 2-O-(m-Substituted)Benzoyl Groups to Make Precursors of Next-Generation Paclitaxel Drugs. *ChemCatChem* **16**, e202400186 (2024).](http://paperpile.com/b/CAQ5G4/ap2Fh)

35. [Long, R. M. & Croteau, R. Preliminary assessment of the C13-side chain 2’-hydroxylase involved in taxol biosynthesis. *Biochem. Biophys. Res. Commun.* **338**, 410–417 (2005).](http://paperpile.com/b/CAQ5G4/gOhBC)
