# Supplementary material for: Discovery of FoTO1 and Taxol genes enables biosynthesis of baccatin III
Source: Nature. 2025 Jun 11;643(8071):582–92. doi: 10.1038/s41586-025-09090-z (PMC12240809; doi:10.1038/s41586-025-09090-z)
Supplement: Supplementary file 2 — Reporting Summary [file 41586_2025_9090_MOESM2_ESM.pdf]

nature portfolio

Corresponding author(s): Elizabeth Sattely

Last updated by author(s): Mar 19, 2025

Reporting Summary

Nature Portfolio wishes to improve the reproducibility of the work that we publish. This form provides structure for consistency and transparency in reporting. For further information on Nature Portfolio policies, see our Editorial Policies and the Editorial Policy Checklist.

Statistics

For all statistical analyses, confirm that the following items are present in the figure legend, table legend, main text, or Methods section.

n/a	Confirmed	
☐	☒	The exact sample size (*n*) for each experimental group/condition, given as a discrete number and unit of measurement
☐	☒	A statement on whether measurements were taken from distinct samples or whether the same sample was measured repeatedly
☐	☒	The statistical test(s) used AND whether they are one- or two-sided<br>*Only common tests should be described solely by name; describe more complex techniques in the Methods section.*
☒	☐	A description of all covariates tested
☐	☒	A description of any assumptions or corrections, such as tests of normality and adjustment for multiple comparisons
☐	☒	A full description of the statistical parameters including central tendency (e.g. means) or other basic estimates (e.g. regression coefficient) AND variation (e.g. standard deviation) or associated estimates of uncertainty (e.g. confidence intervals)
☐	☒	For null hypothesis testing, the test statistic (e.g. *F*, *t*, *r*) with confidence intervals, effect sizes, degrees of freedom and *P* value noted<br>*Give P values as exact values whenever suitable.*
☒	☐	For Bayesian analysis, information on the choice of priors and Markov chain Monte Carlo settings
☒	☐	For hierarchical and complex designs, identification of the appropriate level for tests and full reporting of outcomes
☐	☒	Estimates of effect sizes (e.g. Cohen's *d*, Pearson's *r*), indicating how they were calculated

Our web collection on statistics for biologists contains articles on many of the points above.

Software and code

Policy information about availability of computer code

Data collection	GCMS and LCMS data is collected with Agilent 7820MS/Enhanced MassHunter and Agilent MassHunter Workstation Data Acquisition version 10.1, respectively.<br>Sony SH800 Cell Sorter Software v2.2.4<br>FlowJo v10
Data analysis	Routine data compilation was performed in Microsoft Excel (version 16.8). GCMS data analysis was performed with Agilent MassHunter Qualitative Analysis B.07.00. LCMS data analysis was performed with Agilent MassHunter Qualitative Analysis 10.0. Bar graphs were plotted using GraphPad Prism 10. NMR data were processed and visualized on MestReNova vl4.3.l. Molecular weight calculation and chemical structural visualization were conducted with Chem Draw Professional v22.2.0. Python code used for transcriptomic and untargeted metabolomic analysis will be deposited on GitHub. Published transcriptome datasets were cleaned with trimmomatic and mapped to the Taxus chinensis genome with STARmap.Ambient RNA was removed with cellbender (v.0.3.0) 57. Using the doubletdetection (v.4.2) library58, doublets were removed, as well as cells with outlier numbers of reads or where most reads were the most expressed genes (pct_counts_in_top_20_genes < 25).

For manuscripts utilizing custom algorithms or software that are central to the research but not yet described in published literature, software must be made available to editors and reviewers. We strongly encourage code deposition in a community repository (e.g. GitHub). See the Nature Portfolio guidelines for submitting code & software for further information.

Data

Policy information about availability of data

All manuscripts must include a data availability statement. This statement should provide the following information, where applicable:

- Accession codes, unique identifiers, or web links for publicly available datasets
- A description of any restrictions on data availability
- For clinical datasets or third party data, please ensure that the statement adheres to our policy

Raw and processed single-nuclei transcriptome data have been deposited at NCBI Gene Expression Omnibus (accession GSE292840). The bulk RNA-seq data from six previous studies for comparison were downloaded from NCBI (accession PRJNA493167, PRJNA251671, PRJNA733140, PRJNA427840, PRJNA497542, PRJNA499080, PRJNA86408). Python code used for transcriptomic and metabolomic analysis are deposited on GitHub (https://github.com/mcclune/nature2025). The raw NMR free induction decay (FID) data of individual compounds have been deposited in the Natural Products Magnetic Resonance Database (np-mrd.org) with the following ID: 4α,20-epoxy-taxadien-5α-ol (2'd, NP0350670), 4α,20-epoxy-5α-hydroxy-taxadien-13-one (NP0350671), 5α,13α-diacetoxy-taxadiene (NP0350849), taxusin (6, NP0341906), 13β-taxusin (6', NP0341907), 1β-hydroxytaxusin (6-O1, NP0341908), 15-hydroxy-11(15→1)abeo-taxusin (6-O2, NP0341909); the processed NMR data are shown in Fig. S23-65 and Table S3-10.

Research involving human participants, their data, or biological material

Policy information about studies with human participants or human data. See also policy information about sex, gender (identity/presentation), and sexual orientation and race, ethnicity and racism.

Reporting on sex and gender	N/A
Reporting on race, ethnicity, or other socially relevant groupings	N/A
Population characteristics	N/A
Recruitment	N/A
Ethics oversight	N/A

Note that full information on the approval of the study protocol must also be provided in the manuscript.

Field-specific reporting

Please select the one below that is the best fit for your research. If you are not sure, read the appropriate sections before making your selection.

☒ Life sciences ☐ Behavioural & social sciences ☐ Ecological, evolutionary & environmental sciences

For a reference copy of the document with all sections, see nature.com/documents/nr-reporting-summary-flat.pdf

Life sciences study design

All studies must disclose on these points even when the disclosure is negative.

Sample size	All experiments in this manuscript were conducted with a sample size of at least three to ensure minimal statistical power for analysis.
Data exclusions	No data were excluded during analysis.
Replication	All experiments were replicated at least once, and in most circumstances, in greater than three independent experiments.
Randomization	Randomization is not relevant to the experiments of this manuscript. The various experimental conditions were specifically defined to probe for the function of distinct enzymes, and there was no random assigning of samples to experiment groups.
Blinding	Blinding was not relevant to data collection as the metabolic data were acquired in an unbiased manner on automatic LCMS/GCMS instruments, where all detectable metabolites were measured without prior assumptions.

Reporting for specific materials, systems and methods

We require information from authors about some types of materials, experimental systems and methods used in many studies. Here, indicate whether each material, system or method listed is relevant to your study. If you are not sure if a list item applies to your research, read the appropriate section before selecting a response.

Materials & experimental systems

n/a	Involved in the study	
☐	☒	Antibodies
☒	☐	Eukaryotic cell lines
☒	☐	Palaeontology and archaeology
☒	☐	Animals and other organisms
☒	☐	Clinical data
☒	☐	Dual use research of concern
☐	☒	Plants

Methods

n/a	Involved in the study	
☒	☐	ChIP-seq
☐	☒	Flow cytometry
☒	☐	MRI-based neuroimaging

Antibodies

Antibodies used	Mouse monoclonal anti-VS antibody (SVS-Pkl) (Invitrogen #R960-2S) for detection of VS-tagged proteins by immunoblot in the Nicotiana benthamiana gene expression system used.<br>HA tag horseradish peroxidase-conjugated antibody (Biotechne #HAM0601) for detection of HA-tagged proteins by immunoblot in Nicotiana benthamiana. Dilution for each antibody is indicated in the method.
Validation	The mouse monoclonal anti-VS antibody (Invitrogen #R960-2S) is validated by the manufacturer to be specific to VS-tagged proteins by immunoblot, immunofluorescence microscopy, and functional analysis of the antibody against a fusion protein containing a VS epitope. Certificates of analysis and antibody validation are available on the manufacturers website (https://www.thermofisher.com/antibody/product/VS-Tag-Antibodyclone-SVS-Pkl-Monoelonal/R960-25)<br>The HA tag horseradish peroxidase-conjugated antibody (Biotechne #HAM0601) is validated by the manufacturer to be specific to HA-tagged proteins via immunoblot. Certificates of analysis and antibody validation are available on the manufacturers website (https://www.rndsystems.com/products/ha-tag-horseradish-peroxidase-conjugated-antibody-1049f _ham 0601)

Dual use research of concern

Policy information about dual use research of concern

Hazards

Could the accidental, deliberate or reckless misuse of agents or technologies generated in the work, or the application of information presented in the manuscript, pose a threat to:

No	Yes	
☒	☐	Public health
☒	☐	National security
☒	☐	Crops and/or livestock
☒	☐	Ecosystems
☒	☐	Any other significant area

Experiments of concern

Does the work involve any of these experiments of concern:

No	Yes	
☒	☐	Demonstrate how to render a vaccine ineffective
☒	☐	Confer resistance to therapeutically useful antibiotics or antiviral agents
☒	☐	Enhance the virulence of a pathogen or render a nonpathogen virulent
☒	☐	Increase transmissibility of a pathogen
☒	☐	Alter the host range of a pathogen
☒	☐	Enable evasion of diagnostic/detection modalities
☒	☐	Enable the weaponization of a biological agent or toxin
☒	☐	Any other potentially harmful combination of experiments and agents

Plants

Seed stocks: Nicotiana benthamiana plants used in this study are a gift from the Mudgett lab (Stanford) and propagated in house. Taxus plants are obtained from FastGrowingTrees.

Novel plant genotypes: No novel plant genotype was produced. Only Agrobacterium-mediated transient expression was used in this manuscript.

Authentication: N/A

Flow Cytometry

Plots

Confirm that:

☒ The axis labels state the marker and fluorochrome used (e.g. CD4-FITC).

☒ The axis scales are clearly visible. Include numbers along axes only for bottom left plot of group (a 'group' is an analysis of identical markers).

☒ All plots are contour plots with outliers or pseudocolor plots.

☒ A numerical value for number of cells or percentage (with statistics) is provided.

Methodology

Sample preparation: Nuclei were extracted as described in Methods section, resuspended in 1 ml NIB with 5 ng/ul 4,6-diamidino-2-phenylindole and 5 ng/ul propidium iodide and sorted on a Sony SH800 cell sorter with a 70 µm chip

Instrument: Sony SH800

Software: Sony SH800 software used during sorting and FlowJo vl0 was used for post-sorting data analysis.

Cell population abundance: For single-nuclei sequencing experiments, 150,000-200,000 nuclei were sorted prior to sequencing. During initial experiments, nuclei were examined under the microscope determine that that they were still spherical and intact. Nuclei quality could also be determined by the narrow width of the peak in DAPI and PI channels, indicating homogeneous nucleus size. Nuclei were extremely abundant (>30% of particles in sorted populations).

Gating strategy: Nuclei are gated on three subsequent gates: (i) size selection using forward scatter (FSC) vs side scatter (SSC), (ii) singlet selection using PI fluorescence height and width, and (iii) co-staining with DAPI and PI to identify clean nuclei.

☒ Tick this box to confirm that a figure exemplifying the gating strategy is provided in the Supplementary Information.
